# Quality of life and symptoms of pain in patients with endometriomas compared to those with other endometriosis lesions: a cross-sectional study

**DOI:** 10.1186/s12905-024-02919-1

**Published:** 2024-01-27

**Authors:** Fleur Serge Kanti, Valérie Allard, Sarah Maheux-Lacroix

**Affiliations:** Centre Hospitalier Universitaire de Québec - Université Laval, Quebec City, Quebec, Canada

**Keywords:** Endometriosis, Endometrioma, Quality of life, Endometriosis Health Profile-30, Pelvic pain, Infertility

## Abstract

**Background:**

Endometriomas are genetically distinct from other endometriosis lesions and could be associated with a predisposition to excessive inflammation. However, differences in clinical presentation between types of endometriosis lesions have not been fully elucidated. This study aimed to investigate the quality of life and pain scores of patients with endometriomas compared to those with other types of endometriosis lesions.

**Methods:**

A cross-sectional observational study was conducted between January 2020 and August 2023. Patients diagnosed with endometriosis completed the Endometriosis Health Profile 30 pain subscale questionnaire for their quality of life score and rated their endometriosis-associated pain symptoms using an 11-point numerical rating scale. The data were analyzed for comparison through multivariate linear regression models.

**Results:**

A total of 248 patients were included and divided into endometrioma (81, 33%) and nonendometrioma (167, 67%) groups. The mean age of the patients was 37.1 ± 7.5 years. Most participants were Canadian or North American (84%). One-third of the patients reported experiencing up to four concurrent pain symptoms. The most reported pain included deep dyspareunia (90%), chronic pelvic pain (84%) and lower back pain (81%). The mean quality of life score was 45.9 ± 25.9. We observed no difference in quality of life scores between patients with and without endometriomas. Patients with endometriomas had lower mean scores for deep dyspareunia (0.8; 95% CI [0 to 1.5]; *p* = 0.049) and higher mean scores for superficial dyspareunia (1.4; 95% CI [0.2 to 2.6]; *p* = 0.028). Comorbid infertility (*p* = 0.049) was a factor that modified superficial dyspareunia intensity in patients with endometriomas.

**Conclusion:**

In patients with endometriosis, evidence was insufficient to conclude that the presence of endometriomas was not associated with a greater or lesser quality of life, but differences in specific symptoms of dyspareunia were identified.

**Supplementary Information:**

The online version contains supplementary material available at 10.1186/s12905-024-02919-1.

## Background

Endometriosis is a common gynecological disorder that affects millions of women worldwide. It is characterized by the presence of endometrial tissue outside of the uterus [1]. No current cure exists for endometriosis. Treatment typically involves the use of pain-relief medications, hormonal therapy, and surgical removal of lesions [1, 2].

Endometrioma, which is the presence of an ovarian mass arising from ectopic endometrial tissue, is one of the most common manifestations of endometriosis [1]. Recently, the largest ever-round systems biological research on the genetics of endometriosis revealed that endometrioma is genetically distinct from other types of endometriosis and indicated that there may be a genetic predisposition to excessive inflammation in people with this condition [3]. The impact of endometriosis on quality of life has been extensively researched [4–7]. Patients with endometriosis were typically compared to symptomatic or asymptomatic control women without endometriosis from general or disease-specific populations. Studies have confirmed a reduced quality of life in women diagnosed with endometriosis compared with women who are not diagnosed with endometriosis, with women experiencing significantly lower levels of physical, mental, and social functioning and well-being [2, 8–14]. However, there is limited understanding of the variation in impairment levels experienced by patients with different types of endometriosis. Moreover, reducing pain or improving quality of life is a primary goal of endometriosis treatment, and the European Society of Human Reproduction and Embryology has suggested studying how surgery impacts pain and quality of life parameters in different subtypes of endometriosis [15]. Furthermore, previous studies have assessed quality of life using generic or nondisease-specific tools (most of the items were derived from clinicians and/or were scales taken from generic health status questionnaires) and endometriosis-specific questionnaires (patient-generated instruments) [4–6]. Disease-specific instruments are essential for assessing all aspects of chronic diseases [16]. Additionally, the American Society for Reproductive Medicine and the European Society of Human Reproduction and Embryology recommend the EHP-30 for use as a secondary outcome measure in clinical trials to assess endometriosis-associated pain [17]. Thus, there is a need for endometriosis research to better quantify and compare the quality of life and pain experienced by different women across endometriosis subtypes and to use disease-specific instruments that can produce better clinical results and more meaningful changes in patients’ lives.

In the present study, the endometriosis-specific questionnaire titled Endometriosis Health Profile-30 (EHP-30) [18] was used to compare the quality of life among patients with endometriomas and those with other types of endometriosis. Additionally, we sought to compare pain scores between patients with endometriomas and those with other types of endometriosis.

## Materials and methods

### Study design and participants

Since January 2020, a cohort has been established at the *Centre Hospitalier Universitaire de Québec-Université Laval* (Quebec, Canada). A list of patients scheduled for an initial or follow-up visit related to pelvic pain or endometriosis at the outpatient gynecology clinic was examined by a research professional to identify potential candidates. Eligible women were invited to participate and sent an email with a link to complete the online questionnaires and consent form before their visit to the clinic. Some participants could also be recruited by the medical team (attendings, students, nurses) during their consultation. Written informed consent was obtained from each participant prior to inclusion in the cohort. Ethical approval (reference number: 2020–4972) was obtained from the Research Ethics Committee of the *Centre Hospitalier Universitaire de Québec-Université Laval*.

The present study was a cross-sectional part of the ongoing prospective cohort and lasted until August 2023. The target population consisted of individuals who were invited to participate and who had a comprehensive history and physical examination. To be included in the study, an individual had to be diagnosed with endometriosis, regardless of the method of diagnosis (ultrasound and/or magnetic resonance imaging in the last year, visualization of lesions during surgery or histological confirmation); be aged 18 or older; and be able to read and understand the French language and complete a questionnaire. Participants were excluded if they did not report any symptoms of pain (as the primary outcome was assessed using questions relating to pain) or if their clinical evaluation did not allow for differentiation between endometrioma and other types of endometriosis, as imaging and histopathology data were not available at the time of the study.

**Fig. 1:**
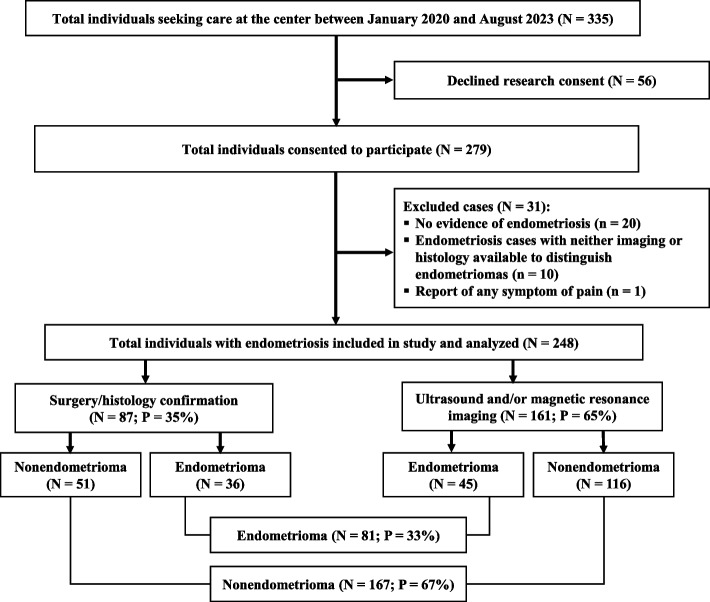
Study population flow chart N represents the frequency while P represents the percentage relative to the sample size (248)

The patients were divided into two groups based on the type of endometriosis: endometrioma and nonendometrioma groups. The endometrioma group consisted of all patients with endometriomas (ovarian cysts that contained dark, blood-stained fluid, often called chocolate cysts), regardless of the presence of other subtypes. The nonendometriosis group included superficial or peritoneal (lesions of various colors located on the surface of the peritoneum) and deep (nodular and fibrotic lesions that extend beyond the peritoneum and have the capacity to invade adjacent pelvic organs, such as the rectosigmoid, ureter or bladder) endometriosis lesions [19]. We used nonsurgical methods for diagnosis (based on symptoms and findings on physical examination and imaging) or laparoscopic visualization of endometriotic lesions with histopathological confirmation following recent guidelines [15, 20]. The study flow chart is shown in Fig. 1.

### Data collection

Participants self-completed a set of questionnaires (Supplemental file 1), which were supplemented by findings from a review of medical records. The main outcome measure was evaluated using the pain subscale of the validated 30-item Endometriosis Health Profile (EHP-30) questionnaire. This subscale consists of the first 11 items. The response options were “never,” “rarely,” “sometimes,” “often,” and “always.” The recall period was the last four weeks. This assessment examines how endometriosis-related pain affects quality of life [18]. The quality of life was assessed using the Canadian French version of the EHP-30 questionnaire [21]. The resulting score was then transformed into a scale ranging from 0 (indicating optimal quality of life) to 100 (indicating worst quality of life) [18]. The EHP-30 score was also categorized as impaired (score ≥ 75th centile of the EHP-30 score population distribution) or best (score < 75th centile of the EHP-30 score population distribution) quality of life [22]. The secondary outcomes included reports in the last three months of superficial dyspareunia (pain on penetration at the vaginal entrance during intercourse), deep dyspareunia (pain on deep penetration of the vagina during intercourse), dysmenorrhea (painful menstrual cramps), dyschezia (painful bowel movements), lower back pain and chronic pelvic pain (pelvic pain other than pain associated with sexual intercourse, painful menstrual cramps, painful bowel movements and lower back pain). Participants rated these symptoms according to intensity using an 11-point numerical rating scale (NRS) recommended for endometriosis research [17]. The NRS scores ranged from 0 (indicating no pain) to 10 (indicating the worst pain imaginable) [17]. The pain NRS scores were further categorized as severe (7–10) or mild-moderate (1–6) [22].

The sociodemographic variables were age, ethnicity, smoking status, employment status, marital status, educational level and annual income (Canadian dollars). The variables related to the disease, medical or obstetric history and physical examination consisted of the presence of fibroma or adenomyosis, age at menarche, years since onset of symptoms, presentation of concurrent symptoms of pain (1–4/5–6), comorbid infertility, gravida, parity, vaginal birth, cesarean section, use of hormonal treatment within the last three months, classes of hormones used in the last three months, use of painkillers, use of antidepressive drugs and body mass index (kg/m^2^). The psychological variables [23] included moderate symptoms linked to depression (score ≥ 10 on the validated Patient Health Questionnaire-9 [PHQ-9]) [24]; anxiety (score ≥ 10 on the validated Generalized Anxiety Disorder-7 [GAD-7] questionnaire) [25]; and pain catastrophizing (score ≥ 75th centile of the validated Pain Catastrophizing Scale [PCS], which includes questions about magnification, rumination, and helplessness) [26]. To discriminate between individuals with significant central contributors to their pain and those without, we administered the Central Sensitization Inventory (CSI) (a score ≥ 40 suggests patients with endometriosis with pain contributors related to central nervous system sensitization) [27].

### Statistical analysis

Our statistical analysis was completed using R software (version 4.3.1) [28]. Continuous data are presented as the mean ± standard deviation and/or median and interquartile or minimum–maximum range. Categorical data are reported as the frequency (percentage). Comparisons were performed between individuals with endometriomas and those without endometriomas using two-sample Wilcoxon–Mann–Whitney tests for continuous variables and chi-square tests of independence or Fisher's exact tests for categorical variables when appropriate.

Multivariate linear regression models were used to compare the primary (EHP-30 score for quality of life) and secondary (NRS score for symptoms of pain) outcomes between the endometrioma and nonendometrioma groups. The models were adjusted for age, body mass index, ethnicity, age of menarche, parity, education level, employment status, marital status, annual income, and hormone use in the last three months. Subgroup analyses were performed to determine whether differences in either quality of life scores or NRS pain scores (when comparing the endometrioma group to the nonendometrioma group) were associated with potential modification factors. To do so, an interaction term between each factor and variable of interest was added to the models. To aid in clinical interpretation, analyses with cutoff values were conducted using multivariate logistic regression models.

We estimated the point estimates, i.e., linear regression coefficients (β), as the mean differences for either quality of life or pain symptom scores between individuals with endometriomas and those without endometriomas and odds ratios (ORs), as the odds of a given outcome (impaired quality of life or severe pain symptom) occurring in the presence of endometriomas compared to the odds of that outcome in the absence of endometriomas. The estimates were supplemented by 95% confidence intervals (CIs). Values of *p* < 0.05 were considered to indicate statistical significance for all analyses. A *p* < 0.05 for the interaction term in subgroup analyses indicated that there was a statistically significant difference in the effect of the variable of interest on the outcome regarding the whole factor. No sample size calculation was conducted. Participants were included over a complete period to obtain a representative sample of patients with frequent hospital visits.

## Results

### Study population characteristics and outcome distributions

Out of the 335 individuals who were screened, 56 (17%) declined research consent, and a total of 248 (74%) met the criteria for inclusion in our study. The study sample consisted of 33% (81/248) of participants who were diagnosed with at least one endometrioma and 21% (51/248) and 73% (180/248) of those diagnosed with superficial and deep endometriosis, respectively. In terms of the diagnostic methods, 35% (161/248) of the participants underwent laparoscopic visualization of endometriotic lesions with histopathological confirmation (Fig. 1, Supplementary file 2: Table S1). Among the endometrioma participants who underwent surgery, the average number of endometrioma lesions was 1.5 ± 1.3 (10^–2^ m), and the average mean size was 5 ± 2.8 (10^–2^ m) (Supplementary file 2: Table S2).

The mean age of the patients was 37.1 ± 7.5 years, and the average body mass index was 26.6 ± 5.7 kg/m^2^. Most participants were Canadian or North American, accounting for 84% of the sample population. A large proportion of the participants (78%) had their first menstrual period at or before the age of 13, while more than half (54%) were nulliparous. One-third of the patients reported experiencing up to four pain symptoms simultaneously (34%). The nonendometrioma group participants were more likely to present a longer duration of pain symptoms (*p* = 0.047), a lower prevalence of presentation of up to four concurrent pain symptoms (*p* = 0.031) and a greater prevalence of adenomyosis (*p* = 0.02). There were significant associations between the type of endometriosis and the use of combined hormonal contraceptives (*p* = 0.028) and between the type of endometriosis and the method of diagnosis (*p* = 0.031). No statistically significant difference in any of the other variables was found between endometrioma patients and nonendometrioma patients (Table 1). On average, the quality of life score was 45.9 ± 25.9. The most reported pain symptoms included deep dyspareunia (90%), chronic pelvic pain (84%) and lower back pain (81%). In patients with severe symptoms (pain intensity of 7 or above), the most common symptoms reported were dysmenorrhea (66%), deep dyspareunia (58%), and chronic pelvic pain (50%) (Table 2).

**Table 1:** Main characteristics of the study population

**Variables**	**Endometriosis **^**a**^		**Total** (*N* = 248)	***p*-value** ^b^
	Endometrioma (*n* = 81)	Non-endometrioma (*n* = 167)		
Age (years)	37.0 ± 7.1	37.1 ± 7.8	37.1 ± 7.5	0.86
Age groups (years)				0.95
18 to 30	18 (22.2%)	35 (21.0%)	53 (21.4%)	
31 to 40	36 (44.4%)	70 (41.9%)	106 (42.7%)	
41 to 50	25 (30.9%)	56 (33.5%)	81 (32.7%)	
51 to 54	2 (2.5%)	6 (3.6%)	8 (3.2%)	
Body mass index (kg/m^2^)	26.4 ± 5.6	26.7 ± 5.8	26.6 ± 5.7	0.83
Body mass index groups (kg/m^2^)				0.14
Underweight (< 18.49)	5 (6.2%)	3 (1.8%)	8 (3.2%)	
Normal (18.5 to 24)	31 (38.3%)	80 (48.2%)	111 (44.9%)	
Overweight (25 to 29.99)	26 (32.1%)	41 (24.7%)	67 (27.1%)	
Obese (≥ 30)	19 (23.5%)	42 (25.3%)	61 (24.7%)	
Ethnicity				0.70
Canadian/North American	69 (85.2%)	139 (83.2%)	208 (83.9%)	
Other than above ^c^	12 (14.8%)	28 (16.8%)	40 (16.1%)	
Educational level				0.15
University	40 (50.0%)	62 (37.1%)	102 (41.3%)	
Collegial	21 (26.3%)	59 (35.3%)	80 (32.4%)	
Elementary/secondary or preferred not to answer	19 (23.8%)	46 (27.5%)	65 (26.3%)	
In employment	71 (87.7%)	133 (79.6%)	204 (82.3%)	0.12
Annual income (Canadian dollars)				0.54
≤ 19,999 or preferred not to answer	11 (13.9%)	30 (18.5%)	41 (17.0%)	
20,000 to 59,999	22 (27.8%)	45 (27.8%)	67 (27.8%)	
60,000 to 99,999	18 (22.8%)	43 (26.5%)	61 (25.3%)	
≥ 100,000	28 (35.4%)	44 (27.2%)	72 (29.9%)	
Marital status				0.40
Dating or married or common law	66 (81.5%)	143 (85.6%)	209 (84.3%)	
Other than above ^d^	15 (18.5%)	24 (14.4%)	39 (15.7%)	
Smoking	5 (17.9%)	15 (29.4%)	20 (25.3%)	0.26
Menarche				0.32
≤ 13 years old	60 (74.1%)	133 (79.6%)	193 (77.8%)	
> 13 years old or not sure	21 (25.9%)	34 (20.4%)	55 (22.2%)	
Nulliparous	46 (56.8%)	88 (52.7%)	134 (54.0%)	0.54
Gravida	2.1 ± 1.1	2.7 ± 1.8	2.5 ± 1.7	0.18
Parity	0.8 ± 1.0	0.9 ± 1.2	0.9 ± 1.1	0.32
Vaginal birth	0.5 ± 0.9	0.6 ± 1.0	0.6 ± 1.0	0.45
Cesarean section	0.2 ± 0.5	0.3 ± 0.7	0.3 ± 0.7	0.79
Comorbid infertility	13 (16.0%)	20 (12.0%)	33 (13.3%)	0.38
Years since onset of pain symptoms	9 (3—18)	12 (5—22)	11 (5—20)	0.047
Reporting up to four concurrent pain symptoms	31 (44.3%)	40 (29.2%)	71 (34.3%)	0.031
Hormones use within the last three months	41 (64.1%)	90 (60.0%)	131 (61.2%)	0.58
Classes of hormones used in the last three months ^e^				
Combined hormonal contraceptives	4 (4.9%)	24 (14.4%)	28 (11.3%)	0.028
Progestins	21 (25.9%)	53 (31.7%)	74 (29.8%)	0.35
GnRH agonists and antagonists	9 (11.1%)	16 (9.6%)	25 (10.1%)	0.71
Painkillers use	50 (61.7%)	118 (70.7%)	168 (67.7%)	0.16
Anti-depressive drugs use	15 (68.2%)	37 (59.7%)	52 (61.9%)	0.48
Diagnosis method				0.031
Imaging ^f^	45 (55.6%)	116 (69.5%)	161 (64.9%)	
Histology	36 (44.4%)	51 (30.5%)	87 (35.1%)	
Adenomyosis	2 (4.9%)	12 (21.8%)	14 (14.6%)	0.020
Fibroma	12 (57.1%)	38 (79.2%)	50 (72.5%)	0.060
Pain catastrophizing (PCS ≥ 27)	19 (23.5%)	48 (28.7%)	67 (27.0%)	0.38
Moderate anxiety (GAD-7 ≥ 10)	6 (23.1%)	14 (23.0%)	20 (23.0%)	0.99
Moderate depression (PHQ-9 ≥ 10)	9 (34.6%)	21 (34.4%)	30 (34.5%)	0.99
Central component of pain (CSI ≥ 40)	5 (11.1%)	15 (17.9%)	20 (15.5%)	0.31

*Abbreviations*: n, frequency per group; N, sample size; *GnRH* gonadotropin-releasing hormone, *PCS* Pain Catastrophizing Scale, *GAD-7* Generalized Anxiety Disorder, *PHQ-9* Patient Health Questionnaire-9, *CSI* Central Sensitization Inventory

^a^Values are given in mean ± standard deviation, median (interquartile range), and frequency (percentage)

^b^Wilcoxon rank sum test for continuous variables; and Pearson’s Chi-squared test or Fisher’s exact test otherwise

^c^Autochthon-Inuit, Central and South America, European, African, Asian, mixed and others

^d^Single, separated, divorced and widowed

^e^Patients may use at least one hormone in at least one hormone class. Hormone classes were described separately

^f^Imaging modalities (ultrasound and/or magnetic resonance imaging) were used in combination with symptoms and findings on physical examination

**Table 2:** Distribution of the quality of life and pain scores of patients with different endometriosis lesions

Outcomes ^a^	Endometriosis ^b^		Total (*N* = 248)
	**Endometrioma** **(*n* = 81)**	**Non endometrioma** **(*n* = 167)**	
**Quality of life**			
EHP-30 score	42.4 ± 25.2	47.6 ± 26.2	45.9 ± 25.9
Impaired (EHP-30 score ≥ 66 ^c^)	21 (25.9%)	48 (28.7%)	69 (27.8%)
Not available	0	0	0
**Superficial dyspareunia**			
Have reported	37 (49.3%)	98 (64.1%)	135 (59.2%)
Intensity	5.0 ± 2.6	4.1 ± 2.6	4.3 ± 2.6
Severe (intensity ≥ 7)	10 (27.0%)	20 (20.4%)	30 (22.2%)
Not available	6	14	20
**Deep dyspareunia**			
Have reported	68 (89.5%)	138 (90.2%)	206 (90.0%)
Intensity	6.0 ± 2.5	6.7 ± 2.2	6.5 ± 2.3
Severe (intensity ≥ 7)	35 (51.5%)	85 (61.6%)	120 (58.3%)
Not available	5	14	19
**Dysmenorrhea**			
Have reported	57 (75.0%)	124 (81.6%)	181 (79.4%)
Intensity	6.6 ± 2.7	7.4 ± 2.3	7.1 ± 2.4
Severe (intensity ≥ 7)	36 (63.2%)	83 (66.9%)	119 (65.7%)
Not available	5	15	20
**Dyschezia**			
Have reported	50 (61.7%)	135 (81.8%)	185 (75.2%)
Intensity	4.9 ± 2.4	5.0 ± 2.5	5.0 ± 2.4
Severe (intensity ≥ 7)	14 (28.0%)	39 (28.9%)	53 (28.6%)
Not available	0	2	2
**Lower back pain**			
Have reported	64 (79.0%)	137 (82.0%)	201 (81.0%)
Intensity	5.6 ± 2.3	6.4 ± 2.1	6.1 ± 2.2
Severe (intensity ≥ 7)	24 (37.5%)	65 (47.4%)	89 (44.3%)
Not available	0	0	0
**Chronic pelvic pain**			
Have reported	64 (79.0%)	143 (85.6%)	207 (83.5%)
Intensity	6.3 ± 2.0	6.6 ± 2.3	6.5 ± 2.2
Severe (intensity ≥ 7)	27 (42.2%)	76 (53.1%)	103 (49.8%)
Not available	0	0	0

*Abbreviations*: *EHP-30*, Endometriosis Health Profile-30

^a^Pain intensity is the numerical rating scale score ranging from 0 (indicating no pain) to 10 (indicating the worst pain imaginable); the EHP-30 score ranges from 0 (indicating optimal quality of life) to 100 (indicating worst quality of life); each “Not available” row displays the number of missing values; each “Have reported” row denotes the number of cases that reported the corresponding symptom of pain

^b^Values are given in mean ± standard deviation and frequency (percentage)

^c^The value (66) corresponds to the 75^th^ centile of EHP-30 score population distribution

### Associations between quality of life and endometriomas

**Fig. 2:**
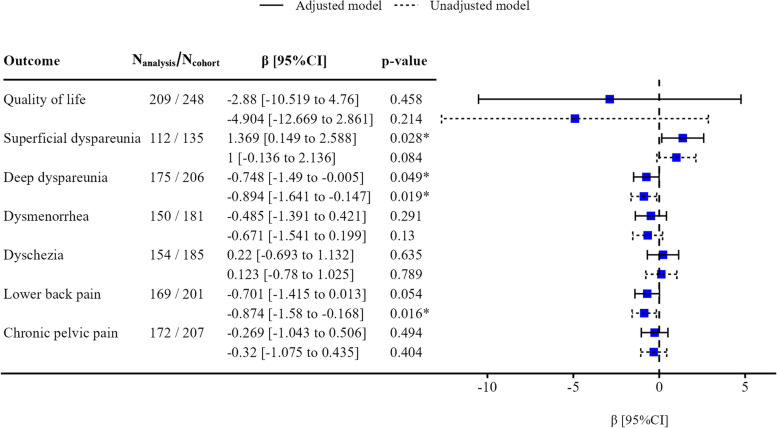
Quality of life and pain symptom scores of patients with different endometriosis lesions Abbreviations: CI, confidence interval Notes: N_analysis_ denotes the number of individuals available for analysis while N_cohort_ denotes the total number of individuals enrolled in the cohort. The differences between the adjusted and unadjusted models are the observations deleted due to missing data. β denotes linear regression coefficient (mean difference in quality of life or pain symptom scores between individuals with endometriomas and those without endometriomas). The vertical black dashed line represents the null value (zero) of the mean difference, indicating that the mean difference is significantly different from zero when the confidence interval does not include zero (equivalent to *p* < 0.05). The mean differences in quality of life score or pain symptom intensity are indicated by blue squares (point estimate values), with 95% confidence intervals delimited by black horizontal solid (adjusted model) or dashed (unadjusted model) lines. The models were adjusted for age, body mass index (kg/m^2^), ethnicity, age of menarche, parity, education level, employment status, marital status, annual income, and hormone use in the last three months. Stars highlight *p*-values less than 0.05

The unadjusted and adjusted analyses for quality of life scores indicated no difference between patients with and without endometriomas (mean difference of -4.9 (95% CI, [-12.7 to 2.9]; *p* = 0.21) and -2.9 (95% CI, [-10.5 to 4.8]; *p* = 0.46), respectively) (Fig. 2).

**Fig. 3:**
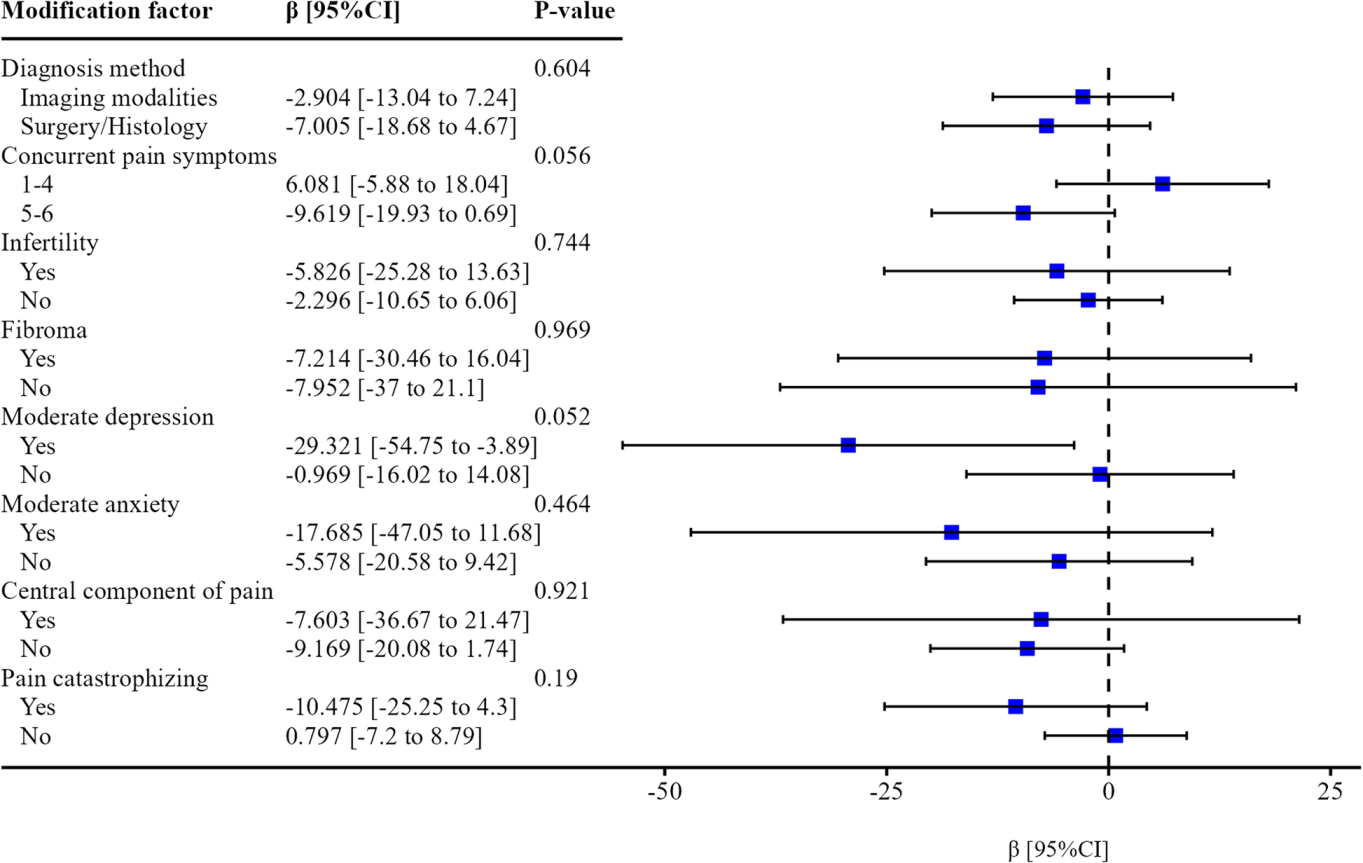
Subgroup analyses of the mean difference in the quality of life score Abbreviations: CI, confidence interval Notes: β denotes the linear regression coefficient (mean difference in the quality of life between individuals with endometriomas and those without endometriomas). The modification factors were the diagnostic method (imaging modalities/histology), concurrent pain symptoms (1–4/5–6), comorbid infertility (yes/no), presence of adenomyosis (yes/no), presence of fibroma (yes/no), moderate depression symptoms (yes/no), moderate anxiety symptoms (yes/no), pain catastrophizing (yes/no) and central component of pain (yes/no). The *P* value for each factor was used for determining the association between the quality of life score and the presence of endometrioma (modification factor if *P* < 0.05; not otherwise), allowing to compare the mean difference between modification factor levels. For each level of each factor, the mean difference in the quality of life score between patients with endometriomas and those with other types of endometriosis is indicated by the blue square (point estimate value), with the 95% confidence interval delimited by the black horizontal solid line. The vertical black dashed line represents the null value (zero) of the mean difference, indicating that the mean difference is significantly different from zero when the confidence interval does not include zero (equivalent to *p* < 0.05; not shown here). The models were adjusted for age, body mass index (kg/m^2^), ethnicity, age of menarche, parity, education level, employment status, marital status, annual income, hormone use in the last three months, and each modification factor using an interaction term with the endometriosis type variable

No statistically significant difference in quality of life score was found between participants with endometriomas and those without endometriomas across the modifying factors. When considering only participants reporting moderate symptoms of depression, there was a significantly lower mean quality of life score (29.3; 95% CI [3.9 to 54.8]) for patients with endometriomas than for those without endometriomas (Fig. 3).

### Associations between pain scores and endometriomas

Participants with endometriomas had significantly greater mean superficial dyspareunia scores (1.4; 95% CI, [0.2 to 2.6]; *p* = 0.028) and significantly lower mean deep dyspareunia scores (0.8; 95% CI, [0 to 1.5]; *p* = 0.049) according to the adjusted analyses than did those without endometriomas. For other pain symptoms (dysmenorrhea, dyschezia, lower back pain, chronic pelvic pain), there were no statistically significant differences in the means (Fig. 2).

Comorbid infertility (*p* = 0.049) was found to be a factor that influenced the results for superficial dyspareunia, with an average difference of 4.6 (95% CI [1.2 to 8.1]) if infertility occurred versus 0.9 (95% CI [-0.3 to 2.2]) otherwise (Supplementary file 2: Fig. S1). The central component of pain (*p* = 0.041) emerged as a factor that modified the potential average difference in scores for chronic pelvic pain (2.54; 95% CI, [-0.4 to 5.5] if the central component of pain was involved versus -0.7; 95% CI, [-1.8 to 0.3] otherwise (Supplementary file 2: Fig. S2).

Considering solely the levels of modifying factors, statistically significant mean score differences were found between participants with endometriomas and those without endometriomas (i.e., 95% CIs did not include zero). These differences were observed in individuals reporting up to four concurrent symptoms of pain, comorbid infertility, moderate symptoms of anxiety and pain catastrophizing for superficial dyspareunia (Supplementary file 2: Fig. S1), absence of a central component in pain experience and no pain catastrophizing for deep dyspareunia (Supplementary file 2: Fig. S3), and presence of a central component in pain experience and no pain catastrophizing for lower back pain (Supplementary file 2: Fig. S4). No such differences were identified for chronic pelvic pain (Supplementary file 2: Fig. S2), dysmenorrhea (Supplementary file 2: Fig. S5), or dyschezia (Supplementary file 2: Fig. S6).

### Odds ratios of impaired quality of life and severe pain symptoms

The OR for impaired quality of life (patients with endometriomas versus patients without endometriomas) was 1.05 (95% CI [0.5 to 2.19]; *p* = 0.89). For symptoms of pain, the OR ranged from 0.52 (95% CI, [0.25 to 1.08]; *p* = 0.08) for severe deep dyspareunia to 2.11 (95% CI, [0.60 to 7.43]; *p* = 0.24). Endometrioma was not associated with impaired quality of life or any severe symptoms of pain, but the CIs in the adjusted analyses indicated that the data were compatible with an OR greater than or less than 1 (Supplementary file 2: Fig. S7).

## Discussion

In this study, we found insufficient evidence to conclude that there is no real difference in quality of life between patients with and without endometriomas. In terms of pain scores, there were differences in deep and superficial dyspareunia. Comorbid infertility was a factor that significantly modified the association between superficial dyspareunia and the presence of endometriomas.

Since no current cure exists for endometriosis, this research supports the development of targeted treatments appropriate for groups with similar clinical experience or for a given subpopulation's underlying biological differences [3, 29]. The present study focused on the quality of life of patients with endometriosis who had different types of lesions. Specifically, endometriosis, as a chronic inflammatory illness, affects quality of life rather than just considering the presence or absence of pain symptoms. Indeed, the study involved people who had experienced at least one symptom of pain. These findings add to the limited evidence in terms of the variation in pain and quality of life across endometriosis types. These findings echo earlier research indicating that endometriosis, regardless of type, significantly impacts patients’ quality of life and pain experience. Patients with endometriosis seem to have an overall impaired quality of life compared to patients from the general population, including daily tasks, marital or sexual relationships, social life, and employment, as well as physical and psychological aspects of life [2, 8–14]. In contrast to these previous studies, it should be emphasized that we focused on evaluating quality of life, specifically about the experience of pain only.

**Fig. 4:**
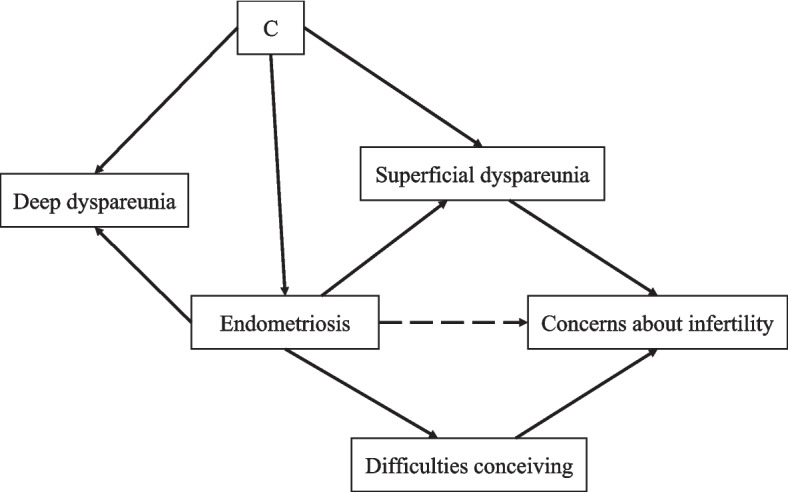
Directed acyclic graph illustrating simplified relationships of superficial and deep dyspareunia with endometriosis Abbreviations: C, confounding factors considered in the study (age, body mass index, ethnicity, age of menarche, parity, education level, employment status, marital status, annual income, and hormone use in the last three months) Notes: Solid arrows represent direct associations between variables. The dashed arrow represents a potential relationship

While patients with endometriomas had significantly higher scores for superficial dyspareunia (modified by comorbid infertility), lower scores were noted for deep dyspareunia. These findings suggest that the pain experienced by patients with endometriosis may vary by symptom and endometriosis subtype [30]. Overall, variations in the perception, nature, and intensity of pain among individuals can account for the differences observed in the results. Additionally, there may be associations between different pain symptoms that contribute to these variations (e.g., deep dyspareunia may be the exacerbation of preexisting chronic pelvic pain by deep penetration in some patients, and deep and superficial dyspareunia seem to cooccur in populations seeking tertiary care [31]). In Fig. 4, we briefly illustrate some of the factors associated with superficial and deep dyspareunia and endometriosis. The findings of superficial dyspareunia could be explained through concerns about infertility. There could be a direct pathway from endometriosis to concerns about infertility (endometriosis is associated with difficulties conceiving [32, 33], difficulties conceiving are significantly related to concerns about infertility [34], and women who are diagnosed with endometriosis are concerned about future infertility caused by endometriosis [35]). Superficial dyspareunia was also found to be significantly related to concerns about infertility [34]. Thus, superficial dyspareunia could be considered a mediator in the aforementioned pathway (considering the association between endometriosis and superficial dyspareunia that we identified). The positive association between endometrioma and superficial dyspareunia (found in the present study) and the (existing) positive association between superficial dyspareunia and concerns about infertility [34] could result in a positive association between endometrioma and concerns about infertility, suggesting that women with endometrioma may have more concerns about future fertility than those with other endometriosis lesions and even more so in cases of comorbid infertility (e.g., as the direct affected organs are ovaries, which have a significant function in reproduction). However, we are not aware of a direct comparison of women with different types of endometriosis to investigate this hypothesis. It should be highlighted that there are several potential causes of superficial dyspareunia, and this symptom often involves a combination of physical, psychological, and relational factors. Additionally, since pain may also be due to the anatomical distortion that endometriomas create in the pelvis [36], the localization and size of endometriotic foci may explain differences in the intensity of pain compared to that of other endometriotic lesions. The deep dyspareunia findings could be explained by direct contact with the affected structures (e.g., uterosacral ligaments, cul-de-sac and bladder). Endometriomas often cooccur with deep endometriosis [1]. In this study, the endometrioma group included all patients with endometriomas, regardless of the other subtypes. Our study highlights the complex impact of endometriosis and cooccurring conditions (such as depression) on quality of life, underscoring the importance of personalized care strategies, including medical, psychological, and nonmedical interventions such as physical therapy and lifestyle changes. The findings also emphasize the need for the development of targeted medical and surgical treatments to improve patient outcomes. The finding that associated conditions such as depression could significantly affect quality of life in endometrioma patients compared to nonendometrioma patients suggests that healthcare providers should consider diagnostic strategies to identify such conditions and involve them in the overall management approach. However, additional studies are needed to determine which factors explain the differences in quality of life among patients with endometriosis.

The strengths of this study included the use of a substantial sample size for the main analyses; the use of a validated measure for quality of life in the endometriosis population (the EHP-30, which was specifically developed and validated for quality of life assessment in women with endometriosis and is important for evaluating the perceptions of women’s disease impact and treatment effectiveness in clinical scenarios); and the use of appropriate and validated statistical methods to analyze the data. Moreover, we considered a wide range of modifying factors (such as adenomyosis, comorbid infertility, and psychological factors), which provides a more comprehensive view of the factors influencing quality of life and pain in endometriosis patients. Furthermore, the focus on comparing quality of life and pain experience between patients with endometriomas and patients with other types of endometriosis (comparisons among patients with endometriosis) is a distinct feature of this study and contributes unique findings to the research field. This study is limited by its cross-sectional design, which prevents the ability to establish cause‒effect relationships between different types of endometriosis and quality of life or pain experience. In addition, there are potential sources of bias that may impact the findings, and factors limiting external validity. First, since the data collection partly relied on self-report questionnaires, the findings might be subject to recall bias. This can arise due to several factors, such as memory limitations, selective memories and delays between the event and the survey. However, we assume that short recall periods (four weeks for quality of life and three months for pain intensity) can reduce the reliance on participants' retrospective recall. Thus, the recall bias effect on comparing outcomes between participants' groups should be negligible. Second, we included participants with various methods of diagnosing endometriosis, not all of whom underwent surgery, thus leading to potential variability in the anatomical extent of the disease. It is important to emphasize that an accurate diagnosis of endometriosis is challenging. The gold standard for diagnosis involves laparoscopic visualization with histopathological confirmation. However, recent international guidelines recommend the diagnosis and first-line management of endometriosis without laparoscopy, leading many women to receive a clinical diagnosis of endometriosis instead [15, 20]. Moreover, our analyses revealed that the diagnostic method was not a factor influencing the association between the type of endometriosis and quality of life (if present) or pain symptoms. Third, despite controlling for multiple variables, residual confounding is likely to remain. We did not consider medical conditions related to pelvic pain or commonly coexisting pain conditions (such as vulvodynia, irritable bowel syndrome and painful bladder syndrome) [37]; the use of painkillers or antidepressive drugs; menopausal transition; or other risk factors for endometriosis (such as low birth weight, short menstrual cycles and increased menstrual flow) [38, 39]. Next, residual confounding may still exist, potentially due to factors that are unknown and cannot be measured. Fourth, the participants were recruited from a specific clinical setting and geographical region, which might limit the representativeness of the findings to a wider population of women with endometriosis and the generalizability of the findings to the more diverse population of women with endometriosis worldwide. In particular, the fact that participants were enrolled in a tertiary care center could lead to a possible overrepresentation of women with moderate-to-severe endometriosis and limit the generalizability of the findings to clinics, primary care settings or general populations. Fifth, the psychometric properties of the Canadian French version of the questionnaires used in this study have not yet been assessed. There is a need to externally validate the questionnaires. It is noteworthy that, to date, it is the only available version of the original instrument in Canadian French, and it has previously been translated and cross-culturally adapted [21]. Finally, since our analyses were performed on data that were already available from an ongoing endometriosis cohort and no formal a priori calculation of sample size was conducted, the potential for type II error should be highlighted, i.e., detecting a null difference when a real difference is present.

Future research could benefit from a longitudinal design to determine changes in quality of life and pain scores over time, considering, for example, patients with a comprehensive histology diagnosis (allowing to accurately distinguish patients with endometrioma only and others with superficial or deep endometriosis only and to take into account the anatomical extent of the disease). This perspective will help to explore in more depth the relationships between different types of endometriosis, quality of life, specific pain experiences, and other influencing factors or cooccurring conditions. This could lead to more effective approaches to improve the quality of life of patients with endometriosis. Further exploration of personalized treatment plans based on the type of endometriosis and the patient's symptoms and assessments of quality of life could be valuable.

## Conclusion

In the studied sample of patients with endometriosis, there was insufficient evidence to conclude that the presence of endometriomas was not associated with a greater or lesser quality of life. Differences in specific symptoms of dyspareunia were identified and found to be modified by comorbid infertility for superficial dyspareunia.

### Supplementary Information

**Additional file 1: Supplementary file 1.** is the list of questionnaires completed by participants (in the ongoing prospective cohort from which the present study was conducted) and the initial clinical, physical examination, ultrasound, surgical and pathology forms. The questionnaires completed by participants included the Standard Endometriosis Patient Questionnaire of the Endometriosis Phenome (and Biobanking) Harmonisation Project from the World Endometriosis Research Foundation, translated from English and adapted to the cultural context in Canadian French for the purposes of the study. We provide these questionnaires in the native language without retranslation into English, as this better aligns with the study's context.

**Additional file 2: Table S1.** Frequency and percentage of endometriosis subtypes in the study sample. **Table S2.** Number and size of endometriomas in participants with surgical diagnosis. **Figure S1.** Subgroup analyses of mean difference in superficial dyspareunia intensity of patients with endometrioma compared to those with other types of endometriosis regarding potential modification factors. **Figure S2.** Subgroup analyses of mean difference in chronic pelvic pain intensity of patients with endometrioma compared to those with other types of endometriosis regarding potential modification factors. **Figure S3.** Subgroup analyses of mean difference in deep dyspareunia intensity of patients with endometrioma compared to those with other types of endometriosis regarding potential modification factors. **Figure S4.** Subgroup analyses of mean difference in lower back pain intensity of patients with endometrioma compared to those with other types of endometriosis regarding potential modification factors. **Figure S5.** Subgroup analyses of mean difference in dysmenorrhea intensity of patients with endometrioma compared to those with other types of endometriosis regarding potential modification factors. **Figure S6.** Subgroup analyses of mean difference in dyschezia intensity of patients with endometrioma compared to those with other types of endometriosis regarding potential modification factors. **Figure S7.** Impaired quality of life and severe pain symptoms of patients with endometriomas compared to those with other lesions of endometriosis.

## Data Availability

The datasets generated and/or analyzed during the current study are not publicly available due to privacy/ethical restrictions but anonymized data are available from the corresponding author (FSK) upon reasonable request and with permission from SML.

## Abbreviations

CI: Confidence interval
EHP-30: Endometriosis Health Profile 30
NRS: Numerical rating scale
OR: Odds ratio
