# Supplementary material for: Quality of life and symptoms of pain in patients with endometriomas compared to those with other endometriosis lesions: a cross-sectional study
Source: BMC Womens Health. 2024 Jan 27;24:72. doi: 10.1186/s12905-024-02919-1 (PMC10821264; doi:10.1186/s12905-024-02919-1)
Supplement: Supplementary file 1 — Additional file 1: Supplementary file 1. is the list of questionnaires completed by participants (in the ongoing prospective cohort from which the present study was conducted) and the initial clinical, physical examination, ultrasound, surgical and pathology forms. The questionnaires completed by participants included the Standard Endometriosis Patient Questionnaire of the Endometriosis Phenome (and Biobanking) Harmonisation Project from the World Endometriosis Research Foundation, translated from English and adapted to the cultural context in Canadian French for the purposes of the study. We provide these questionnaires in the native language without retranslation into English, as this better aligns with the study's context. [file 12905_2024_2919_MOESM1_ESM.pdf]

Quality of life and symptoms of pain in patients with endometriomas compared to those with other endometriosis lesions: a cross-sectional study

Authors and Affiliations

Fleur Serge Kanti[1*]

Valérie Allard[1]

Sarah Maheux-Lacroix[1]

1. Centre hospitalier universitaire de Québec - Université Laval, Quebec City, Quebec, Canada.

*** Corresponding Author**

Fleur Serge Kanti

Centre hospitalier universitaire de Québec - Université Laval

2705, boulevard Laurier

T0-64

Québec, Québec

Canada, G1V 4G2

Phone: +1 418 525 4444, # 46185

E-mail: serge.kanti@crchudequebec.ulaval.ca

SUPPLEMENTARY FILE 1

Supplementary file 1 is the list of questionnaires completed by participants (in the ongoing prospective cohort from which the present study was conducted) and the initial clinical, physical examination, ultrasound, surgical and pathology forms. The questionnaires completed by participants included the Standard Endometriosis Patient Questionnaire of the Endometriosis Phenome (and Biobanking) Harmonisation Project from the World Endometriosis Research Foundation, translated from English and adapted to the cultural context in Canadian French for the purposes of the study. We provide these questionnaires in the native language without retranslation into English, as this better aligns with the study's context.

Questionnaire initial - CLINIQUE

Veuillez compléter le questionnaire ci-dessous.

Merci!

QUESTIONNAIRE INITIAL - VOLET CLINIQUE

La complétion de ces questions est essentielle au bon suivi de vos symptômes par votre équipe traitante. Les réponses seront versées à votre dossier clinique. Ces données nous permettront de vous offrir une meilleure prise en charge.

Nous sommes conscients qu'il peut être difficile de répondre avec précision à certaines questions. Veuillez répondre aux meilleurs de vos connaissances.

Nom de famille, prénom ______________________

Numéro carte CHUL ______________________

Adresse courriel : ______________________

Quelle est votre date de naissance? ______________________
(Cliquer sur le calendrier et sélectionner votre date de naissance)

Date de complétion du questionnaire ______________________

Âge ______________________
(calculé avec les données ci-haut)

Quelles sont les trois premiers caractères de votre code postal? ______________________

Antécédents menstruels et hormones

À quel âge avez-vous eu vos premières menstruations?	◯ 8 ans ou moins ◯ 9<br>◯ 10 ◯ 11 ◯ 12 ◯ 13<br>◯ 14 ◯ 15 ◯ 16 ◯ 17 ans ou plus<br>◯ Je ne suis pas sûre ◯ Mes menstruations n'ont pas encore commencé
Avez-vous eu vos menstruations dans les 3 derniers mois? (Nous parlons des saignements nécessitant un tampon ou une serviette hygiénique et NON des pertes vaginales (traces) nécessitant seulement un protège-dessous)"	◯ Oui ◯ Non
Vos menstruations des 3 derniers mois ont-elles été régulières?	◯ Extrêmement régulières (début des menstruations 1-2 jours avant ou après la date attendue)<br>◯ Très régulières (début des menstruations 3-4 jours avant ou après la date attendue)<br>◯ Régulières (début des menstruations 5-7 jours avant ou après la date attendue)<br>◯ Plutôt irrégulières (début des menstruations 8-20 jours avant ou après la date attendue)<br>◯ Irrégulières (début des menstruations plus de 20 jours avant ou après la date attendue)
Combien de jours ont habituellement duré les saignements à chaque fois que vous avez eu vos menstruations dans les 3 derniers mois? (Sans compter les pertes vaginales/traces nécessitant seulement un protège-slip)"	◯ 1<br>◯ 2<br>◯ 3<br>◯ 4<br>◯ 5<br>◯ 6<br>◯ 7<br>◯ 8<br>◯ 9<br>◯ 10<br>◯ 11<br>◯ 12<br>◯ 13<br>◯ 14<br>◯ 15<br>◯ 16<br>◯ 17<br>◯ 18<br>◯ 19<br>◯ 20<br>◯ Trop irrégulier pour pouvoir le dire

Powered by REDCap

L'image ci-dessous montre des exemples d'abondance de saignements que vous pourriez constater toutes les quatre heures pendant vos menstruations.

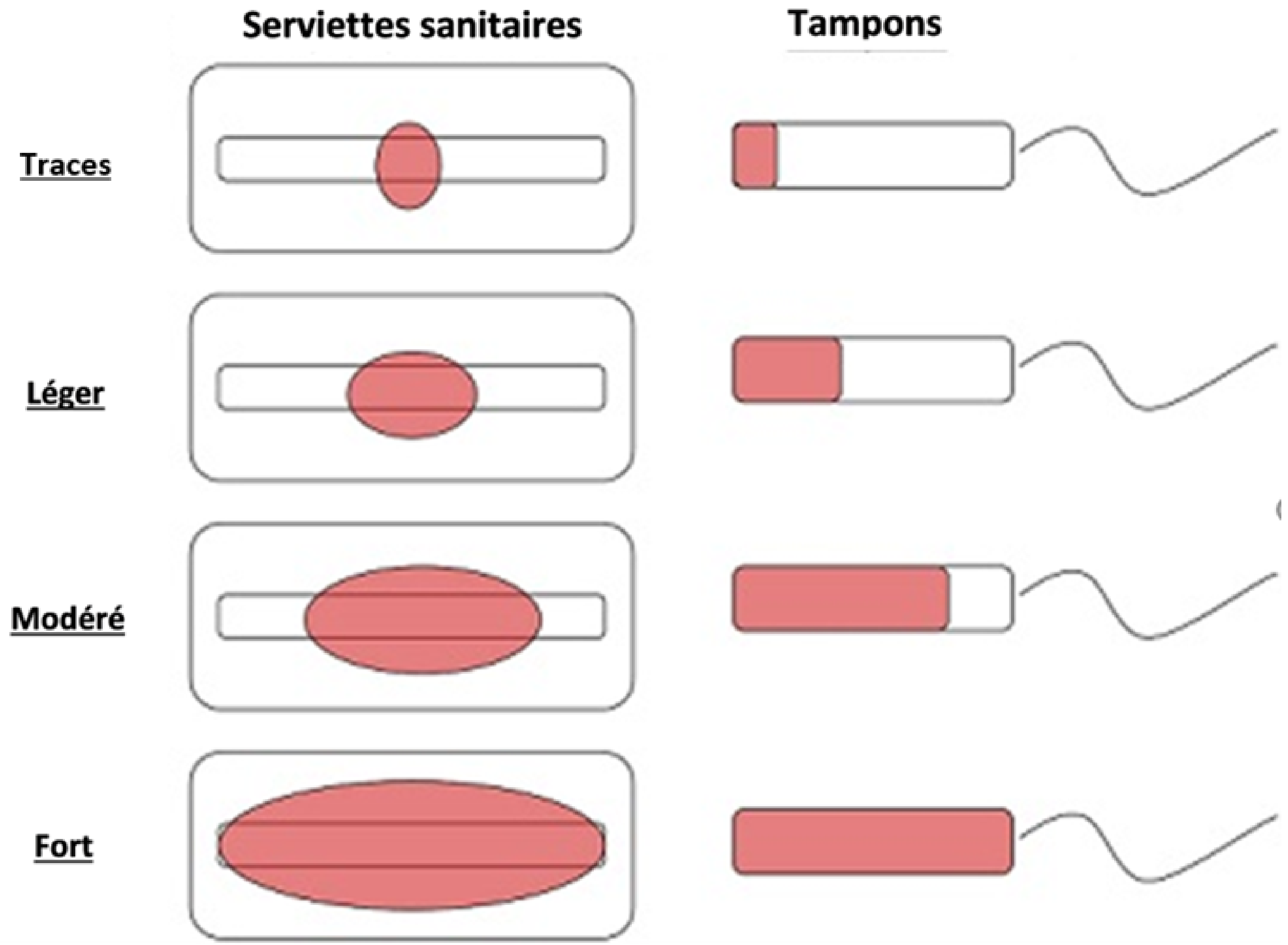

Veuillez préciser l'abondance des saignements que vous constatez habituellement toutes les quatre heures, au plus fort de vos menstruations et en moyenne pendant vos menstruations.

	Traces	Léger	Moyen	Fort (caillots/écoulement massif)
Au plus fort des menstruations	◯	◯	◯	◯
En moyenne pendant vos menstruations	◯	◯	◯	◯

Avez-vous déjà utilisé des hormones (ex. pilule contraceptive, timbres, anneau vaginal, injection, implant, stérilet, etc.) quelle qu'en soit la raison (acné, menstruations douloureuses ou irrégulières, contraception, traitements contre l'infertilité, etc.)?

◯ Oui ◯ Non

Combien d'hormones différentes avez-vous déjà utilisées?

____

Les questions suivantes portent sur toutes les hormones que vous avez déjà utilisées quelle qu'en soit la raison (acné, menstruations douloureuses ou irrégulières, contraception, traitements contre l'infertilité). Si vous ne vous souvenez pas du nom de l'hormone utilisée, veuillez inscrire dans la première colonne.

HORMONE #1

Nom de l'hormone (Par exemple : Alesse, Norlutate, etc.). Si vous ne vous souvenez pas du nom de l'hormone utilisée, veuillez inscrire . ________________________________

Type d'hormone

- ◯ Pilule contraceptive combinée (ex. Alesse, Marvelon, Jasmine, Diane-35)
- ◯ Pilule contraceptive uniquement progestative ('mini-pilule', ex. Micronor)
- ◯ Je ne suis pas sûre du type de pilule contraceptive orale
- ◯ Progestatif en injection/piqûre (ex. Depo provera)
- ◯ Dispositifs transdermiques : timbres/patchs (ex. OrthoEvra, Climara), pastilles (Vivelledot)
- ◯ Anneau vaginal (NuvaRing)
- ◯ Stérilet/DIU contenant de la progestérone (Mirena, Kyleena)
- ◯ Implant hormonal (Implanon/Nexplanon)
- ◯ Progestatifs oraux (ex. diénogest [Visanne], acétate de médroxyprogestérone [Provera], dydrogestérone [Duphaston], acétate de noréthindrone[Norlutate])
- ◯ Agoniste de la GnRH en injection/piqûre (ex. acétate de leuprolide (leuproréline) [Prostap], goséréline [Zoladex])
- ◯ Acétate de noréthindrone (Norlutate) en addition à l'agoniste de la GnRH
- ◯ Antagoniste de la GnRH (elagolix [Orlissa])
- ◯ Danazol
- ◯ Hormonothérapie de remplacement ou traitement hormonal substitutif (ex. Premarin, Provera)
- ◯ Autre
- ◯ Je ne sais pas quel type d'hormone

Avez-vous utilisé le Danazol par voie vaginale ou orale?

- ◯ Vaginale
- ◯ Orale

Âge de début ________________________________

Utilisée dans les 3 derniers mois? ◯ Oui ◯ Non

Dans les deux questions ci-dessous, prière d'entrer la durée totale d'utilisation de cette hormone (années + mois). Si vous utilisation est de 1 an, indiquez : durée totale d'utilisation (années) : 1; durée totale d'utilisation (mois) : 0.

Durée totale d'utilisation (années) ________________________________ (années)

Durée totale d'utilisation (en mois)	◯ 0<br>◯ 1<br>◯ 2<br>◯ 3<br>◯ 4<br>◯ 5<br>◯ 6<br>◯ 7<br>◯ 8<br>◯ 9<br>◯ 10<br>◯ 11<br>(mois)
Si l'hormone utilisée était une injection, indiquez la date de la dernière injection.<br>__________________________________	
Quelle était la dose d'antagoniste de la GnRH (elagolix [Orlissa])?	◯ 150 mg 1 fois par jour<br>◯ 200 mg 2 fois par jour
HORMONE #2	
Nom de l'hormone (Par exemple : Alesse, Norlutate, etc.). Si vous ne vous souvenez pas du nom de l'hormone utilisée, veuillez inscrire .	__________________________________
Type d'hormone	◯ Pilule contraceptive combinée (ex. Alesse, Marvelon, Jasmine, Diane-35)<br>◯ Pilule contraceptive uniquement progestative ('mini-pilule', ex. Micronor)<br>◯ Je ne suis pas sûre du type de pilule contraceptive orale<br>◯ Progestatif en injection/piqûre (ex. Depo provera)<br>◯ Dispositifs transdermiques : timbres/patchs (ex. OrthoEvra, Climara), pastilles (Vivelledot)<br>◯ Anneau vaginal (NuvaRing)<br>◯ Stérilet/DIU contenant de la progestérone (Mirena, Kyleena)<br>◯ Implant hormonal (Implanon/Nexplanon)<br>◯ Progestatifs oraux (ex. diénogest [Visanne], acétate de médroxyprogestérone [Provera], dydrogestérone [Duphaston], acétate de noréthindrone[Norlutate])<br>◯ Agoniste de la GnRH en injection/piqûre (ex. acétate de leuprolide (leuproréline) [Prostap], goséréline [Zoladex])<br>◯ Acétate de noréthindrone (Norlutate) en addition à l'agoniste de la GnRH<br>◯ Antagoniste de la GnRH (elagolix [Orlissa])<br>◯ Danazol<br>◯ Hormonothérapie de remplacement ou traitement hormonal substitutif (ex. Premarin, Provera)<br>◯ Autre<br>◯ Je ne sais pas quel type d'hormone
Avez-vous utilisé le Danazol par voie vaginale ou orale?	◯ Vaginale<br>◯ Orale

Âge de début	__________________________________
Utilisée dans les 3 derniers mois?	◯ Oui ◯ Non

Dans les deux questions ci-dessous, prière d'entrer la durée totale d'utilisation de cette hormone (années + mois). Si vous utilisation est de 1 an, indiquez : durée totale d'utilisation (années) : 1; durée totale d'utilisation (mois) : 0.

Durée totale d'utilisation (années)	__________________________________<br>(années)
Durée totale d'utilisation (en mois)	◯ 0<br>◯ 1<br>◯ 2<br>◯ 3<br>◯ 4<br>◯ 5<br>◯ 6<br>◯ 7<br>◯ 8<br>◯ 9<br>◯ 10<br>◯ 11<br>(mois)

Si l'hormone utilisée était une injection, indiquez la date de la dernière injection.

____

Quelle était la dose d'antagoniste de la GnRH (elagolix [Orlissa])?	◯ 150 mg 1 fois par jour<br>◯ 200 mg 2 fois par jour

HORMONE #3

Nom de l'hormone (Par exemple : Alesse, Norlutate, etc.). Si vous ne vous souvenez pas du nom de l'hormone utilisée, veuillez inscrire .	__________________________________

Powered by REDCap

Type d'hormone	◯ Pilule contraceptive combinée (ex. Alesse, Marvelon, Jasmine, Diane-35)<br>◯ Pilule contraceptive uniquement progestative ('mini-pilule', ex. Micronor)<br>◯ Je ne suis pas sûre du type de pilule contraceptive orale<br>◯ Progestatif en injection/piqûre (ex. Depo provera)<br>◯ Dispositifs transdermiques : timbres/patchs (ex. OrthoEvra, Climara), pastilles (Vivelledot)<br>◯ Anneau vaginal (NuvaRing)<br>◯ Stérilet/DIU contenant de la progestérone (Mirena, Kyleena)<br>◯ Implant hormonal (Implanon/Nexplanon)<br>◯ Progestatifs oraux (ex. diénogest [Visanne], acétate de médroxyprogestérone [Provera], dydrogestérone [Duphaston], acétate de noréthindrone[Norlutate])<br>◯ Agoniste de la GnRH en injection/piqûre (ex. acétate de leuprolide (leuproréline) [Prostap], goséréline [Zoladex])<br>◯ Acétate de noréthindrone (Norlutate) en addition à l'agoniste de la GnRH<br>◯ Antagoniste de la GnRH (elagolix [Orlissa])<br>◯ Danazol<br>◯ Hormonothérapie de remplacement ou traitement hormonal substitutif (ex. Premarin, Provera)<br>◯ Autre<br>◯ Je ne sais pas quel type d'hormone
Avez-vous utilisé le Danazol par voie vaginale ou orale?	◯ Vaginale<br>◯ Orale
Âge de début	__________________________________
Utilisée dans les 3 derniers mois?	◯ Oui ◯ Non

Dans les deux questions ci-dessous, prière d'entrer la durée totale d'utilisation de cette hormone (années + mois). Si vous utilisation est de 1 an, indiquez : durée totale d'utilisation (années) : 1; durée totale d'utilisation (mois) : 0.

Durée totale d'utilisation (années)	__________________________________<br>(années)
Durée totale d'utilisation (en mois)	◯ 0<br>◯ 1<br>◯ 2<br>◯ 3<br>◯ 4<br>◯ 5<br>◯ 6<br>◯ 7<br>◯ 8<br>◯ 9<br>◯ 10<br>◯ 11<br>(mois)

Si l'hormone utilisée était une injection, indiquez la date de la dernière injection.

____

Quelle était la dose d'antagoniste de la GnRH (elagolix [Orlissa])?	◯ 150 mg 1 fois par jour<br>◯ 200 mg 2 fois par jour
HORMONE #4	
Nom de l'hormone (Par exemple : Alesse, Norlutate, etc.). Si vous ne vous souvenez pas du nom de l'hormone utilisée, veuillez inscrire .	__________________________________
Type d'hormone	◯ Pilule contraceptive combinée (ex. Alesse, Marvelon, Jasmine, Diane-35)<br>◯ Pilule contraceptive uniquement progestative ('mini-pilule', ex. Micronor)<br>◯ Je ne suis pas sûre du type de pilule contraceptive orale<br>◯ Progestatif en injection/piqûre (ex. Depo provera)<br>◯ Dispositifs transdermiques : timbres/patchs (ex. OrthoEvra, Climara), pastilles (Vivelledot)<br>◯ Anneau vaginal (NuvaRing)<br>◯ Stérilet/DIU contenant de la progestérone (Mirena, Kyleena)<br>◯ Implant hormonal (Implanon/Nexplanon)<br>◯ Progestatifs oraux (ex. diénogest [Visanne], acétate de médroxyprogestérone [Provera], dydrogestérone [Duphaston], acétate de noréthindrone[Norlutate])<br>◯ Agoniste de la GnRH en injection/piqûre (ex. acétate de leuprolide (leuproréline) [Prostap], goséréline [Zoladex])<br>◯ Acétate de noréthindrone (Norlutate) en addition à l'agoniste de la GnRH<br>◯ Antagoniste de la GnRH (elagolix [Orlissa])<br>◯ Danazol<br>◯ Hormonothérapie de remplacement ou traitement hormonal substitutif (ex. Premarin, Provera)<br>◯ Autre<br>◯ Je ne sais pas quel type d'hormone
Avez-vous utilisé le Danazol par voie vaginale ou orale?	◯ Vaginale<br>◯ Orale
Âge de début	__________________________________
Utilisée dans les 3 derniers mois?	◯ Oui ◯ Non

Dans les deux questions ci-dessous, prière d'entrer la durée totale d'utilisation de cette hormone (années + mois). Si vous utilisation est de 1 an, indiquez : durée totale d'utilisation (années) : 1; durée totale d'utilisation (mois) : 0.

Durée totale d'utilisation (années)	__________________________________<br>(années)

Durée totale d'utilisation (en mois)	◯ 0<br>◯ 1<br>◯ 2<br>◯ 3<br>◯ 4<br>◯ 5<br>◯ 6<br>◯ 7<br>◯ 8<br>◯ 9<br>◯ 10<br>◯ 11<br>(mois)
Si l'hormone utilisée était une injection, indiquez la date de la dernière injection.<br>______________________________	
Quelle était la dose d'antagoniste de la GnRH (elagolix [Orlissa])?	◯ 150 mg 1 fois par jour<br>◯ 200 mg 2 fois par jour
HORMONE #5	
Nom de l'hormone (Par exemple : Alesse, Norlutate, etc.). Si vous ne vous souvenez pas du nom de l'hormone utilisée, veuillez inscrire .	______________________________
Type d'hormone	◯ Pilule contraceptive combinée (ex. Alesse, Marvelon, Jasmine, Diane-35)<br>◯ Pilule contraceptive uniquement progestative ('mini-pilule', ex. Micronor)<br>◯ Je ne suis pas sûre du type de pilule contraceptive orale<br>◯ Progestatif en injection/piqûre (ex. Depo provera)<br>◯ Dispositifs transdermiques : timbres/patchs (ex. OrthoEvra, Climara), pastilles (Vivelledot)<br>◯ Anneau vaginal (NuvaRing)<br>◯ Stérilet/DIU contenant de la progestérone (Mirena, Kyleena)<br>◯ Implant hormonal (Implanon/Nexplanon)<br>◯ Progestatifs oraux (ex. diénogest [Visanne], acétate de médroxyprogestérone [Provera], dydrogestérone [Duphaston], acétate de noréthindrone[Norlutate])<br>◯ Agoniste de la GnRH en injection/piqûre (ex. acétate de leuprolide (leuproréline) [Prostap], goséréline [Zoladex])<br>◯ Acétate de noréthindrone (Norlutate) en addition à l'agoniste de la GnRH<br>◯ Antagoniste de la GnRH (elagolix [Orlissa])<br>◯ Danazol<br>◯ Hormonothérapie de remplacement ou traitement hormonal substitutif (ex. Premarin, Provera)<br>◯ Autre<br>◯ Je ne sais pas quel type d'hormone
Avez-vous utilisé le Danazol par voie vaginale ou orale?	◯ Vaginale<br>◯ Orale

Âge de début

____

Utilisée dans les 3 derniers mois? ◯ Oui ◯ Non

Dans les deux questions ci-dessous, prière d'entrer la durée totale d'utilisation de cette hormone (années + mois).
Si vous utilisation est de 1 an, indiquez : durée totale d'utilisation (années) : 1; durée totale d'utilisation (mois) : 0.

Durée totale d'utilisation (années)

____
(années)

Durée totale d'utilisation (en mois)

◯ 0
◯ 1
◯ 2
◯ 3
◯ 4
◯ 5
◯ 6
◯ 7
◯ 8
◯ 9
◯ 10
◯ 11
(mois)

Si l'hormone utilisée était une injection, indiquez la date de la dernière injection.

____

Quelle était la dose d'antagoniste de la GnRH (elagolix [Orlissa])?

◯ 150 mg 1 fois par jour
◯ 200 mg 2 fois par jour

HORMONE #6

Nom de l'hormone (Par exemple : Alesse, Norlutate, etc.). Si vous ne vous souvenez pas du nom de l'hormone utilisée, veuillez inscrire .

____

Type d'hormone	◯ Pilule contraceptive combinée (ex. Alesse, Marvelon, Jasmine, Diane-35)<br>◯ Pilule contraceptive uniquement progestative ('mini-pilule', ex. Micronor)<br>◯ Je ne suis pas sûre du type de pilule contraceptive orale<br>◯ Progestatif en injection/piqûre (ex. Depo provera)<br>◯ Dispositifs transdermiques : timbres/patchs (ex. OrthoEvra, Climara), pastilles (Vivelledot)<br>◯ Anneau vaginal (NuvaRing)<br>◯ Stérilet/DIU contenant de la progestérone (Mirena, Kyleena)<br>◯ Implant hormonal (Implanon/Nexplanon)<br>◯ Progestatifs oraux (ex. diénogest [Visanne], acétate de médroxyprogestérone [Provera], dydrogestérone [Duphaston], acétate de noréthindrone[Norlutate])<br>◯ Agoniste de la GnRH en injection/piqûre (ex. acétate de leuprolide (leuproréline) [Prostap], goséréline [Zoladex])<br>◯ Acétate de noréthindrone (Norlutate) en addition à l'agoniste de la GnRH<br>◯ Antagoniste de la GnRH (elagolix [Orlissa])<br>◯ Danazol<br>◯ Hormonothérapie de remplacement ou traitement hormonal substitutif (ex. Premarin, Provera)<br>◯ Autre<br>◯ Je ne sais pas quel type d'hormone
Avez-vous utilisé le Danazol par voie vaginale ou orale?	◯ Vaginale<br>◯ Orale
Âge de début	______________________
Utilisée dans les 3 derniers mois?	◯ Oui ◯ Non

Dans les deux questions ci-dessous, prière d'entrer la durée totale d'utilisation de cette hormone (années + mois). Si vous utilisation est de 1 an, indiquez : durée totale d'utilisation (années) : 1; durée totale d'utilisation (mois) : 0.

Durée totale d'utilisation (années)	______________________<br>(années)
Durée totale d'utilisation (en mois)	◯ 0<br>◯ 1<br>◯ 2<br>◯ 3<br>◯ 4<br>◯ 5<br>◯ 6<br>◯ 7<br>◯ 8<br>◯ 9<br>◯ 10<br>◯ 11<br>(mois)

Si l'hormone utilisée était une injection, indiquez la date de la dernière injection.

____

Quelle était la dose d'antagoniste de la GnRH (elagolix [Orlissa])?	◯ 150 mg 1 fois par jour<br>◯ 200 mg 2 fois par jour
HORMONE #7	
Nom de l'hormone (Par exemple : Alesse, Norlutate, etc.). Si vous ne vous souvenez pas du nom de l'hormone utilisée, veuillez inscrire .	______________________________
Type d'hormone	◯ Pilule contraceptive combinée (ex. Alesse, Marvelon, Jasmine, Diane-35)<br>◯ Pilule contraceptive uniquement progestative ('mini-pilule', ex. Micronor)<br>◯ Je ne suis pas sûre du type de pilule contraceptive orale<br>◯ Progestatif en injection/piqûre (ex. Depo provera)<br>◯ Dispositifs transdermiques : timbres/patchs (ex. OrthoEvra, Climara), pastilles (Vivelledot)<br>◯ Anneau vaginal (NuvaRing)<br>◯ Stérilet/DIU contenant de la progestérone (Mirena, Kyleena)<br>◯ Implant hormonal (Implanon/Nexplanon)<br>◯ Progestatifs oraux (ex. diénogest [Visanne], acétate de médroxyprogestérone [Provera], dydrogestérone [Duphaston], acétate de noréthindrone[Norlutate])<br>◯ Agoniste de la GnRH en injection/piqûre (ex. acétate de leuprolide (leuproréline) [Prostap], goséréline [Zoladex])<br>◯ Acétate de noréthindrone (Norlutate) en addition à l'agoniste de la GnRH<br>◯ Antagoniste de la GnRH (elagolix [Orlissa])<br>◯ Danazol<br>◯ Hormonothérapie de remplacement ou traitement hormonal substitutif (ex. Premarin, Provera)<br>◯ Autre<br>◯ Je ne sais pas quel type d'hormone
Avez-vous utilisé le Danazol par voie vaginale ou orale?	◯ Vaginale<br>◯ Orale
Âge de début	______________________________
Utilisée dans les 3 derniers mois?	◯ Oui ◯ Non

Dans les deux questions ci-dessous, prière d'entrer la durée totale d'utilisation de cette hormone (années + mois). Si vous utilisation est de 1 an, indiquez : durée totale d'utilisation (années) : 1; durée totale d'utilisation (mois) : 0.

Durée totale d'utilisation (années)	______________________________<br>(années)

Powered by REDCap

Durée totale d'utilisation (en mois)	◯ 0<br>◯ 1<br>◯ 2<br>◯ 3<br>◯ 4<br>◯ 5<br>◯ 6<br>◯ 7<br>◯ 8<br>◯ 9<br>◯ 10<br>◯ 11<br>(mois)

Si l'hormone utilisée était une injection, indiquez la date de la dernière injection.

____

Quelle était la dose d'antagoniste de la GnRH (elagolix [Orlissa])?	◯ 150 mg 1 fois par jour<br>◯ 200 mg 2 fois par jour
HORMONE #8	
Nom de l'hormone (Par exemple : Alesse, Norlutate, etc.). Si vous ne vous souvenez pas du nom de l'hormone utilisée, veuillez inscrire .	__________________________________
Type d'hormone	◯ Pilule contraceptive combinée (ex. Alesse, Marvelon, Jasmine, Diane-35)<br>◯ Pilule contraceptive uniquement progestative ('mini-pilule', ex. Micronor)<br>◯ Je ne suis pas sûre du type de pilule contraceptive orale<br>◯ Progestatif en injection/piqûre (ex. Depo provera)<br>◯ Dispositifs transdermiques : timbres/patchs (ex. OrthoEvra, Climara), pastilles (Vivelledot)<br>◯ Anneau vaginal (NuvaRing)<br>◯ Stérilet/DIU contenant de la progestérone (Mirena, Kyleena)<br>◯ Implant hormonal (Implanon/Nexplanon)<br>◯ Progestatifs oraux (ex. diénogest [Visanne], acétate de médroxyprogestérone [Provera], dydrogestérone [Duphaston], acétate de noréthindrone[Norlutate])<br>◯ Agoniste de la GnRH en injection/piqûre (ex. acétate de leuprolide (leuproréline) [Prostap], goséréline [Zoladex])<br>◯ Acétate de noréthindrone (Norlutate) en addition à l'agoniste de la GnRH<br>◯ Antagoniste de la GnRH (elagolix [Orlissa])<br>◯ Danazol<br>◯ Hormonothérapie de remplacement ou traitement hormonal substitutif (ex. Premarin, Provera)<br>◯ Autre<br>◯ Je ne sais pas quel type d'hormone
Avez-vous utilisé le Danazol par voie vaginale ou orale?	◯ Vaginale<br>◯ Orale

Âge de début

____

Utilisée dans les 3 derniers mois? ◯ Oui ◯ Non

Dans les deux questions ci-dessous, prière d'entrer la durée totale d'utilisation de cette hormone (années + mois).
Si vous utilisation est de 1 an, indiquez : durée totale d'utilisation (années) : 1; durée totale d'utilisation (mois) : 0.

Durée totale d'utilisation (années)

____
(années)

Durée totale d'utilisation (en mois)

◯ 0
◯ 1
◯ 2
◯ 3
◯ 4
◯ 5
◯ 6
◯ 7
◯ 8
◯ 9
◯ 10
◯ 11
(mois)

Si l'hormone utilisée était une injection, indiquez la date de la dernière injection.

____

Quelle était la dose d'antagoniste de la GnRH (elagolix [Orlissa])?

◯ 150 mg 1 fois par jour
◯ 200 mg 2 fois par jour

HORMONE #9

Nom de l'hormone (Par exemple : Alesse, Norlutate, etc.). Si vous ne vous souvenez pas du nom de l'hormone utilisée, veuillez inscrire .

____

Type d'hormone	◯ Pilule contraceptive combinée (ex. Alesse, Marvelon, Jasmine, Diane-35)<br>◯ Pilule contraceptive uniquement progestative ('mini-pilule', ex. Micronor)<br>◯ Je ne suis pas sûre du type de pilule contraceptive orale<br>◯ Progestatif en injection/piqûre (ex. Depo provera)<br>◯ Dispositifs transdermiques : timbres/patchs (ex. OrthoEvra, Climara), pastilles (Vivelledot)<br>◯ Anneau vaginal (NuvaRing)<br>◯ Stérilet/DIU contenant de la progestérone (Mirena, Kyleena)<br>◯ Implant hormonal (Implanon/Nexplanon)<br>◯ Progestatifs oraux (ex. diénogest [Visanne], acétate de médroxyprogestérone [Provera], dydrogestérone [Duphaston], acétate de noréthindrone[Norlutate])<br>◯ Agoniste de la GnRH en injection/piqûre (ex. acétate de leuprolide (leuproréline) [Prostap], goséréline [Zoladex])<br>◯ Acétate de noréthindrone (Norlutate) en addition à l'agoniste de la GnRH<br>◯ Antagoniste de la GnRH (elagolix [Orlissa])<br>◯ Danazol<br>◯ Hormonothérapie de remplacement ou traitement hormonal substitutif (ex. Premarin, Provera)<br>◯ Autre<br>◯ Je ne sais pas quel type d'hormone
Avez-vous utilisé le Danazol par voie vaginale ou orale?	◯ Vaginale<br>◯ Orale
Âge de début	______________________
Utilisée dans les 3 derniers mois?	◯ Oui ◯ Non

Dans les deux questions ci-dessous, prière d'entrer la durée totale d'utilisation de cette hormone (années + mois). Si vous utilisation est de 1 an, indiquez : durée totale d'utilisation (années) : 1; durée totale d'utilisation (mois) : 0.

Durée totale d'utilisation (années)	______________________<br>(années)
Durée totale d'utilisation (en mois)	◯ 0<br>◯ 1<br>◯ 2<br>◯ 3<br>◯ 4<br>◯ 5<br>◯ 6<br>◯ 7<br>◯ 8<br>◯ 9<br>◯ 10<br>◯ 11<br>(mois)

Si l'hormone utilisée était une injection, indiquez la date de la dernière injection.

____

Quelle était la dose d'antagoniste de la GnRH (elagolix [Orlissa])?	◯ 150 mg 1 fois par jour<br>◯ 200 mg 2 fois par jour
HORMONE #10	
Nom de l'hormone (Par exemple : Alesse, Norlutate, etc.). Si vous ne vous souvenez pas du nom de l'hormone utilisée, veuillez inscrire .	______________________
Type d'hormone	◯ Pilule contraceptive combinée (ex. Alesse, Marvelon, Jasmine, Diane-35)<br>◯ Pilule contraceptive uniquement progestative ('mini-pilule', ex. Micronor)<br>◯ Je ne suis pas sûre du type de pilule contraceptive orale<br>◯ Progestatif en injection/piqûre (ex. Depo provera)<br>◯ Dispositifs transdermiques : timbres/patchs (ex. OrthoEvra, Climara), pastilles (Vivelledot)<br>◯ Anneau vaginal (NuvaRing)<br>◯ Stérilet/DIU contenant de la progestérone (Mirena, Kyleena)<br>◯ Implant hormonal (Implanon/Nexplanon)<br>◯ Progestatifs oraux (ex. diénogest [Visanne], acétate de médroxyprogestérone [Provera], dydrogestérone [Duphaston], acétate de noréthindrone[Norlutate])<br>◯ Agoniste de la GnRH en injection/piqûre (ex. acétate de leuprolide (leuproréline) [Prostap], goséréline [Zoladex])<br>◯ Acétate de noréthindrone (Norlutate) en addition à l'agoniste de la GnRH<br>◯ Antagoniste de la GnRH (elagolix [Orlissa])<br>◯ Danazol<br>◯ Hormonothérapie de remplacement ou traitement hormonal substitutif (ex. Premarin, Provera)<br>◯ Autre<br>◯ Je ne sais pas quel type d'hormone
Avez-vous utilisé le Danazol par voie vaginale ou orale?	◯ Vaginale<br>◯ Orale
Âge de début	______________________
Utilisée dans les 3 derniers mois?	◯ Oui ◯ Non

Dans les deux questions ci-dessous, prière d'entrer la durée totale d'utilisation de cette hormone (années + mois). Si vous utilisation est de 1 an, indiquez : durée totale d'utilisation (années) : 1; durée totale d'utilisation (mois) : 0.

Durée totale d'utilisation (années)	______________________<br>(années)

Durée totale d'utilisation (en mois)	◯ 0<br>◯ 1<br>◯ 2<br>◯ 3<br>◯ 4<br>◯ 5<br>◯ 6<br>◯ 7<br>◯ 8<br>◯ 9<br>◯ 10<br>◯ 11<br>(mois)
Si l'hormone utilisée était une injection, indiquez la date de la dernière injection.<br><br>__________________________________	
Quelle était la dose d'antagoniste de la GnRH (elagolix [Orlissa])?	◯ 150 mg 1 fois par jour<br>◯ 200 mg 2 fois par jour
Quelles sont/étaient vos raisons de l'utilisation d'hormones ?	☐ Contraception / prévention de la grossesse<br>☐ Douleur pelvienne ou menstruations douloureuses<br>☐ Menstruations irrégulières<br>☐ Menstruations très abondantes<br>☐ Acné<br>☐ Syndrome des ovaires polykystiques (SOPK)<br>☐ Kyste ovarien<br>☐ Autre<br>((Cochez toutes les réponses applicables))
Avez-vous déjà utilisé une contraception d'urgence?	◯ Oui<br>◯ Non<br>◯ Je préfère ne pas répondre
Avez-vous utilisé une contraception d'urgence dans les 3 derniers mois?	◯ Oui ◯ Non
Les hormones ont-elles aider à réduire les douleurs ?	◯ Oui ◯ Non
Avez-vous déjà arrêté des hormones ou changé d'hormones parce qu'elles n'étaient pas assez efficaces pour contrôler les douleurs?	◯ Oui ◯ Non
Veuillez préciser les autres raisons pour l'utilisation d'hormones.	__________________________________

TRAITEMENT NON HORMONAL

Avez-vous utilisé un stérilet/DIU non hormonal (ex. stérilet de cuivre) dans les 3 derniers mois?	◯ Oui ◯ Non

Dans les deux questions ci-dessous, prière d'entrer la durée totale d'utilisation du stérilet/DIU non hormonal (années + mois).
Si vous utilisation est de 1 an, indiquez : durée totale d'utilisation (années) : 1; durée totale d'utilisation (mois) : 0.

Pendant combien de temps avez-vous utilisé un stérilet/DIU non hormonal?	______________________ (années)
Pendant combien de temps avez-vous utilisé un stérilet/DIU non hormonal?	◯ 1 ◯ 2 ◯ 3 ◯ 4<br>◯ 5 ◯ 6 ◯ 7 ◯ 8<br>◯ 9 ◯ 10 ◯ 11<br>(mois)

Grossesse et fertilité

Avez-vous déjà été enceinte (confirmé par un test de grossesse positif, y compris les fausses-couches, les grossesses extra-utérines ou les avortements)?

◯ Oui ◯ Non

Combien de fois avez-vous été enceinte (confirmé par un test de grossesse positif, y compris les fausses-couches, les grossesses extra-utérines ou les avortements)?

____

Veuillez compléter les questions suivantes pour chacune de vos grossesses, y compris les fausses-couches et les avortements. Si vous avez eu des jumeaux ou triplets, veuillez les compter comme UNE grossesse. Si vous êtes actuellement enceinte, ne pas inclure la grossesse actuelle . NOTE: Pour les questions suivantes, spontané signifie que les contractions ont débutés par eux-même, pas de travail signifie une césarienne sans contraction, travail provoqué signifie que vous avez eu des contractions après avoir reçu de la médication (excluant le décollement des membrane/ stripping).

Grossesse #1

Quel âge aviez-vous au début de la grossesse?

____
(années)

Quel traitement contre l'infertilité a été utilisé, le cas échéant, pour cette grossesse?

☐ Conception naturelle : aucun traitement contre l'infertilité
☐ Médicaments contre l'infertilité sous forme de comprimés pour stimuler l'ovulation (Clomid, clomifène)
☐ Insémination intra-utérine
☐ Fécondation in vitro (FIV/FIV avec micro-injection [ICSI])

Comment s'est terminé cette grossesse?

☐ Enfant unique né vivant
☐ Jumeaux ou triplés
☐ Fausse-couche
☐ Enfant mort-né
☐ Interruption de grossesse (avortement)
☐ Grossesse ectopique (dans la trompe ou à l'extérieur de l'utérus
☐ Grossesse môlaire
☐ Grossesse en cours
((Cochez toutes les réponses applicables))

Si la grossesse a donné lieu à une fausse-couche, une grossesse extra-utérine ou un avortement, quelle a été la prise en charge?

◯ Chirurgicale (curetage)
◯ Médicamenteuse (comprimés oraux et/ou vaginaux)
◯ Aucune prise en charge n'a été nécessaire

Si la grossesse s'est achevée par une naissance, l'accouchement s'est-il déroulé par voie vaginale ou par césarienne?

◯ Voie vaginale
◯ Césarienne

Y a-t-il eu une phase de travail avant l'accouchement et si oui, était-il provoqué ou spontané?

◯ Pas de travail
◯ Travail spontané
◯ Travail provoqué

Avez-vous connu l'une des complications suivantes liées à la grossesse ou l'allaitement?	☐ Diabète gestationnel<br>☐ Hypertension liée à la grossesse<br>☐ Pré-éclampsie/toxémie gravidique<br>☐ Mastite/infection du sein<br>☐ Syndrome HELLP<br>☐ Hyperémèse gravidique<br>☐ Naissance prématurée (avant 37 semaines)<br>☐ Autre
Autre complication #1	__________________________________
Autre complication #2	__________________________________
Pendant combien de temps avez-vous allaité ? (Indiquez le nombre de mois d'allaitement ou inscrivez '0' si vous n'avez pas allaité. Si vous avez allaité pendant moins d'un mois, inscrivez '1')	__________________________________<br>(mois)
Grossesse #2	
Quel âge aviez-vous au début de la grossesse?	__________________________________<br>(années)
Quel traitement contre l'infertilité a été utilisé, le cas échéant, pour cette grossesse?	☐ Conception naturelle : aucun traitement contre l'infertilité<br>☐ Médicaments contre l'infertilité sous forme de comprimés pour stimuler l'ovulation (Clomid, clomifène)<br>☐ Insémination intra-utérine<br>☐ Fécondation in vitro (FIV/FIV avec micro-injection [ICSI])
Comment s'est terminé cette grossesse?	☐ Enfant unique né vivant<br>☐ Jumeaux ou triplés<br>☐ Fausse-couche<br>☐ Enfant mort-né<br>☐ Interruption de grossesse (avortement)<br>☐ Grossesse ectopique (dans la trompe ou à l'extérieur de l'utérus)<br>☐ Grossesse môlaire<br>☐ Grossesse en cours<br>((Cochez toutes les réponses applicables))
Si la grossesse a donné lieu à une fausse-couche, une grossesse extra-utérine ou un avortement, quelle a été la prise en charge?	◯ Chirurgicale (curetage)<br>◯ Médicamenteuse (comprimés oraux et/ou vaginaux)<br>◯ Aucune prise en charge n'a été nécessaire
Si la grossesse s'est achevée par une naissance, l'accouchement s'est-il déroulé par voie vaginale ou par césarienne?	◯ Voie vaginale<br>◯ Césarienne
Y a-t-il eu une phase de travail avant l'accouchement et si oui, était-il provoqué ou spontané?	◯ Pas de travail<br>◯ Travail spontané<br>◯ Travail provoqué

Avez-vous connu l'une des complications suivantes liées à la grossesse ou l'allaitement?	☐ Diabète gestationnel<br>☐ Hypertension liée à la grossesse<br>☐ Pré-éclampsie/toxémie gravidique<br>☐ Mastite/infection du sein<br>☐ Syndrome HELLP<br>☐ Hyperémèse gravidique<br>☐ Naissance prématurée (avant 37 semaines)<br>☐ Autre
Autre complication #1	__________________________________
Autre complication #2	__________________________________
Pendant combien de temps avez-vous allaité ? (Indiquez le nombre de mois d'allaitement ou inscrivez '0' si vous n'avez pas allaité. Si vous avez allaité pendant moins d'un mois, inscrivez '1')	__________________________________<br>(mois)
Grossesse #3	
Quel âge aviez-vous au début de la grossesse?	__________________________________<br>(années)
Quel traitement contre l'infertilité a été utilisé, le cas échéant, pour cette grossesse?	☐ Conception naturelle : aucun traitement contre l'infertilité<br>☐ Médicaments contre l'infertilité sous forme de comprimés pour stimuler l'ovulation (Clomid, clomifène)<br>☐ Insémination intra-utérine<br>☐ Fécondation in vitro (FIV/FIV avec micro-injection [ICSI])
Comment s'est terminé cette grossesse?	☐ Enfant unique né vivant<br>☐ Jumeaux ou triplés<br>☐ Fausse-couche<br>☐ Enfant mort-né<br>☐ Interruption de grossesse (avortement)<br>☐ Grossesse ectopique (dans la trompe ou à l'extérieur de l'utérus<br>☐ Grossesse môlaire<br>☐ Grossesse en cours<br>((Cochez toutes les réponses applicables))
Si la grossesse a donné lieu à une fausse-couche, une grossesse extra-utérine ou un avortement, quelle a été la prise en charge?	◯ Chirurgicale (curetage)<br>◯ Médicamenteuse (comprimés oraux et/ou vaginaux)<br>◯ Aucune prise en charge n'a été nécessaire
Si la grossesse s'est achevée par une naissance, l'accouchement s'est-il déroulé par voie vaginale ou par césarienne?	◯ Voie vaginale<br>◯ Césarienne
Y a-t-il eu une phase de travail avant l'accouchement et si oui, était-il provoqué ou spontané?	◯ Pas de travail<br>◯ Travail spontané<br>◯ Travail provoqué

Avez-vous connu l'une des complications suivantes liées à la grossesse ou l'allaitement?

- ☐ Diabète gestationnel
- ☐ Hypertension liée à la grossesse
- ☐ Pré-éclampsie/toxémie gravidique
- ☐ Mastite/infection du sein
- ☐ Syndrome HELLP
- ☐ Hyperémèse gravidique
- ☐ Naissance prématurée (avant 37 semaines)
- ☐ Autre

Autre complication #1

____

Autre complication #2

____

Pendant combien de temps avez-vous allaité ? (Indiquez le nombre de mois d'allaitement ou inscrivez '0' si vous n'avez pas allaité. Si vous avez allaité pendant moins d'un mois, inscrivez '1')

____
(mois)

Grossesse #4

Quel âge aviez-vous au début de la grossesse?

____
(années)

Quel traitement contre l'infertilité a été utilisée, le cas échéant, pour cette grossesse?

- ☐ Conception naturelle : aucun traitement contre l'infertilité
- ☐ Médicaments contre l'infertilité sous forme de comprimés pour stimuler l'ovulation (Clomid, clomifène)
- ☐ Insémination intra-utérine
- ☐ Fécondation in vitro (FIV/FIV avec micro-injection [ICSI])

Comment s'est terminé cette grossesse?

- ☐ Enfant unique né vivant
- ☐ Jumeaux ou triplés
- ☐ Fausse-couche
- ☐ Enfant mort-né
- ☐ Interruption de grossesse (avortement)
- ☐ Grossesse ectopique (dans la trompe ou à l'extérieur de l'utérus
- ☐ Grossesse môlaire
- ☐ Grossesse en cours

((Cochez toutes les réponses applicables))

Si la grossesse a donné lieu à une fausse-couche, une grossesse extra-utérine ou un avortement, quelle a été la prise en charge?

- ◯ Chirurgicale (curetage)
- ◯ Médicamenteuse (comprimés oraux et/ou vaginaux)
- ◯ Aucune prise en charge n'a été nécessaire

Si la grossesse s'est achevée par une naissance, l'accouchement s'est-il déroulé par voie vaginale ou par césarienne?

- ◯ Voie vaginale
- ◯ Césarienne

Y a-t-il eu une phase de travail avant l'accouchement et si oui, était-il provoqué ou spontané?

- ◯ Pas de travail
- ◯ Travail spontané
- ◯ Travail provoqué

Avez-vous connu l'une des complications suivantes liées à la grossesse ou l'allaitement?	☐ Diabète gestationnel<br>☐ Hypertension liée à la grossesse<br>☐ Pré-éclampsie/toxémie gravidique<br>☐ Mastite/infection du sein<br>☐ Syndrome HELLP<br>☐ Hyperémèse gravidique<br>☐ Naissance prématurée (avant 37 semaines)<br>☐ Autre
Autre complication #1	__________________________________
Autre complication #2	__________________________________
Pendant combien de temps avez-vous allaité ? (Indiquez le nombre de mois d'allaitement ou inscrivez '0' si vous n'avez pas allaité. Si vous avez allaité pendant moins d'un mois, inscrivez '1')	__________________________________<br>(mois)
Grossesse #5	
Quel âge aviez-vous au début de la grossesse?	__________________________________<br>(années)
Quel traitement contre l'infertilité a été utilisé, le cas échéant, pour cette grossesse?	☐ Conception naturelle : aucun traitement contre l'infertilité<br>☐ Médicaments contre l'infertilité sous forme de comprimés pour stimuler l'ovulation (Clomid, clomifène)<br>☐ Insémination intra-utérine<br>☐ Fécondation in vitro (FIV/FIV avec micro-injection [ICSI])
Comment s'est terminé cette grossesse?	☐ Enfant unique né vivant<br>☐ Jumeaux ou triplés<br>☐ Fausse-couche<br>☐ Enfant mort-né<br>☐ Interruption de grossesse (avortement)<br>☐ Grossesse ectopique (dans la trompe ou à l'extérieur de l'utérus<br>☐ Grossesse môlaire<br>☐ Grossesse en cours<br>((Cochez toutes les réponses applicables))
Si la grossesse a donné lieu à une fausse-couche, une grossesse extra-utérine ou un avortement, quelle a été la prise en charge?	◯ Chirurgicale (curetage)<br>◯ Médicamenteuse (comprimés oraux et/ou vaginaux)<br>◯ Aucune prise en charge n'a été nécessaire
Si la grossesse s'est achevée par une naissance, l'accouchement s'est-il déroulé par voie vaginale ou par césarienne?	◯ Voie vaginale<br>◯ Césarienne
Y a-t-il eu une phase de travail avant l'accouchement et si oui, était-il provoqué ou spontané?	◯ Pas de travail<br>◯ Travail spontané<br>◯ Travail provoqué

Avez-vous connu l'une des complications suivantes liées à la grossesse ou l'allaitement?	☐ Diabète gestationnel<br>☐ Hypertension liée à la grossesse<br>☐ Pré-éclampsie/toxémie gravidique<br>☐ Mastite/infection du sein<br>☐ Syndrome HELLP<br>☐ Hyperémèse gravidique<br>☐ Naissance prématurée (avant 37 semaines)<br>☐ Autre
Autre complication #1	________________________________
Autre complication #2	________________________________
Pendant combien de temps avez-vous allaité ? (Indiquez le nombre de mois d'allaitement ou inscrivez '0' si vous n'avez pas allaité. Si vous avez allaité pendant moins d'un mois, inscrivez '1')	________________________________<br>(mois)
Grossesse #6	
Quel âge aviez-vous au début de la grossesse?	________________________________<br>(années)
Quel traitement contre l'infertilité a été utilisé, le cas échéant, pour cette grossesse	☐ Conception naturelle : aucun traitement contre l'infertilité<br>☐ Médicaments contre l'infertilité sous forme de comprimés pour stimuler l'ovulation (Clomid, clomifène)<br>☐ Insémination intra-utérine<br>☐ Fécondation in vitro (FIV/FIV avec micro-injection [ICSI])
Comment s'est terminé cette grossesse?	☐ Enfant unique né vivant<br>☐ Jumeaux ou triplés<br>☐ Fausse-couche<br>☐ Enfant mort-né<br>☐ Interruption de grossesse (avortement)<br>☐ Grossesse ectopique (dans la trompe ou à l'extérieur de l'utérus<br>☐ Grossesse môlaire<br>☐ Grossesse en cours<br>((Cochez toutes les réponses applicables))
Si la grossesse a donné lieu à une fausse-couche, une grossesse extra-utérine ou un avortement, quelle a été la prise en charge?	◯ Chirurgicale (curetage)<br>◯ Médicamenteuse (comprimés oraux et/ou vaginaux)<br>◯ Aucune prise en charge n'a été nécessaire
Si la grossesse s'est achevée par une naissance, l'accouchement s'est-il déroulé par voie vaginale ou par césarienne?	◯ Voie vaginale<br>◯ Césarienne
Y a-t-il eu une phase de travail avant l'accouchement et si oui, était-il provoqué ou spontané?	◯ Pas de travail<br>◯ Travail spontané<br>◯ Travail provoqué

Avez-vous connu l'une des complications suivantes liées à la grossesse ou l'allaitement?	☐ Diabète gestationnel<br>☐ Hypertension liée à la grossesse<br>☐ Pré-éclampsie/toxémie gravidique<br>☐ Mastite/infection du sein<br>☐ Syndrome HELLP<br>☐ Hyperémèse gravidique<br>☐ Naissance prématurée (avant 37 semaines)<br>☐ Autre
Autre complication #1	__________________________
Autre complication #2	__________________________
Pendant combien de temps avez-vous allaité ? (Indiquez le nombre de mois d'allaitement ou inscrivez '0' si vous n'avez pas allaité. Si vous avez allaité pendant moins d'un mois, inscrivez '1')	__________________________<br>(mois)
Grossesse #7	
Quel âge aviez-vous au début de la grossesse?	__________________________<br>(années)
Quel traitement contre l'infertilité a été utilisé, le cas échéant, pour cette grossesse?	☐ Conception naturelle : aucun traitement contre l'infertilité<br>☐ Médicaments contre l'infertilité sous forme de comprimés pour stimuler l'ovulation (Clomid, clomifène)<br>☐ Insémination intra-utérine<br>☐ Fécondation in vitro (FIV/FIV avec micro-injection [ICSI])
Comment s'est terminé cette grossesse?	☐ Enfant unique né vivant<br>☐ Jumeaux ou triplés<br>☐ Fausse-couche<br>☐ Enfant mort-né<br>☐ Interruption de grossesse (avortement)<br>☐ Grossesse ectopique (dans la trompe ou à l'extérieur de l'utérus<br>☐ Grossesse môlaire<br>☐ Grossesse en cours<br>((Cochez toutes les réponses applicables))
Si la grossesse a donné lieu à une fausse-couche, une grossesse extra-utérine ou un avortement, quelle a été la prise en charge?	◯ Chirurgicale (curetage)<br>◯ Médicamenteuse (comprimés oraux et/ou vaginaux)<br>◯ Aucune prise en charge n'a été nécessaire
Si la grossesse s'est achevée par une naissance, l'accouchement s'est-il déroulé par voie vaginale ou par césarienne?	◯ Voie vaginale<br>◯ Césarienne
Y a-t-il eu une phase de travail avant l'accouchement et si oui, était-il provoqué ou spontané?	◯ Pas de travail<br>◯ Travail spontané<br>◯ Travail provoqué

Question	Réponse
Avez-vous connu l'une des complications suivantes liées à la grossesse ou l'allaitement?	☐ Diabète gestationnel<br>☐ Hypertension liée à la grossesse<br>☐ Pré-éclampsie/toxémie gravidique<br>☐ Mastite/infection du sein<br>☐ Syndrome HELLP<br>☐ Hyperémèse gravidique<br>☐ Naissance prématurée (avant 37 semaines)<br>☐ Autre
Autre complication #1	__________________
Autre complication #2	__________________
Pendant combien de temps avez-vous allaité ? (Indiquez le nombre de mois d'allaitement ou inscrivez '0' si vous n'avez pas allaité. Si vous avez allaité pendant moins d'un mois, inscrivez '1')	__________________<br>(mois)
Grossesse #8	
Quel âge aviez-vous au début de la grossesse?	__________________<br>(années)
Quel traitement contre l'infertilité a été utilisé, le cas échéant, pour cette grossesse?	☐ Conception naturelle : aucun traitement contre l'infertilité<br>☐ Médicaments contre l'infertilité sous forme de comprimés pour stimuler l'ovulation (Clomid, clomifène)<br>☐ Insémination intra-utérine<br>☐ Fécondation in vitro (FIV/FIV avec micro-injection [ICSI])
Comment s'est terminé cette grossesse?	☐ Enfant unique né vivant<br>☐ Jumeaux ou triplés<br>☐ Fausse-couche<br>☐ Enfant mort-né<br>☐ Interruption de grossesse (avortement)<br>☐ Grossesse ectopique (dans la trompe ou à l'extérieur de l'utérus<br>☐ Grossesse môlaire<br>☐ Grossesse en cours<br>((Cochez toutes les réponses applicables)")
Si la grossesse a donné lieu à une fausse-couche, une grossesse extra-utérine ou un avortement, quelle a été la prise en charge?	◯ Chirurgicale (curetage)<br>◯ Médicamenteuse (comprimés oraux et/ou vaginaux)<br>◯ Aucune prise en charge n'a été nécessaire
Si la grossesse s'est achevée par une naissance, l'accouchement s'est-il déroulé par voie vaginale ou par césarienne?	◯ Voie vaginale<br>◯ Césarienne
Y a-t-il eu une phase de travail avant l'accouchement et si oui, était-il provoqué ou spontané?	◯ Pas de travail<br>◯ Travail spontané<br>◯ Travail provoqué

Avez-vous connu l'une des complications suivantes liées à la grossesse ou l'allaitement?	☐ Diabète gestationnel<br>☐ Hypertension liée à la grossesse<br>☐ Pré-éclampsie/toxémie gravidique<br>☐ Mastite/infection du sein<br>☐ Syndrome HELLP<br>☐ Hyperémèse gravidique<br>☐ Naissance prématurée (avant 37 semaines)<br>☐ Autre
Autre complication #1	__________________________________
Autre complication #2	__________________________________
Pendant combien de temps avez-vous allaité ? (Indiquez le nombre de mois d'allaitement ou inscrivez '0' si vous n'avez pas allaité. Si vous avez allaité pendant moins d'un mois, inscrivez '1')	__________________________________<br>(mois)

Fertilité

Essayez-vous présentement d'être enceinte?	◯ Oui ◯ Non
Avez-vous déjà essayé d'être enceinte pendant plus de 6 mois d'affilée sans y parvenir?	◯ Oui ◯ Non
Quelle a été la durée de votre tentative la plus longue, qu'elle se soit terminé ou non par une grossesse?	______________ (mois)
Est-ce que vous ou votre partenaire avez déjà passé des tests/examens pour déterminer pourquoi vous ne parveniez pas à être enceinte?	◯ Oui ◯ Non
Quels ont été les résultats de ces tests?	☐ Endométriose<br>☐ Syndrome des ovaires polykystiques (SOPK)<br>☐ Maladie inflammatoire pelvienne<br>☐ Fibromes utérins<br>☐ Trompes bouchées<br>☐ Ovulation absente/irrégulière<br>☐ Quantité/qualité insuffisante du sperme<br>☐ Adhérences<br>☐ Autre<br>☐ Aucune cause n'a été identifiée<br>☐ Je ne m'en souviens pas<br>((Cochez toutes les réponses applicables))
Autre résultat de ces tests	______________
Avez-vous déjà consulté dans une clinique pour recevoir un traitement contre l'infertilité?	◯ Oui ◯ Non
Veuillez nous indiquer les traitements contre l'infertilité que vous avez utilisés dans les questions suivantes.	
Rapports sexuels programmés dans le temps spécifiquement pour concevoir	◯ Jamais utilisé ◯ Utilisé dans les trois derniers mois ◯ Utilisé, mais pas dans les trois derniers mois
Médicaments contre l'infertilité sous forme de comprimés pour stimuler l'ovulation (clomid, clomifène ou tout autre médicament sous forme de comprimé)	◯ Jamais utilisé ◯ Utilisé dans les trois derniers mois ◯ Utilisé, mais pas dans les trois derniers mois
Combien de cycles avez-vous eu pour ce traitement?	______________
Médicaments contre l'infertilité en injection (gonadotrophines, HCG ou tout autre médicament injecté)	◯ Jamais utilisé ◯ Utilisé dans les trois derniers mois ◯ Utilisé, mais pas dans les trois derniers mois
Combien de cycles avez-vous eu pour ce traitement?	______________
Progestérone (injection vaginale ou intramusculaire)?	◯ Jamais utilisé ◯ Utilisé dans les trois derniers mois ◯ Utilisé, mais pas dans les trois derniers mois

Combien de cycles avez-vous eu pour ce traitement?	__________________________
Insémination avec le sperme de votre partenaire	◯ Jamais utilisé ◯ Utilisé dans les trois derniers mois ◯ Utilisé, mais pas dans les trois derniers mois
Combien de cycles avez-vous eu pour ce traitement?	__________________________
Insémination intra-utérine avec le sperme d'un donneur	◯ Jamais utilisé ◯ Utilisé dans les trois derniers mois ◯ Utilisé, mais pas dans les trois derniers mois
Combien de cycles avez-vous eu pour ce traitement?	__________________________
Fécondation in vitro (FIV)	◯ Jamais utilisé ◯ Utilisé dans les trois derniers mois ◯ Utilisé, mais pas dans les trois derniers mois
Combien de cycles avez-vous eu pour ce traitement?	__________________________
Fécondation in vitro avec micro-injection (ICSI)	◯ Jamais utilisé ◯ Utilisé dans les trois derniers mois ◯ Utilisé, mais pas dans les trois derniers mois
Combien de cycles avez-vous eu pour ce traitement?	__________________________
Fécondation in vitro avec don d'ovules	◯ Jamais utilisé ◯ Utilisé dans les trois derniers mois ◯ Utilisé, mais pas dans les trois derniers mois
Combien de cycles avez-vous eu pour ce traitement?	__________________________
Après quelle étape votre (vos) cycle(s) de FIV s'est (se sont)-il(s) terminé(s)?	☐ Stimulation ovarienne (pas d'ovocytes prêts à ponctionner)<br>☐ Ponction des ovocytes (pas de transfert des embryons)<br>☐ Transfert d'embryon (pas de test de grossesse positif)<br>☐ Grossesse chimiquement confirmée (test de grossesse positif mais pas de battements de cœur à l'échographie)<br>☐ Grossesse cliniquement confirmée (battements de cœur détectés, mais perte de grossesse avant la fin de la semaine 12)<br>☐ Perte de grossesse ou enfant mort-né après la semaine 12<br>☐ Enfant né vivant<br>(Cochez toutes les réponses applicables)
Si vous voulez ajouter des précisions à vos réponses précédentes, veuillez utiliser l'espace suivant :	________________________________

Douleur

Répondez aux questions en fonction de votre état actuel, sauf si mentionné autrement.

Douleur et menstruation

Avez-vous déjà eu des crampes menstruelles douloureuses?	◯ Oui ◯ Non
EN GÉNÉRAL, quelle a été l'intensité de la douleur de vos crampes menstruelles sur une échelle de 0 à 10 où 0 = aucune douleur et 10 = la pire douleur imaginable?	◯ 0<br>◯ 1<br>◯ 2<br>◯ 3<br>◯ 4<br>◯ 5<br>◯ 6<br>◯ 7<br>◯ 8<br>◯ 9<br>◯ 10<br>◯ Aucun saignement
Dans les 3 DERNIERS MOIS, quelle a été l'intensité de la douleur de vos crampes menstruelles?	◯ 0<br>◯ 1<br>◯ 2<br>◯ 3<br>◯ 4<br>◯ 5<br>◯ 6<br>◯ 7<br>◯ 8<br>◯ 9<br>◯ 10<br>◯ Aucun saignement
Dans les 3 DERNIERS MOIS, combien de jours, au total, avez-vous eu vos des saignements vaginaux?	◯ 0<br>◯ 1-10<br>◯ 10-20<br>◯ 20-40<br>◯ 40-60<br>◯ 60-80<br>◯ >80

Powered by REDCap

Douleur liée au transit digestif

Dans les 3 DERNIERS MOIS, quelle a été l'intensité de la douleur lorsque vous êtes allées à la selle?
Si vous n'avez pas eu de douleur, veuillez indiquer 0.

◯ 0
◯ 1
◯ 2
◯ 3
◯ 4
◯ 5
◯ 6
◯ 7
◯ 8
◯ 9
◯ 10

Avez-vous de la douleur abdominale en moyenne au moins 1 journée/semaine durant les 3 derniers mois (débutant au moins 6 mois avant ce questionnaire) qui est associée aux éléments suivants :

	Oui	Non
Changement dans la fréquence des selles?	◯	◯
Changement dans la forme des selles?	◯	◯
Défécation?	◯	◯

Douleur liée à la vessie

Avez-vous une sensation désagréable (ex. douleur, pression, inconfort) qui semble liée à la vessie (qui dure depuis au moins 6 semaines)?	◯ Oui ◯ Non
Est-ce que cette douleur EMPIRE quand la vessie se remplit?	◯ Oui ◯ Non
Avez-vous une forte envie (pressante) d'uriner en raison de la douleur?	◯ Oui ◯ Non
Urinez-vous plus souvent que dans le passé?	◯ Oui ◯ Non

Autres douleurs

Dans les 3 DERNIERS MOIS, avez-vous eu des douleurs pelviennes (sans tenir compte des crampes menstruelles, des douleurs liées aux rapports sexuels et des douleurs liées au passage des selles)?	◯ Oui ◯ Non
Dans les 3 DERNIERS MOIS, quelle a été l'intensité de cette autre douleur pelvienne?	◯ 0 ◯ 1 ◯ 2 ◯ 3<br>◯ 4 ◯ 5 ◯ 6 ◯ 7<br>◯ 8 ◯ 9 ◯ 10
Dans les 3 DERNIERS MOIS, avez-vous eu des douleurs dans le bas du dos?	◯ Oui ◯ Non
Dans les 3 DERNIERS MOIS, quelle a été l'intensité de cette douleur au bas du dos?	◯ 0 ◯ 1 ◯ 2 ◯ 3<br>◯ 4 ◯ 5 ◯ 6 ◯ 7<br>◯ 8 ◯ 9 ◯ 10

-Douleur lors de rapports sexuels

Si vous n'avez jamais eu de rapports sexuels, veuillez l'indiquer ici.

◯ Je n'ai jamais eu de rapports sexuels.

Les questions suivantes concernent les douleurs pelviennes pendant ou après les rapports sexuels. Nous vous rappelons que toutes les informations fournies seront traitées de façon totalement confidentielle. Si toutefois vous ne souhaitez pas répondre à ces questions, veuillez l'indiquer ici.

◯ Je ne désire pas répondre à ces questions.

Avez-vous déjà eu des douleurs pelviennes pendant les rapports sexuels ou dans les 24 heures suivant un rapport sexuel vaginal/une pénétration?

◯ Oui ◯ Non

À quel âge ces douleurs ont-elles débuté ?

____

Si vous êtes sexuellement actives ou l'avez été dans le passé, quelle a été l'intensité de la douleur lors de la pénétration sur une échelle de 0 à 10 où 0 = aucune douleur et 10 = la pire douleur imaginable?

	0	1	2	3	4	5	6	7	8	9	10
En profondeur dans le vagin	◯	◯	◯	◯	◯	◯	◯	◯	◯	◯	◯
À l'entrée du vagin	◯	◯	◯	◯	◯	◯	◯	◯	◯	◯	◯

Quand a eu lieu votre dernier rapport sexuel vaginal ?

◯ Au cours du dernier mois
◯ Il y a 1-3 mois ◯ Il y a 4-12 mois
◯ Il y a plus de 12 mois

Dans ce cas, avez-vous évité les rapports sexuels en raison des douleurs pelviennes ?

◯ Oui ◯ Non

La dernière fois que vous avez eu un rapport sexuel, avez-vous eu des douleurs pelviennes pendant ou dans les 24 heures suivant le rapport sexuel ?

◯ Non ◯ Oui, pendant le rapport/la pénétration ◯ Oui, dans les 24 heures suivant le rapport/la pénétration
◯ Oui, pendant le rapport/la pénétration et aussi dans les 24 heures suivantes

La dernière fois que vous avez eu un rapport sexuel vaginal/pénétration, où la douleur s'est-elle manifestée ?

☐ À l'entrée du vagin
☐ En profondeur dans le vagin
☐ Dans l'abdomen/la région du bassin
☐ Autre localisation
(Cochez toutes les réponses applicables)

Veuillez préciser la localisation de la douleur :

____

Veuillez indiquer l'intensité de vos douleurs pelviennes au plus fort de la douleur la dernière fois que vous avez eu un rapport sexuel vaginal/pénétration sur une échelle de 0 à 10 où 0 = aucune douleur et 10 = la pire douleur imaginable.

◯ 0
◯ 1
◯ 2
◯ 3
◯ 4
◯ 5
◯ 6
◯ 7
◯ 8
◯ 9
◯ 10

Veuillez indiquer l'intensité de vos douleurs pelviennes au plus fort de la douleur dans les 24 heures ayant suivi votre dernier rapport sexuel vaginal/pénétration sur une échelle de 0 à 10 où 0 = aucune douleur et 10 = la pire douleur imaginable.

◯ 0
◯ 1
◯ 2
◯ 3
◯ 4
◯ 5
◯ 6
◯ 7
◯ 8
◯ 9
◯ 10

Lorsque vous avez eu des rapports sexuels vaginaux/pénétration dans les 12 derniers mois, à quelle fréquence avez-vous eu des douleurs pelviennes pendant ou dans les 24 heures suivant le rapport ?

◯ Jamais
◯ Occasionnellement (moins d'une fois sur quatre)
◯ Souvent (entre une fois sur quatre et une fois sur deux)
◯ Très souvent (plus d'une fois sur deux)
◯ Systématiquement (à chaque fois)

Dans les 12 derniers mois, y a-t-il eu une période du mois où les rapports sexuels vaginaux/la pénétration étaient plus douloureux que le reste du temps?

	Les rapports sexuels étaient PLUS douloureux à ce moment que le reste du temps.	Les rapports sexuels n'étaient PAS plus douloureux à ce moment que le reste du temps.	Non applicable, aucun rapport sexuel tenté durant cette période
Pendant les règles	◯	◯	◯
Quelques jours avant les règles	◯	◯	◯
Quelques jours après les règles	◯	◯	◯
En milieu de cycle (autour de l'ovulation)	◯	◯	◯

Dans les 12 DERNIERS MOIS, avez-vous déjà dû interrompre un rapport sexuel en raison de douleurs pelviennes? ◯ Oui ◯ Non

Dans les 12 DERNIERS MOIS, avez-vous déjà évité un rapport sexuel en raison de douleurs pelviennes?	◯ Oui ◯ Non
Quel âge aviez-vous lorsque les douleurs pelviennes liées aux rapports sexuels étaient les plus fortes?	____________________________________<br>(ans)
Veuillez indiquer l'intensité des douleurs pelviennes liées aux rapports sexuels lorsqu'elles étaient les plus fortes sur une échelle de 0 à 10 où 0 = aucune douleur et 10 = la pire douleur imaginable.	◯ 0<br>◯ 1<br>◯ 2<br>◯ 3<br>◯ 4<br>◯ 5<br>◯ 6<br>◯ 7<br>◯ 8<br>◯ 9<br>◯ 10
À quelle fréquence vous êtes vous senti en détresse à propos de votre vie sexuelle dans les 30 derniers jours incluant aujourd'hui?	◯ Jamais<br>◯ Rarement<br>◯ Parfois<br>◯ Souvent<br>◯ Toujours<br>◯ Ne s'applique pas

Douleur pelvienne/abdominale

Les questions de cette partie concernent les douleurs pelviennes/du bas ventre en général. Par 'douleurs pelviennes', nous entendons tout type de douleur (crampe, douleur lancinante, douleur vive, etc.) dans la région du bas ventre indiquée en gris sur cette image.

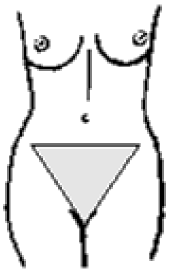

Avez-vous déjà eu des douleurs pelviennes de façon générale?
Ne prenez pas en compte : les douleurs liées aux opérations chirurgicales, à la grossesse, à l'accouchement, aux blessures sportives ou autres, aux intoxications alimentaires ou à la gastro-entérite.

◯ Oui ◯ Non

Quand avez-vous eu des douleurs pelviennes pour la dernière fois?

◯ Au cours du dernier mois
◯ Il y a 1-3 mois
◯ Il y a 4-6 mois
◯ Il y a 7-12 mois
◯ Il y a plus de 12 mois

Au total, pendant combien de temps environ avez-vous eu des douleurs pelviennes dans les 3 DERNIERS MOIS?

◯ Moins d'un jour par mois
◯ Un jour par mois
◯ Deux ou trois jours par mois
◯ Un jour par semaine
◯ Plus d'un jour par semaine
◯ Tous les jours

Avez-vous pris des médicaments pour aider à atténuer les douleurs pelviennes dans les 3 derniers mois?

☐ Non
☐ Oui, des anti-douleurs prescrits par un médecin
☐ Oui, des anti-douleurs en vente libre, sans ordonnance (p. ex. aspirine, ibuprofène, paracétamol/acétaminophène, naproxène)
☐ Oui, des hormones, mais elles n'ont pas atténué la douleur
☐ Oui, des hormones, et la douleur a été au moins en partie atténuée
(Cochez toutes les réponses applicables)

Veuillez indiquer l'intensité de vos douleurs pelviennes au plus fort de la douleur dans les 3 derniers mois sur une échelle de 0 à 10 où 0 = aucune douleur et 10 = la pire douleur imaginable.

◯ 1
◯ 2
◯ 3
◯ 4
◯ 5
◯ 6
◯ 7
◯ 8
◯ 9
◯ 10

Les questions suivantes concernent votre transit intestinal/vos selles lorsque vous avez eu des douleurs pelviennes dans les 3 DERNIERS MOIS , à quelle fréquence...

	Jamais/Rarement	Parfois	Souvent	La plupart du temps	Toujours
La douleur s'est-elle atténuée ou arrêtée après le passage à la selle?	◯	◯	◯	◯	◯
La douleur a-t-elle empiré après le passage à la selle?	◯	◯	◯	◯	◯
Alliez-vous plus fréquemment à la selle quand la douleur débutait?	◯	◯	◯	◯	◯
Avez-vous dû aller moins fréquemment à la selle quand la douleur débutait?	◯	◯	◯	◯	◯
Vos selles étaient-elles plus molles quand la douleur débutait?	◯	◯	◯	◯	◯
Vos selles étaient-elles plus dures quand la douleur débutait?	◯	◯	◯	◯	◯

Les questions suivantes concernent la période de votre vie où les douleurs pelviennes/du bas ventre étaient les plus fortes. Veuillez ne pas prendre en compte : les douleurs liées aux règles ou aux rapports sexuels, à la grossesse ou l'accouchement, aux opérations chirurgicales, aux blessures sportives ou autres, aux intoxications alimentaires ou à la gastro-entérite."

Quel âge aviez-vous lorsque les douleurs pelviennes/du bas ventre étaient les plus fortes ?

____
(ans)

Veuillez indiquer l'intensité de vos douleurs pelviennes/du bas ventre lorsqu'elles étaient les plus fortes sur une échelle de 0 à 10 où 0 = aucune douleur et 10 = la pire douleur imaginable.

◯ 0
◯ 1
◯ 2
◯ 3
◯ 4
◯ 5
◯ 6
◯ 7
◯ 8
◯ 9
◯ 10

Pendant la période de votre vie où les douleurs pelviennes/du bas ventre étaient les plus fortes, preniez-vous des médicaments pour aider à atténuer la douleur?

☐ Non
☐ Oui, des anti-douleurs prescrits par un médecin
☐ Oui, des anti-douleurs en vente libre, sans ordonnance (p. ex. aspirine, ibuprofène, paracétamol/acétaminophène, naproxène)
☐ Oui, des hormones, mais elles n'ont pas atténué la douleur
☐ Oui, des hormones, et la douleur a été au moins en partie atténuée
((Cochez toutes les réponses applicables))

Veuillez indiquer si vous avez connu les autres types de douleur suivants dans les 12 derniers mois :

	Non	Oui, au cours du dernier mois	Oui, il y a plus d'un mois
Douleur dans le bas du dos	○	○	○
Douleur musculaire/articulaire non liée à une infection virale ou une blessure (sportive)	○	○	○
Douleur lors de l'ovulation (en milieu de cycle)	○	○	○
Douleur dans les jambes	○	○	○
Douleur lorsque vous urinez	○	○	○
Douleur lorsque vous allez à la selle	○	○	○

La liste de mots ci-dessous décrit certaines caractéristiques de la douleur et des symptômes. Pour chaque description, veuillez indiquer ce qui décrit le mieux l'intensité générale de vos douleurs pelviennes/abdominales, DANS LES 3 DERNIERS MOIS, sur une échelle de 0 à 10 où 0 = aucune douleur et 10 = la pire douleur imaginable. Sélectionner 0 si le mot ne décrit pas votre douleur ou vos symptômes.

	0	1	2	3	4	5	6	7	8	9	10
Douleur pulsatile	○	○	○	○	○	○	○	○	○	○	○
Douleur lancinante	○	○	○	○	○	○	○	○	○	○	○
Douleur vive	○	○	○	○	○	○	○	○	○	○	○
Douleur aiguë	○	○	○	○	○	○	○	○	○	○	○
Crampe	○	○	○	○	○	○	○	○	○	○	○
Douleur tenaillante	○	○	○	○	○	○	○	○	○	○	○
Chaleur-brûlure	○	○	○	○	○	○	○	○	○	○	○
Douleur sourde	○	○	○	○	○	○	○	○	○	○	○
Lourdeur	○	○	○	○	○	○	○	○	○	○	○
Sensibilité	○	○	○	○	○	○	○	○	○	○	○
Douleur fendante	○	○	○	○	○	○	○	○	○	○	○
Fatigante-épuisante	○	○	○	○	○	○	○	○	○	○	○
Douleur nauséeuse	○	○	○	○	○	○	○	○	○	○	○
Effrayante	○	○	○	○	○	○	○	○	○	○	○
Punitive-cruelle	○	○	○	○	○	○	○	○	○	○	○
Chocs électriques	○	○	○	○	○	○	○	○	○	○	○
Glaciale	○	○	○	○	○	○	○	○	○	○	○
Perçante	○	○	○	○	○	○	○	○	○	○	○
Douleurs lors de touchers légers	○	○	○	○	○	○	○	○	○	○	○
Causant des démangeaisons	○	○	○	○	○	○	○	○	○	○	○
Picotements	○	○	○	○	○	○	○	○	○	○	○

Engourdissements	○	○	○	○	○	○	○	○	○	○	○

Si vous voulez ajouter des précisions à vos réponses précédentes, veuillez utiliser l'espace suivant : ________________________________

Histoire médicale

Un médecin a-t-il déjà diagnostiqué la cause de vos douleurs?	◯ Oui<br>◯ Non
Quel était le diagnostic?	☐ Syndrome du côlon irritable<br>☐ Maladie inflammatoire de l'intestin (p. ex. maladie de Crohn ou rectocolite hémorragique)<br>☐ Endométriose<br>☐ Fibrome(s)<br>☐ Kyste ovarien<br>☐ Atteinte inflammatoire/infection pelvienne<br>☐ Vessie douloureuse/cystite interstitielle (HORS infection bactérienne de la vessie)<br>☐ Stress<br>☐ Autre<br>((cochez toutes les réponses applicables))
Veuillez préciser cet autre diagnostic.	______________________________
Un médecin vous a-t-il déjà diagnostiqué un cancer ou une quelconque tumeur maligne?	◯ Oui ◯ Non
1er. Quel(s) type(s) de cancer (localisation primaire) avez-vous eu?	______________________________
Quand le diagnostic a-t-il été posé pour la première fois?	______________________________<br>(Âge lors du diagnostic initial (ans))
2e. Quel(s) type(s) de cancer (localisation primaire) avez-vous eu?	______________________________
Quand le diagnostic a-t-il été posé pour la première fois?	______________________________<br>(Âge lors du diagnostic initial (ans))
3e. Quel(s) type(s) de cancer (localisation primaire) avez-vous eu?	______________________________
Quand le diagnostic a-t-il été posé pour la première fois?	______________________________<br>(Âge lors du diagnostic initial (ans))

Avez-vous déjà eu l'un des problèmes de santé suivants?

	Oui	Non
Anxiété nécessitant un traitement ou une thérapie	◯	◯
Asthme	◯	◯
Maladie cardiovasculaire	◯	◯
Syndrome de fatigue chronique (SFC)	◯	◯
Dépression nécessitant un traitement ou une thérapie	◯	◯
Diabète	◯	◯
Fibromes utérins	◯	◯
Fibromyalgie	◯	◯
Maladie de Graves	◯	◯
Maladie de Hashimoto	◯	◯
Hypertension artérielle	◯	◯
Syndrome du côlon irritable (SCI)	◯	◯
Migraine	◯	◯
Sclérose en plaques	◯	◯
Vessie douloureuse/cystite interstitielle (EXCLUANT infection bactérienne de la vessie)	◯	◯
Atteinte inflammatoire pelvienne (AIP ou PID)	◯	◯
Syndrome des ovaires polykystiques	◯	◯
Trouble de la thyroïde	◯	◯

Avez-vous subi les opérations chirurgicales suivantes au cours de votre vie?

Ligature des trompes (stérilisation) ◯ Oui ◯ Non

À quel âge?

____
(années)

Appendicectomie (résection de l'appendice) ◯ Oui ◯ Non

À quel âge?

____
(années)

Hystérectomie (retrait de l'utérus) ◯ Oui ◯ Non

À quel âge?

____
(années)

Pour quelle raison? ______________________

Ovariectomie (retrait de l'ovaire) ◯ Oui ◯ Non

Si oui, combien de vos ovaires ont été retirés?
◯ Une
◯ Les deux
◯ Incertaine

À quel(s) âge(s) ? ______________________
(veuillez lister tous les âges séparés par des virgules)

Curetage utérin (après dilatation du col) ◯ Oui ◯ Non

Hystéroscopie (caméro dans l'utérus) ◯ Oui ◯ Non

À quel(s) âge(s)? ______________________
(veuillez lister tous les âges séparés par des virgules)

Combien de fois au total? ______________________

Cholécystectomie (retrait de la vésicule biliaire) ◯ Oui ◯ Non

À quel(s) âge(s)? ______________________
(veuillez lister tous les âges séparés par des virgules)

Colonoscopie/Sigmoïdoscopie (insertion d'une caméra dans l'intestin) ◯ Oui ◯ Non

À quel(s) âge(s)? ______________________
(veuillez lister tous les âges séparés par des virgules)

Combien de fois au total? ______________________

Pour quelle raison? ______________________

Laparoscopie (opération impliquant l'insertion d'une caméra dans l'abdomen). Veuillez lister séparément si vous en avez eu plus d'une.

Laparoscopie #1 ◯ Oui ◯ Non

À quel âge? ______________________

Pour quelle raison?	__________________________________
Laparoscopie #2	◯ Oui ◯ Non
À quel âge?	__________________________________
Pour quelle raison?	__________________________________
Laparoscopie #3	◯ Oui ◯ Non
À quel âge?	__________________________________
Pour quelle raison?	__________________________________
Laparoscopie #4	◯ Oui ◯ Non
À quel âge?	__________________________________
Pour quelle raison?	__________________________________
Laparoscopie #5 (ou le dernier si plus de 5)	◯ Oui ◯ Non
À quel âge?	__________________________________
Pour quelle raison?	__________________________________
Autre opération abdominale?	◯ Oui ◯ Non
Quel type d'autre opération abdominale?	__________________________________
À quel(s) âge(s)?	__________________________________<br>(Veuillez indiquer tous les âges séparés par des virgules)
Combien de fois au total?	__________________________________
Pour quelle raison?	__________________________________
Autre opération abdominale?	◯ Oui ◯ Non
Quel type d'autre opération abdominale?	__________________________________

À quel(s) âge(s)?	______________________ (Veuillez indiquer tous les âges séparés par des virgules)
Combien de fois au total?	______________________
Pour quelle raison?	______________________
Autre opération abdominale?	◯ Oui ◯ Non
Quel type d'autre opération abdominale?	______________________
À quel(s) âge(s)?	______________________ (Veuillez indiquer tous les âges séparés par des virgules)
Combien de fois au total?	______________________
Pour quelle raison?	______________________
Avez-vous connu l'un des problèmes suivants au cours des 3 derniers mois ?	☐ Saignement rectal ou sang dans les selles<br>☐ Moins de 3 passages de selles par semaine<br>☐ Plus de 3 passages de selles par jour<br>☐ Selles dures ou grumeleuses<br>☐ Selles molles ou liquides<br>☐ Nausées et/ou vomissement<br>☐ Effort intense lors du passage de selles<br>☐ Besoin urgent d'aller à la selle<br>☐ Sensation de vidage incomplet après le passage de selles<br>☐ Expulsion de mucus lors du passage de selles<br>☐ Sensation de trop-plein, ballonnement ou gonflement abdominal<br>☐ Crampes intestinales<br>(Cochez toutes les réponses applicables)
Une endométriose a-t-elle déjà été diagnostiquée chez vous par un médecin ou autre professionnel de santé?	◯ Oui ◯ Non
Quel âge aviez-vous lorsque les symptômes sont apparus pour la première fois?	______________________ (ans)
Quels symptômes, le cas échéant, vous ont conduit à consulter un professionnel de santé avant le diagnostic de votre endométriose?	☐ Douleur<br>☐ Infertilité<br>☐ Aucun symptôme<br>☐ Autre<br>((cochez toutes les réponses applicables))

Veuillez préciser autre symptôme :	______________________
Quel âge aviez-vous lorsque votre endométriose a été diagnostiquée?	______________________<br>(ans)
Avez-vous déjà subi une intervention chirurgicale pour rechercher une endométriose sans qu'aucune ne soit détectée?	◯ Oui ◯ Non
Quels symptômes ont amené à effectuer cette intervention chirurgicale?	◯ Douleur ◯ Infertilité<br>◯ Autre
Autre symptôme :	______________________<br>(Veuillez préciser)

Y a-t-il dans votre famille des femmes qui ont eu une endométriose diagnostiquée ou ont souffert de douleurs pelviennes chroniques?

	Mère	Sœur	Grand-mère, tante ou cousine du côté maternel	Grand-mère, tante ou cousine du côté paternel
Endométriose	☐	☐	☐	☐
Douleurs pelviennes chroniques	☐	☐	☐	☐

Avez-vous déjà eu une des infections suivantes?

☐ Chlamydia
☐ Gonorrhée
☐ Herpès (génital)
☐ Atteinte inflammatoire pelvienne (AIP ou PID)
☐ Syphilis
☐ Virus de l'immunodéficience humaine (VIH)
☐ Vaginose bactérienne
☐ Virus du papillome humain (VPH)
☐ Levures
☐ Hépatite A
☐ Hépatite B
☐ Hépatite C
☐ Autre
☐ Aucune
(Veuillez cocher toutes les réponses applicables)

Si autre, veuillez préciser :

____

Si vous voulez ajouter des précisions à vos réponses précédentes, veuillez utiliser l'espace suivant :

____

MÉDICAMENTS UTILISÉS

Les questions suivantes se réfèrent à tous les médicaments pris au cours de votre vie.

Veuillez nous indiquer tous les médicaments anti-douleurs, en vente libre ou sur ordonnance, que vous avez utilisés au moins une fois par semaine pendant une période de 3 mois ou plus.

Acétaminophène (tylenol)	◯ Oui ◯ Non
Pris actuellement?	◯ Oui ◯ Non
À quel âge avez-vous commencé à prendre régulièrement ce médicament?	______________ (ans)
Pour quelle douleur ce médicament a-t-il été utilisé?	◯ Douleur pelvienne ◯ Autre douleur ◯ Les deux
Combien de jours par semaine?	◯ 1 ◯ 2-3 ◯ 4-5 ◯ 6+
Combien de comprimés par semaine?	◯ 1-2 ◯ 3-5 ◯ 6-14 ◯ 15+

Dans les deux questions ci-dessous, prière d'entrer la durée totale d'utilisation de ce médicament (années + mois). Si vous utilisation est de 3 ans et 2 mois, indiquez : durée totale d'utilisation (années) : 3; durée totale d'utilisation (mois) : 2.

Durée totale d'utilisation (années)	______________ (années)
Durée totale d'utilisation (mois)	◯ 0 ◯ 1 ◯ 2 ◯ 3 ◯ 4 ◯ 5 ◯ 6 ◯ 7 ◯ 8 ◯ 9 ◯ 10 ◯ 11 ◯ 12 (mois)
Aspirine (325 mg ou plus/comprimé)	◯ Oui ◯ Non
Pris actuellement?	◯ Oui ◯ Non
À quel âge avez-vous commencé à prendre régulièrement ce médicament?	______________ (ans)
Pour quelle douleur ce médicament a-t-il été utilisé?	◯ Douleur pelvienne ◯ Autre douleur ◯ Les deux
Combien de jours par semaine?	◯ 1 ◯ 2-3 ◯ 4-5 ◯ 6+
Combien de comprimés par semaine?	◯ 1-2 ◯ 3-5 ◯ 6-14 ◯ 15+

Dans les deux questions ci-dessous, prière d'entrer la durée totale d'utilisation de ce médicament (années + mois). Si vous utilisation est de 3 ans et 2 mois, indiquez : durée totale d'utilisation (années) : 3; durée totale d'utilisation (mois) : 2.

Powered by REDCap

Durée d'utilisation (années) :

____
(années)

Durée d'utilisation (mois) :

◯ 0
◯ 1
◯ 2
◯ 3
◯ 4
◯ 5
◯ 6
◯ 7
◯ 8
◯ 9
◯ 10
◯ 11
◯ 12
(mois)

Ibuprofène (ex. Motrin, Advil) ◯ Oui ◯ Non

Pris actuellement? ◯ Oui ◯ Non

À quel âge avez-vous commencé à prendre régulièrement ce médicament?

____
(ans)

Pour quelle douleur ce médicament a-t-il été utilisé?

◯ Douleur pelvienne ◯ Autre douleur
◯ Les deux

Combien de jours par semaine? ◯ 1 ◯ 2-3 ◯ 4-5 ◯ 6+

Combien de comprimés par semaine? ◯ 1-2 ◯ 3-5 ◯ 6-14
◯ 15+

Dans les deux questions ci-dessous, prière d'entrer la durée totale d'utilisation de ce médicament (années + mois). Si vous utilisation est de 3 ans et 2 mois, indiquez : durée totale d'utilisation (années) : 3; durée totale d'utilisation (mois) : 2.

Durée d'utilisation (années) :

____
(années)

Durée d'utilisation (mois) :

◯ 0
◯ 1
◯ 2
◯ 3
◯ 4
◯ 5
◯ 6
◯ 7
◯ 8
◯ 9
◯ 10
◯ 11
◯ 12
(mois)

Celebrex, Vioxx (inhibiteurs de la COX-2)	◯ Oui ◯ Non
Pris actuellement?	◯ Oui ◯ Non
À quel âge avez-vous commencé à prendre régulièrement ce médicament?	______________________ (ans)
Pour quelle douleur ce médicament a-t-il été utilisé?	◯ Douleur pelvienne ◯ Autre douleur ◯ Les deux
Combien de jours par semaine?	◯ 1 ◯ 2-3 ◯ 4-5 ◯ 6+
Combien de comprimés par semaine?	◯ 1-2 ◯ 3-5 ◯ 6-14 ◯ 15+

Dans les deux questions ci-dessous, prière d'entrer la durée totale d'utilisation de ce médicament (années + mois). Si vous utilisation est de 3 ans et 2 mois, indiquez : durée totale d'utilisation (années) : 3; durée totale d'utilisation (mois) : 2.

Durée d'utilisation (années) :	______________________ (années)
Durée d'utilisation (mois) :	◯ 0<br>◯ 1<br>◯ 2<br>◯ 3<br>◯ 4<br>◯ 5<br>◯ 6<br>◯ 7<br>◯ 8<br>◯ 9<br>◯ 10<br>◯ 11<br>◯ 12<br>(mois)
Autres analgésiques anti-inflammatoires (Naprosyn, Naproxène, acide méfanamique, Aleve, Relafen, Kétoprofène, Anaprox)	◯ Oui ◯ Non
Pris actuellement?	◯ Oui ◯ Non
À quel âge avez-vous commencé à prendre régulièrement ce médicament ?	______________________ (ans)
Pour quelle douleur ce médicament a-t-il été utilisé?	◯ Douleur pelvienne ◯ Autre douleur ◯ Les deux
Combien de jours par semaine?	◯ 1 ◯ 2-3 ◯ 4-5 ◯ 6+

Combien de comprimés par semaine?	◯ 1-2 ◯ 3-5 ◯ 6-14<br>◯ 15+

Dans les deux questions ci-dessous, prière d'entrer la durée totale d'utilisation de ce médicament (années + mois). Si vous utilisation est de 3 ans et 2 mois, indiquez : durée totale d'utilisation (années) : 3; durée totale d'utilisation (mois) : 2.

Durée d'utilisation (années) :	_________________________________<br>(années)
Durée d'utilisation (mois) :	◯ 0 ◯ 1 ◯ 2 ◯ 3<br>◯ 4 ◯ 5 ◯ 6 ◯ 7<br>◯ 8 ◯ 9 ◯ 10 ◯ 11<br>◯ 12<br>(mois)
Analgésiques puissants (narcotiques) (dilaudid, morphine, codéine, oxycodone, hydrocodone + paracétamol, codéine + paracétamol, , hydrocodone, Demerol)	◯ Oui ◯ Non
Pris actuellement?	◯ Oui ◯ Non
À quel âge avez-vous commencé à prendre régulièrement ce médicament?	_________________________________<br>(ans)
Pour quelle douleur ce médicament a-t-il été utilisé?	◯ Douleur pelvienne ◯ Autre douleur<br>◯ Les deux
Combien de jours par semaine?	◯ 1 ◯ 2-3 ◯ 4-5 ◯ 6+
Combien de comprimés par semaine?	◯ 1-2 ◯ 3-5 ◯ 6-14<br>◯ 15+

Dans les deux questions ci-dessous, prière d'entrer la durée totale d'utilisation de ce médicament (années + mois). Si vous utilisation est de 4 ans et 0 mois, indiquez : durée totale d'utilisation (années) : 4; durée totale d'utilisation (mois) : 0.

Durée d'utilisation (années) :	_________________________________<br>(années)
Durée d'utilisation (mois) :	◯ 0 ◯ 1 ◯ 2 ◯ 3<br>◯ 4 ◯ 5 ◯ 6 ◯ 7<br>◯ 8 ◯ 9 ◯ 10 ◯ 11<br>◯ 12
Prenez-vous des opioïdes/narcotiques (ex. : Tylenol#3, Tramacet, oxycodone, morphine) TOUS LES JOURS en raison de la douleur pelvienne?	◯ Oui ◯ Non
Autres anti-douleurs ciblant les nerfs/le système nerveux central (amitriptyline-Elavil, nortriptyline, gabapentine-Neurontin, prégabaline, lamotrigine)	◯ Oui ◯ Non

Pris actuellement?	◯ Oui ◯ Non
À quel âge avez-vous commencé à prendre régulièrement ce médicament?	______________________ (ans)
Pour quelle douleur ce médicament a-t-il été utilisé?	◯ Douleur pelvienne ◯ Autre douleur ◯ Les deux
Combien de jours par semaine?	◯ 1 ◯ 2-3 ◯ 4-5 ◯ 6+
Combien de comprimés par semaine?	◯ 1-2 ◯ 3-5 ◯ 6-14 ◯ 15+

Dans les deux questions ci-dessous, prière d'entrer la durée totale d'utilisation de ce médicament (années + mois). Si vous utilisation est de 4 ans et 0 mois, indiquez : durée totale d'utilisation (années) : 4; durée totale d'utilisation (mois) : 0.

Durée d'utilisation (années) :	______________________ (années)
Durée d'utilisation (mois) :	◯ 0 ◯ 1 ◯ 2 ◯ 3 ◯ 4 ◯ 5 ◯ 6 ◯ 7 ◯ 8 ◯ 9 ◯ 10 ◯ 11 ◯ 12 (mois)
Relaxants musculaires (diazépam/témazépam, buscopan)	◯ Oui ◯ Non
Pris actuellement?	◯ Oui ◯ Non
À quel âge avez-vous commencé à prendre régulièrement ce médicament?	______________________ (ans)
Pour quelle douleur ce médicament a-t-il été utilisé?	◯ Douleur pelvienne ◯ Autre douleur ◯ Les deux
Combien de jours par semaine?	◯ 1 ◯ 2-3 ◯ 4-5 ◯ 6+
Combien de comprimés par semaine?	◯ 1-2 ◯ 3-5 ◯ 6-14 ◯ 15+

Dans les deux questions ci-dessous, prière d'entrer la durée totale d'utilisation de ce médicament (années + mois). Si vous utilisation est de 4 ans et 0 mois, indiquez : durée totale d'utilisation (années) : 4; durée totale d'utilisation (mois) : 0.

Durée d'utilisation (années) :	______________________ (années)

Durée d'utilisation (mois)	◯ 0 ◯ 1 ◯ 2 ◯ 3<br>◯ 4 ◯ 5 ◯ 6 ◯ 7<br>◯ 8 ◯ 9 ◯ 10 ◯ 11<br>◯ 12<br>(mois)
Produits de santé naturels	◯ Oui ◯ Non
Pris actuellement?	◯ Oui<br>◯ Non
À quel âge avez-vous commencé à prendre régulièrement ce produit?	__________________________________<br>(ans)
Pour quelle douleur ce produit a-t-il été utilisé?	◯ Douleur pelvienne<br>◯ Autre douleur<br>◯ Les deux
Combien de jours par semaine?	◯ 1<br>◯ 2-3<br>◯ 4-5<br>◯ 6+
Combien de comprimés par semaine?	◯ 1-2<br>◯ 3-5<br>◯ 6-14<br>◯ 15+

Dans les deux questions ci-dessous, prière d'entrer la durée totale d'utilisation de ce médicament (années + mois). Si vous utilisation est de 4 ans et 0 mois, indiquez : durée totale d'utilisation (années) : 4; durée totale d'utilisation (mois) : 0.

Durée d'utilisation (années) :	__________________________________<br>(années)
Durée d'utilisation (mois) :	◯ 0<br>◯ 1<br>◯ 2<br>◯ 3<br>◯ 4<br>◯ 5<br>◯ 6<br>◯ 7<br>◯ 8<br>◯ 9<br>◯ 10<br>◯ 11<br>◯ 12<br>(mois)

Avez-vous déjà pris des médicaments sur ordonnance pendant plus de 3 mois, en excluant les traitements hormonaux et les anti-douleurs?

Diurétique	◯ Oui ◯ Non

À quel âge avez-vous commencé à prendre ce médicament quotidiennement pendant plus d'un mois?	__________ (ans)
Pendant combien d'années, au total, avez-vous pris ce médicament? Veuillez donner une estimation, et indiquez '0 an au total' si moins de 1 an.	__________ (années de prise)
Prenez-vous actuellement ce médicament quotidiennement?	◯ Oui ◯ Non
Veuillez indiquer le nom exact du médicament utilisé le plus récemment si vous le connaissez :	__________
Comprimés pour le diabète	◯ Oui ◯ Non
À quel âge avez-vous commencé à prendre ce médicament quotidiennement pendant plus d'un mois?	__________ (ans)
Pendant combien d'années, au total, avez-vous pris ce médicament? Veuillez donner une estimation, et indiquez '0 an au total' si moins de 1 an.	__________ (années de prise)
Prenez-vous actuellement ce médicament quotidiennement?	◯ Oui ◯ Non
Veuillez indiquer le nom exact du médicament utilisé le plus récemment si vous le connaissez :	__________
Insuline	◯ Oui ◯ Non
À quel âge avez-vous commencé à prendre ce médicament quotidiennement pendant plus d'un mois?	__________ (ans)
Pendant combien d'années, au total, avez-vous pris ce médicament? Veuillez donner une estimation, et indiquez '0 an au total' si moins de 1 an.	__________ (années de prise)
Prenez-vous actuellement ce médicament quotidiennement?	◯ Oui ◯ Non
Veuillez indiquer le nom exact du médicament utilisé le plus récemment si vous le connaissez :	__________
Médicaments pour la thyroïde	◯ Oui ◯ Non
À quel âge avez-vous commencé à prendre ce médicament quotidiennement pendant plus d'un mois?	__________ (ans)
Pendant combien d'années, au total, avez-vous pris ce médicament? Veuillez donner une estimation, et indiquez '0 an au total' si moins de 1 an.	__________ (années de prise)

Prenez-vous actuellement ce médicament quotidiennement?	◯ Oui ◯ Non
Veuillez indiquer le nom exact du médicament utilisé le plus récemment si vous le connaissez :	__________________
Médicaments contre l'épilepsie	◯ Oui ◯ Non
À quel âge avez-vous commencé à prendre ce médicament quotidiennement pendant plus d'un mois?	__________________ (ans)
Pendant combien d'années, au total, avez-vous pris ce médicament? Veuillez donner une estimation, et indiquez '0 an au total' si moins de 1 an.	__________________ (années de prise)
Prenez-vous actuellement ce médicament quotidiennement?	◯ Oui ◯ Non
Veuillez indiquer le nom exact du médicament utilisé le plus récemment si vous le connaissez :	__________________
Comprimés pour dormir/tranquillisants	◯ Oui ◯ Non
À quel âge avez-vous commencé à prendre ce médicament quotidiennement pendant plus d'un mois?	__________________ (ans)
Pendant combien d'années, au total, avez-vous pris ce médicament? Veuillez donner une estimation, et indiquez '0 an au total' si moins de 1 an.	__________________ (années de prise)
Prenez-vous actuellement ce médicament quotidiennement?	◯ Oui ◯ Non
Veuillez indiquer le nom exact du médicament utilisé le plus récemment si vous le connaissez :	__________________
Anti-dépresseurs	◯ Oui ◯ Non
À quel âge avez-vous commencé à prendre ce médicament quotidiennement pendant plus d'un mois?	__________________ (ans)
Pendant combien d'années, au total, avez-vous pris ce médicament? Veuillez donner une estimation, et indiquez '0 an au total' si moins de 1 an.	__________________ (années de prise)
Prenez-vous actuellement ce médicament quotidiennement?	◯ Oui ◯ Non
Veuillez indiquer le nom exact du médicament utilisé le plus récemment si vous le connaissez :	__________________
Autres médicaments pour traiter les troubles mentaux :	◯ Oui ◯ Non

À quel âge avez-vous commencé à prendre ce médicament quotidiennement pendant plus d'un mois?	__________ (ans)
Pendant combien d'années, au total, avez-vous pris ce médicament? Veuillez donner une estimation, et indiquez '0 an au total' si moins de 1 an.	__________ (années de prise)
Prenez-vous actuellement ce médicament quotidiennement?	◯ Oui ◯ Non
Veuillez indiquer le nom exact du médicament utilisé le plus récemment si vous le connaissez :	__________
Médicaments contre l'ostéoporose (os fragiles)	◯ Oui ◯ Non
À quel âge avez-vous commencé à prendre ce médicament quotidiennement pendant plus d'un mois?	__________ (ans)
Pendant combien d'années, au total, avez-vous pris ce médicament? Veuillez donner une estimation, et indiquez '0 an au total' si moins de 1 an.	__________ (années de prise)
Prenez-vous actuellement ce médicament quotidiennement?	◯ Oui ◯ Non
Veuillez indiquer le nom exact du médicament utilisé le plus récemment si vous le connaissez :	__________
Médicaments contre la polyarthrite rhumatoïde	◯ Oui ◯ Non
À quel âge avez-vous commencé à prendre ce médicament quotidiennement pendant plus d'un mois?	__________ (ans)
Pendant combien d'années, au total, avez-vous pris ce médicament? Veuillez donner une estimation, et indiquez '0 an au total' si moins de 1 an.	__________ (années de prise)
Prenez-vous actuellement ce médicament quotidiennement?	◯ Oui ◯ Non
Veuillez indiquer le nom exact du médicament utilisé le plus récemment si vous le connaissez :	__________
Antibiotiques pendant un mois ou plus	◯ Oui ◯ Non
À quel âge avez-vous commencé à prendre ce médicament quotidiennement pendant plus d'un mois?	__________ (ans)
Pendant combien d'années, au total, avez-vous pris ce médicament? Veuillez donner une estimation, et indiquez '0 an au total' si moins de 1 an.	__________ (années de prise)

Prenez-vous actuellement ce médicament quotidiennement?	◯ Oui ◯ Non
Veuillez indiquer le nom exact du médicament utilisé le plus récemment si vous le connaissez :	__________________
Anti-acides	◯ Oui ◯ Non
À quel âge avez-vous commencé à prendre ce médicament quotidiennement pendant plus d'un mois?	__________________ (ans)
Pendant combien d'années, au total, avez-vous pris ce médicament? Veuillez donner une estimation, et indiquez '0 an au total' si moins de 1 an.	__________________ (années de prise)
Prenez-vous actuellement ce médicament quotidiennement?	◯ Oui ◯ Non
Veuillez indiquer le nom exact du médicament utilisé le plus récemment si vous le connaissez :	__________________
Médicaments contre l'ulcère de l'estomac / la gastrite	◯ Oui ◯ Non
À quel âge avez-vous commencé à prendre ce médicament quotidiennement pendant plus d'un mois?	__________________ (ans)
Pendant combien d'années, au total, avez-vous pris ce médicament? Veuillez donner une estimation, et indiquez '0 an au total' si moins de 1 an.	__________________ (années de prise)
Prenez-vous actuellement ce médicament quotidiennement?	◯ Oui ◯ Non
Veuillez indiquer le nom exact du médicament utilisé le plus récemment si vous le connaissez :	__________________
Médicaments pour faire baisser le cholestérol	◯ Oui ◯ Non
À quel âge avez-vous commencé à prendre ce médicament quotidiennement pendant plus d'un mois?	__________________ (ans)
Pendant combien d'années, au total, avez-vous pris ce médicament? Veuillez donner une estimation, et indiquez '0 an au total' si moins de 1 an.	__________________ (années de prise)
Prenez-vous actuellement ce médicament quotidiennement?	◯ Oui ◯ Non
Veuillez indiquer le nom exact du médicament utilisé le plus récemment si vous le connaissez :	__________________

Médicaments contre les allergies (antihistaminiques)	◯ Oui ◯ Non
À quel âge avez-vous commencé à prendre ce médicament quotidiennement pendant plus d'un mois?	______________ (ans)
Pendant combien d'années, au total, avez-vous pris ce médicament? Veuillez donner une estimation, et indiquez '0 an au total' si moins de 1 an.	______________ (années de prise)
Prenez-vous actuellement ce médicament quotidiennement?	◯ Oui ◯ Non
Veuillez indiquer le nom exact du médicament utilisé le plus récemment si vous le connaissez :	______________
Stéroïdes (oraux, inhalés ou intra-nasaux)	◯ Oui ◯ Non
À quel âge avez-vous commencé à prendre ce médicament quotidiennement pendant plus d'un mois?	______________ (ans)
Pendant combien d'années, au total, avez-vous pris ce médicament? Veuillez donner une estimation, et indiquez '0 an au total' si moins de 1 an.	______________ (années de prise)
Prenez-vous actuellement ce médicament quotidiennement?	◯ Oui ◯ Non
Veuillez indiquer le nom exact du médicament utilisé le plus récemment si vous le connaissez :	______________
Chimiothérapie contre le cancer	◯ Oui ◯ Non
À quel âge avez-vous commencé à prendre ce médicament quotidiennement pendant plus d'un mois?	______________ (ans)
Pendant combien d'années, au total, avez-vous pris ce médicament? Veuillez donner une estimation, et indiquez '0 an au total' si moins de 1 an.	______________ (années de prise)
Prenez-vous actuellement ce médicament quotidiennement?	◯ Oui ◯ Non
Veuillez indiquer le nom exact du médicament utilisé le plus récemment si vous le connaissez :	______________
Tamoxifène contre le cancer	◯ Oui ◯ Non
À quel âge avez-vous commencé à prendre ce médicament quotidiennement pendant plus d'un mois?	______________ (ans)

Pendant combien d'années, au total, avez-vous pris ce médicament? Veuillez donner une estimation, et indiquez '0 an au total' si moins de 1 an.	______________ (années de prise)
Prenez-vous actuellement ce médicament quotidiennement?	◯ Oui ◯ Non
Veuillez indiquer le nom exact du médicament utilisé le plus récemment si vous le connaissez :	______________
Médicaments pour la tension artérielle	◯ Oui ◯ Non
À quel âge avez-vous commencé à prendre ce médicament quotidiennement pendant plus d'un mois?	______________ (ans)
Pendant combien d'années, au total, avez-vous pris ce médicament? Veuillez donner une estimation, et indiquez '0 an au total' si moins de 1 an.	______________ (années de prise)
Prenez-vous actuellement ce médicament quotidiennement?	◯ Oui ◯ Non
Veuillez indiquer le nom exact du médicament utilisé le plus récemment si vous le connaissez :	______________
Médicaments contre l'angine de poitrine (douleur thoracique)	◯ Oui ◯ Non
À quel âge avez-vous commencé à prendre ce médicament quotidiennement pendant plus d'un mois?	______________ (ans)
Pendant combien d'années, au total, avez-vous pris ce médicament? Veuillez donner une estimation, et indiquez '0 an au total' si moins de 1 an.	______________ (années de prise)
Prenez-vous actuellement ce médicament quotidiennement?	◯ Oui ◯ Non
Veuillez indiquer le nom exact du médicament utilisé le plus récemment si vous le connaissez :	______________
Autres médicaments pour une maladie cardiaque	◯ Oui ◯ Non
À quel âge avez-vous commencé à prendre ce médicament quotidiennement pendant plus d'un mois?	______________ (ans)
Pendant combien d'années, au total, avez-vous pris ce médicament? Veuillez donner une estimation, et indiquez '0 an au total' si moins de 1 an.	______________ (années de prise)
Prenez-vous actuellement ce médicament quotidiennement?	◯ Oui ◯ Non

Veuillez indiquer le nom exact du médicament utilisé le plus récemment si vous le connaissez :	______________________
Pompe pour l'asthme	◯ Oui ◯ Non
À quel âge avez-vous commencé à prendre ce médicament quotidiennement pendant plus d'un mois?	______________________ (ans)
Pendant combien d'années, au total, avez-vous pris ce médicament? Veuillez donner une estimation, et indiquez '0 an au total' si moins de 1 an.	______________________ (années de prise)
Prenez-vous actuellement ce médicament quotidiennement?	◯ Oui ◯ Non
Veuillez indiquer le nom exact du médicament utilisé le plus récemment si vous le connaissez :	______________________
Coumadin / héparine pour fluidifier le sang	◯ Oui ◯ Non
À quel âge avez-vous commencé à prendre ce médicament quotidiennement pendant plus d'un mois?	______________________ (ans)
Pendant combien d'années, au total, avez-vous pris ce médicament? Veuillez donner une estimation, et indiquez '0 an au total' si moins de 1 an.	______________________ (années de prise)
Prenez-vous actuellement ce médicament quotidiennement?	◯ Oui ◯ Non
Veuillez indiquer le nom exact du médicament utilisé le plus récemment si vous le connaissez :	______________________
Comprimés/injections contre les migraines	◯ Oui ◯ Non
À quel âge avez-vous commencé à prendre ce médicament quotidiennement pendant plus d'un mois?	______________________ (ans)
Pendant combien d'années, au total, avez-vous pris ce médicament? Veuillez donner une estimation, et indiquez '0 an au total' si moins de 1 an.	______________________ (années de prise)
Prenez-vous actuellement ce médicament quotidiennement?	◯ Oui ◯ Non
Veuillez indiquer le nom exact du médicament utilisé le plus récemment si vous le connaissez :	______________________
Autre #1	◯ Oui ◯ Non

Powered by REDCap

À quel âge avez-vous commencé à prendre ce médicament quotidiennement pendant plus d'un mois?	__________ (ans)
Pendant combien d'années, au total, avez-vous pris ce médicament? Veuillez donner une estimation, et indiquez '0 an au total' si moins de 1 an.	__________ (années de prise)
Prenez-vous actuellement ce médicament quotidiennement?	◯ Oui ◯ Non
Veuillez indiquer le nom exact du médicament utilisé le plus récemment si vous le connaissez :	__________
Autre #2	◯ Oui ◯ Non
À quel âge avez-vous commencé à prendre ce médicament quotidiennement pendant plus d'un mois?	__________ (ans)
Pendant combien d'années, au total, avez-vous pris ce médicament? Veuillez donner une estimation, et indiquez '0 an au total' si moins de 1 an.	__________ (années de prise)
Prenez-vous actuellement ce médicament quotidiennement?	◯ Oui ◯ Non
Veuillez indiquer le nom exact du médicament utilisé le plus récemment si vous le connaissez :	__________
Autre #3	◯ Oui ◯ Non
À quel âge avez-vous commencé à prendre ce médicament quotidiennement pendant plus d'un mois?	__________ (ans)
Pendant combien d'années, au total, avez-vous pris ce médicament? Veuillez donner une estimation, et indiquez '0 an au total' si moins de 1 an.	__________ (années de prise)
Prenez-vous actuellement ce médicament quotidiennement?	◯ Oui ◯ Non
Veuillez indiquer le nom exact du médicament utilisé le plus récemment si vous le connaissez :	__________
Autre #4	◯ Oui ◯ Non
À quel âge avez-vous commencé à prendre ce médicament quotidiennement pendant plus d'un mois?	__________
Pendant combien d'années, au total, avez-vous pris ce médicament? Veuillez donner une estimation, et indiquez '0 an au total' si moins de 1 an.	__________ (années de prise)

Prenez-vous actuellement ce médicament quotidiennement?	◯ Oui ◯ Non
Veuillez indiquer le nom exact du médicament utilisé le plus récemment si vous le connaissez :	__________________________________
Autre #5	◯ Oui ◯ Non
À quel âge avez-vous commencé à prendre ce médicament quotidiennement pendant plus d'un mois?	__________________________________ (ans)
Pendant combien d'années, au total, avez-vous pris ce médicament? Veuillez donner une estimation, et indiquez '0 an au total' si moins de 1 an.	__________________________________ (années de prise)
Prenez-vous actuellement ce médicament quotidiennement?	◯ Oui ◯ Non
Veuillez indiquer le nom exact du médicament utilisé le plus récemment si vous le connaissez :	__________________________________

Informations personnelles

Quelle est votre orientation sexuelle?

- ◯ Hétérosexuelle
- ◯ Lesbienne
- ◯ Bisexuelle
- ◯ Asexuelle
- ◯ Aucun des choix ci-dessus
- ◯ Je préfère ne pas répondre.

Veuillez spécifier votre orientation sexuelle :

____

Comment décririez-vous votre identité de genre?

- ◯ Femme
- ◯ Homme
- ◯ Transgenre - Femme à Homme
- ◯ Transgenre - Homme à Femme
- ◯ Non-Binaire / Queer
- ◯ Aucun des choix ci-dessus
- ◯ Je préfère ne pas répondre

Veuillez spécifier votre identité de genre :

____

État civil

- ◯ Célibataire
- ◯ En relation
- ◯ Mariée
- ◯ Conjointe
- ◯ Séparée
- ◯ Divorcée
- ◯ Veuve

Comment décririez-vous votre origine ethnique/culturelle?

- ☐ Canadienne-Nord Américaine
- ☐ Autochtone-Inuit
- ☐ Amérique latine-centrale-du sud
- ☐ Européenne de l'Ouest-Est
- ☐ Africaine
- ☐ Asiatique
- ☐ Autres

(Veuillez cocher toutes les réponses applicables)

Si autre, veuillez spécifier :

____

Travaillez-vous actuellement?

◯ Oui ◯ Non

Quelle est votre type de professions?
Veuillez vous référer aux définitions ci-dessous.

- ◯ Gestion
- ◯ Affaires, finance et administration
- ◯ Sciences naturelles et appliquées et professions apparentées
- ◯ Secteur de la santé
- ◯ Enseignement, droit et services sociaux, communautaires et gouvernementaux
- ◯ Arts, culture, sports et loisirs
- ◯ Ventes et services
- ◯ Métiers, transport, machinerie et domaines apparentés
- ◯ Ressources naturelles, agriculture et production connexe
- ◯ Fabrication et services d'utilité publique

A - Gestion
Cette catégorie comprend les membres de corps législatifs et les cadres supérieurs et intermédiaires. Les cadres supérieurs sont décrits dans plusieurs grandes catégories.
B - Affaires, finance et administration
Cette catégorie comprend les professions qui touchent la prestation de services financiers et d'affaires, de services administratifs et de services de supervision de bureau et de soutien.
C - Sciences naturelles et appliquées et domaines apparentés
Cette catégorie comprend les professions en sciences, en génie, en architecture et en technologie de l'information.
D - Secteur de la santé
Cette catégorie comprend les professions touchant la prestation directe de services de soins de santé aux patients, ainsi que les professions de soutien technique au personnel médical.
E - Enseignement, droit et services sociaux, communautaires et gouvernementaux
Cette catégorie englobe les professions touchant au droit, à l'enseignement, au counseling, à la recherche en sciences sociales, à l'élaboration de politiques publiques et à l'administration de programmes gouvernementaux et de programmes des autres secteurs. Elle comprend aussi les professions touchant le leadership et les services religieux.
F - Arts, culture, sports et loisirs
Cette catégorie englobe les professions des arts et de la culture, y compris celles touchant les arts du spectacle, le film et la vidéo, la radiotélédiffusion, le journalisme, la rédaction, le design créatique, la bibliothéconomie et la muséologie. Elle comprend également les professions en sports et en loisirs.
G - Ventes et services
Cette catégorie comprend les professions dans les domaines de la vente en gros et au détail, des services personnels et de protection et les professions liées à l'accueil et au tourisme.
H - Métiers, transport, machinerie et domaines apparentés
Cette catégorie regroupe les superviseurs de métiers et les contremaîtres, les personnes de métier en construction et en mécanique, les opérateurs et les conducteurs de matériel de transport et de machinerie lourde, ainsi que les aides de corps de métiers.
I - Ressources naturelles, agriculture et production connexe
Cette catégorie englobe les postes de supervision et de conduite de machines dans les secteurs d'exploitation des ressources naturelles, c'est-à-dire la production minière, pétrolière et gazière, la foresterie et l'abattage, l'agriculture, l'horticulture et la pêche.
J - Fabrication et services d'utilité publique
Cette catégorie englobe les postes de supervision, de production et de main d'oeuvre dans les secteurs de la transformation, de la fabrication et des services d'utilité publique.

Si non, est-ce en raison des douleurs pelviennes? ◯ Oui ◯ Non

---

Êtes-vous aux études actuellement? ◯ Oui ◯ Non

Quel est le plus haut niveau de scolarité que vous avez complété?

- ◯ Aucune scolarité (ou niveau primaire non complété)
- ◯ Primaire
- ◯ Secondaire (ex. secondaire 5, DEP, etc.)
- ◯ Collégial (ex. DEC général ou technique, AEC, etc.)
- ◯ Universitaire (ex. certificat, baccalauréat, maîtrise, doctorat, etc.)
- ◯ Je préfère ne pas répondre

Quel était approximativement votre revenu familial total l'an dernier avant déduction d'impôts?

- ◯ 0$ - 19 999$
- ◯ 20 000$ - 39 999$
- ◯ 40 000$ - 59 999$
- ◯ 60 000$ - 79 999$
- ◯ 80 000$ - 99 999$
- ◯ 100 000$ et plus
- ◯ Je préfère ne pas répondre

Dans les prochaines questions, veuillez indiquer votre taille dans l'unité de votre choix (pieds et pouces ou centimètres).

Quelle est votre taille en pieds?
- ◯ 3
- ◯ 4
- ◯ 5
- ◯ 6

Quelle est votre taille en pouces?
- ◯ 0
- ◯ 1
- ◯ 2
- ◯ 3
- ◯ 4
- ◯ 5
- ◯ 6
- ◯ 7
- ◯ 8
- ◯ 9
- ◯ 10
- ◯ 11

Quelle est votre taille en cm?

________________________________ (cm)

Dans les prochaines questions, veuillez indiquer votre poids dans l'unité de votre choix (livres ou kilogrammes).

Quel est votre poids actuel (livres)?

________________________________ ((livres))

Quel est votre poids actuel (en kg)?

________________________________ ((kg))

À l'âge de 18 ans, quelle était votre couleur de cheveux naturelle? Si vous avez moins de 18 ans, indiquez-nous votre couleur de cheveux naturelle actuelle. "
- ◯ Roux
- ◯ Blonds
- ◯ Châtains
- ◯ Bruns
- ◯ Noirs

De quelle couleur sont vos yeux?
- ◯ Bleu
- ◯ Gris
- ◯ Vert
- ◯ Noisette
- ◯ Brun
- ◯ Pers
- ◯ Vairons (yeux de couleurs différentes)

Avez-vous fumé plus de 100 cigarettes dans votre vie? ◯ Oui ◯ Non

Fumez-vous actuellement? ◯ Oui ◯ Non

Nombre de cigarettes fumées par semaine

________________________________ ((cigarettes par semaine))

Buvez-vous de l'alcool? ◯ Oui ◯ Non

Combien de consommations d'alcool prenez-vous en moyenne par semaine?<br>Une consommation équivaut à : 1 bouteille/canette de bière (340 ml, 12 oz), 1 verre de vin (140ml, 5 oz), 1 verre de vin fortifié 20% alcool (85 ml, 3 oz). 1 verre de spiritueux 40% alcool (45 ml, 5 oz).	______________________<br>(consommations / par semaine)
Consommez-vous de la marijuana (cannabis)?	◯ Je n'ai jamais consommé de cannabis<br>◯ Dans le passé, occasionnellement<br>◯ Dans le passé, régulièrement<br>◯ Oui, occasionnellement<br>◯ Oui, régulièrement ◯ Je préfère ne pas répondre
Consommez-vous des drogues illicites?	◯ Je n'ai jamais consommé de drogues.<br>◯ Dans le passé, occasionnellement<br>◯ Dans le passé, régulièrement<br>◯ Oui, occasionnellement<br>◯ Oui, régulièrement ◯ Je préfère ne pas répondre
Quelle(s) drogue(s) consommez-vous actuellement?	☐ Héroïne<br>☐ Amphétamines<br>☐ Barbiturique<br>☐ Cocaïne<br>☐ Je préfère ne pas répondre<br>☐ Autre
Si autre, veuillez préciser :	______________________

MODE DE VIE

Au cours des 12 derniers mois, combien de temps par semaine en moyenne avez-vous consacré à chacune des activités de loisir suivantes ?

	0	1-4 min	5-19 min	20-59 min	1 heure	1-1.5 hrs	2-3 hrs	4-6 hrs	7-10 hrs	11+hrs
Marche ou randonnée (y compris la marche pour aller au travail)	◯	◯	◯	◯	◯	◯	◯	◯	◯	◯
Jogging (moins de 10 km/heure)	◯	◯	◯	◯	◯	◯	◯	◯	◯	◯
Course rapide (10 km/h ou plus)	◯	◯	◯	◯	◯	◯	◯	◯	◯	◯
Vélo (y compris vélo d'appartement)	◯	◯	◯	◯	◯	◯	◯	◯	◯	◯
Gymnastique rythmique/aérobic/danse tonique/rameur	◯	◯	◯	◯	◯	◯	◯	◯	◯	◯
Tennis, squash, racquetball	◯	◯	◯	◯	◯	◯	◯	◯	◯	◯
Natation sportive	◯	◯	◯	◯	◯	◯	◯	◯	◯	◯
Autre activité tonique (p. ex., tondre la pelouse)	◯	◯	◯	◯	◯	◯	◯	◯	◯	◯

TRAUMA

Avez-vous déjà été témoin ou vécu un événement où vous, ou quelqu'un d'autre, vous êtes senties menacées ou en danger; ou que vous avez été violenté physiquement ou émotionnellement?	◯ Oui<br>◯ Non<br>◯ Je préfère ne pas répondre.

Avez- vous eu l'une des expériences suivants en tant qu'enfant (moins de 14 ans)?

Quelqu'un dans ma famille m'a frappé si fort que j'ai eu des marques ou des contusions (ecchymoses, bleus)?	◯ Oui ◯ Non
Quelqu'un dans ma famille m'a traité, de façon répétitive, de tous les noms (ex. stupide, paresseuse ou laide).	◯ Oui ◯ Non
J'ai senti qu'une personne de famille me haïssait.	◯ Oui ◯ Non
Quelqu'un a essayé de me toucher d'une façon sexuelle ou a essayé de m'obliger à le toucher.	◯ Oui ◯ Non
Quelqu'un a menacé de me faire mal ou de dire des mensonges sur moi si je faisais pas des choses sexuels avec eux.	◯ Oui ◯ Non

Avez- vous eu l'une des expériences suivants en tant qu'adulte (plus de 14 ans)?

Quelqu'un dans ma famille m'a frappé si fort que j'ai eu des marques ou des contusions (ecchymoses, bleus)?	◯ Oui ◯ Non
Quelqu'un m'a forcé à avoir un rapport sexuel sans mon consentement.	◯ Oui ◯ Non

Qualité de vie EHP

Dans les 4 DERNIÈRES SEMAINES, du fait de votre endométriose/douleurs pelviennes, dans quelle mesure avez-vous...

		Jamais	Rarement	Parfois	Souvent	Toujours
1)	Été incapable de faire des travaux domestiques (tâches ménagères, etc.) à cause de la douleur?					
2)	Trouvé difficile de rester debout à cause de la douleur?					
3)	Trouvé difficile de rester assise à cause de la douleur?					
4)	Trouvé difficile de marcher à cause de la douleur?					
5)	Trouvé difficile de faire de l'exercice ou les activités de loisirs que vous aimez à cause de la douleur?					
6)	Perdu l'appétit ou été incapable de manger à cause de la douleur?					
7)	Trouvé difficile de dormir normalement à cause de la douleur?					
8)	Été obligée de devoir aller au lit ou de vous allonger à cause de la douleur?					
9)	Été incapable de faire les choses que vous vouliez (travail, école, etc.) à cause de la douleur?					
10)	Trouvé difficile de vivre avec la douleur?					
11)	Été incapable d'avoir une vie sociale (sortir, aller à des événements sociaux, etc.) à cause de la douleur?					

Powered by REDCap

CSI

Date de complétion du questionnaire ____________________

CSI

Veuillez indiquer pour chaque situation la proposition la plus adaptée.

	Jamais	Rarement	Parfois	Souvent	Toujours
1. J'ai la sensation d'un sommeil non récupérateur quand je me réveille le matin	○	○	○	○	○
2. Je ressens des raideurs et des douleurs musculaires	○	○	○	○	○
3. Je fais des crises d'angoisse	○	○	○	○	○
4. Je grince ou serre les dents	○	○	○	○	○
5. J'ai des problèmes de diarrhée et/ou de constipation	○	○	○	○	○
6. J'ai besoin d'aide pour effectuer mes activités quotidiennes	○	○	○	○	○
7. Je suis sensible aux fortes lumières	○	○	○	○	○
8. Je me fatigue très facilement lorsque je suis actif physiquement	○	○	○	○	○
9. Je ressens des douleurs partout dans le corps	○	○	○	○	○
10. J'ai des maux de tête	○	○	○	○	○
11. Je ressens une gêne à la vessie et/ou des brûlures lorsque j'urine	○	○	○	○	○
12. Je ne dors pas bien	○	○	○	○	○
13. J'ai des difficultés de concentration	○	○	○	○	○
14. J'ai des problèmes de peau tels que sécheresse, démangeaisons ou éruptions cutanées	○	○	○	○	○
15. Le stress aggrave mes symptômes physiques	○	○	○	○	○
16. Je me sens triste ou déprimé	○	○	○	○	○
17. J'ai peu d'énergie	○	○	○	○	○

18. Je ressens des tensions musculaires dans la nuque et dans les épaules	○	○	○	○	○
19. J'ai mal à la mâchoire	○	○	○	○	○
20. Certaines odeurs, comme des parfums, me donnent des nausées et des étourdissements	○	○	○	○	○
21. Je dois uriner fréquemment	○	○	○	○	○
22. J'ai la sensation désagréable des jambes sans repos lorsque j'essaye de dormir le soir	○	○	○	○	○
23. J'ai des difficultés à me souvenir de certaines choses	○	○	○	○	○
24. J'ai eu des traumatismes au cours de mon enfance	○	○	○	○	○
25. Je ressens des douleurs dans la région du bassin	○	○	○	○	○

Douleur (PCS)

Veuillez compléter le questionnaire suivant.

Merci!

DOULEUR

Tout le monde fait l'expérience de la douleur à un moment ou un autre. Il peut s'agir, par exemple, de maux de tête, de douleurs dentaires, articulaires ou musculaires. Chacun est régulièrement exposé à des situations pouvant entraîner des douleurs, comme une maladie, une blessure, une intervention dentaire ou une opération chirurgicale. Nous aimerions mieux connaître vos pensées et sentiments lorsque vous ressentez des douleurs. La liste ci-dessous contient treize affirmations décrivant différents états d'esprit pouvant être associés à la douleur. À l'aide de l'échelle, veuillez indiquer dans quelle mesure vous vous trouvez dans ces états d'esprit lorsque vous ressentez des douleurs.

		Pas du tout	Un peu	Moyennement	Beaucoup	Tout le temps
1)	Je m'inquiète tout le temps de savoir si la douleur va s'arrêter	○	○	○	○	○
2)	J'ai le sentiment que je ne pourrai pas continuer	○	○	○	○	○
3)	C'est terrible et je me dis que ça ne va jamais aller mieux	○	○	○	○	○
4)	C'est épouvantable et je me sens accablée	○	○	○	○	○
5)	Je me dis que je ne pourrai pas le supporter plus longtemps	○	○	○	○	○
6)	Je suis effrayée à l'idée que la douleur ne s'aggrave	○	○	○	○	○
7)	Je n'arrête pas de penser à d'autres événements douloureux	○	○	○	○	○
8)	J'espère avec inquiétude que la douleur va disparaître	○	○	○	○	○
9)	Je n'arrive pas à ne plus y penser	○	○	○	○	○
10)	Je n'arrête pas de penser à quel point j'ai mal	○	○	○	○	○
11)	J'attends désespérément que la douleur s'arrête et je ne pense qu'à ça	○	○	○	○	○
12)	Il n'y a rien que je puisse faire pour atténuer la douleur	○	○	○	○	○
13)	Je me demande s'il ne risque pas de se passer quelque chose de grave	○	○	○	○	○

GAD-7

Veuillez compléter le questionnaire suivant.

Merci!

1) Date de complétion du questionnaire

____

GAD-7

Au cours des 14 derniers jours, à quelle fréquence avez-vous été dérangé(e) par les problèmes suivants?

	Jamais	Plusieurs jours	Plus de la moitié des jours	Presque tous les jours
2) Sentiment de nervosité, d'anxiété ou de tension	○	○	○	○
3) Incapable d'arrêter de vous inquiéter ou de contrôler vos inquiétudes	○	○	○	○
4) Inquiétudes excessives à propos de tout et de rien	○	○	○	○
5) Difficulté à se détendre	○	○	○	○
6) Agitation telle qu'il est difficile de rester tranquille	○	○	○	○
7) Devenir facilement contrarié(e) ou irritable	○	○	○	○
8) Avoir peur que quelque chose d'épouvantable puisse arrive	○	○	○	○

9) Si vous avez coché un problème parmi la liste ci-dessus, dans quelle mesure ces problèmes ont-ils rendu difficile pour vous de travailler, de vous occuper de vos tâches quotidiennes à la maison ou de vous entendre avec d'autres personnes?

- ○ Pas du tout difficile
- ○ Un peu difficile
- ○ Très difficile
- ○ Extrêmement difficile

Questionnaire santé du patient (PHQ-9)

Veuillez compléter le questionnaire suivant.

Merci!

PHQ-9

1) Date de complétion du questionnaire

____

Au cours des deux dernières semaines, à quelle fréquence avez-vous été dérangé par les problèmes suivants? Veuillez répondre à chacune des questions en encerclant l'énoncé qui correspond le mieux à votre situation.

		Jamais	Plusieurs jours	Plus de la moitié du temps	Presque tous les jours
2)	Peu d'intérêt ou de plaisir à faire les choses	◯	◯	◯	◯
3)	Vous sentir triste, déprimé ou désespéré	◯	◯	◯	◯
4)	Difficultés à vous endormir, à rester endormi ou trop dormir	◯	◯	◯	◯
5)	Vous sentir fatigué ou avoir peu d'énergie	◯	◯	◯	◯
6)	Peu d'appétit ou trop d'appétit	◯	◯	◯	◯
7)	Mauvaise perception de vous-même, vous pensez que vous êtes un perdant ou que vous n'avez pas satisfait vos propres attentes ou celles de votre famille	◯	◯	◯	◯
8)	Difficultés à vous concentrer sur des choses telles que lire le journal ou regarder la télévision	◯	◯	◯	◯
9)	Vous bougez ou vous parlez si lentement que les autres personnes ont pu le remarquer. Ou, au contraire, vous êtes si agité que vous bougez beaucoup plus que d'habitude	◯	◯	◯	◯
10)	Vous avez pensé que vous seriez mieux mort ou pensé à vous blesser d'une façon ou d'une autre	◯	◯	◯	◯

11) Si vous avez coché un problème parmi la liste ci-dessus, dans quelle mesure ces problèmes ont-ils rendu difficile pour vous de travailler, de vous occuper de vos tâches quotidiennes à la maison ou de vous entendre avec d'autres personnes?

◯ Pas du tout difficile ◯ Un peu difficile
◯ Très difficile ◯ Extrêmement difficile

Powered by REDCap

Examen physique

Identifiant : ______________________

Date examen : ______________________

Médecin :
- ◯ Sarah Maheux-Lacroix
- ◯ Madeleine Lemyre
- ◯ Philippe Laberge
- ◯ Annie-Kim Gareau-Labelle
- ◯ Kristina Arendas
- ◯ Jessica Lefebvre
- ◯ Marie-Ève Bergeron
- ◯ Autre

Si autre médecin, veuillez spécifier : ______________________

Consultation en fertilité? ◯ Oui ◯ Non

Examen Physique

Menstruations ? ◯ Oui ◯ Non

DDM ______________________

Raison aucune menstruation :
- ☐ Pas d'utérus
- ☐ Supprimée
- ☐ Aménorrhée
- ☐ Ménopause

Taille ______________________ (cm)

Poids ______________________ (kg)

IMC ______________________

TA systolique ______________________

TA diastolique ______________________

Fréquence cardiaque ______________________

Powered by REDCap

Douleur à la ceinture pelvienne :

☐ R. dorsal sacroiliac ligament
☐ L. dorsal sacroiliac ligament
☐ R. active straight leg raise
☐ L. active straight leg raise
☐ Symphisis pubis
☐ R. Faber
☐ L. Faber
☐ R. P4
☐ L. P4
☐ Non fait
☐ Négatif
(Cocher si présent)

Allodynie cutanée

☐ Droite ☐ Gauche ☐ Négatif
☐ Non fait

Abdomen

◯ Pas fait ◯ Pas sensible

	Sensibilité	Carnett - mieux	Carnett - équivalent	Carnett - pire-simple	Carnett - pire - multiple
Cadran inférieur DROIT	☐	☐	☐	☐	☐
Cadran inférieur GAUCHE	☐	☐	☐	☐	☐
Sus-pubien	☐	☐	☐	☐	☐
Cadran supérieur DROIT	☐	☐	☐	☐	☐
Cadran supérieur GAUCHE	☐	☐	☐	☐	☐
Autres (ex. cicatrices, etc.)	☐	☐	☐	☐	☐

Si autre, veuillez spécifier :

____

Présence de masse abdominale

◯ Oui ◯ Non

Masse abdominale

☐ Pas sensible ☐ Sensible

Taille masse abdominale

____
(mm (plus large))

Spéculum : Vulve / Vagin / Col

Examen Q-tip

◯ Fait ◯ Non fait

Vestibulodynie provoquée

◯ Oui

Spéculum

◯ Fait ◯ Non fait

Nodule vaginal

◯ Oui

Examen bimanuel

Utérus:	◯ Pas d'utérus<br>◯ Pas d'utérus/col de l'utérus
Douleur à la palpation de l'utérus :	◯ Oui ◯ Non
Utérus - taille :	◯ Normal ◯ Anormal
Nombre de semaines :	______________________<br>(semaines)
Utérus - mobilité	◯ Normale<br>◯ Réduite<br>◯ Fixe
Nodules :	◯ Oui ◯ Non
Combien de nodules?	______________________
#1 Nodule (taille) :	______________________<br>(cm)
#1 Nodule (emplacement) :	◯ Droite ◯ Gauche ◯ Centrale
#2 Nodule (taille) :	______________________<br>(cm)
#2 Nodule (emplacement) :	◯ Droite ◯ Gauche ◯ Centrale
#3 Nodule (taille) :	______________________<br>(cm)
#3 Nodule (emplacement) :	◯ Droite ◯ Gauche ◯ Centrale
#4 Nodule (taille) :	______________________<br>(cm)
#4 Nodule (emplacement) :	◯ Droite ◯ Gauche ◯ Centrale
#5 Nodule (taille) :	______________________<br>(cm)
#5 Nodule (emplacement) :	◯ Droite ◯ Gauche ◯ Centrale
Annexes :	◯ Normales ◯ Anormales<br>◯ Absentes

Annexes anormales :	☐ Masses annexielles ☐ Annexes fixes
Induration/Épaississement cul-de-sac postérieur	◯ Oui ◯ Non

Labo

AMH :	____________________
CA-125 :	____________________

Imagerie

Imagerie effectuée?	◯ Oui ◯ Non
Si oui :	☐ Échographie<br>☐ IRM
Échographie	____________________<br>(la plus récente)
Trouvailles à l'échographie :	☐ Kyste(s) à GAUCHE<br>☐ Kyste(s) à DROITE<br>☐ Nodule rectovaginal<br>☐ Atteinte digestive<br>☐ Atteinte vésicale<br>☐ Atteinte urétérale<br>☐ Anomalies utérines<br>☐ Autres<br>☐ Normale
IRM	____________________<br>(la plus récente)
Trouvailles à l'IRM :	☐ Kyste(s) à GAUCHE<br>☐ Kyste(s) à DROITE<br>☐ Nodule rectovaginal<br>☐ Atteinte digestive<br>☐ Atteinte vésicale<br>☐ Atteinte urétérale<br>☐ Anomalies utérines<br>☐ Autres<br>☐ Normale
Combien de kystes à GAUCHE?	____________________<br>(Max 3)
Kyste GAUCHE #1 - taille :	____________________<br>(cm)

Kyste GAUCHE #2 - taille :	______________ (cm)
Kyste GAUCHE #3 - taille :	______________ (cm)
Combien de kystes à DROITE?	______________ (Max 3)
Kyste DROITE #1 - taille :	______________ (cm)
Kyste DROITE #2 - taille :	______________ (cm)
Kyste DROITE #3 - taille :	______________ (cm)
Implication de l'uretère :	☐ Gauche ☐ Droite
Anomalies utérines :	☐ Fibromes<br>☐ Polypes<br>☐ Adénomyoses
Autre (spécifier) :	______________
Combien de kystes à GAUCHE?	______________ (Max 3)
Kyste GAUCHE #1 - taille :	______________ (cm)
Kyste GAUCHE #2 - taille :	______________ (cm)
Kyste GAUCHE #3 - taille :	______________ (cm)
Combien de kystes à DROITE?	______________ (Max 3)
Kyste DROITE #1 - taille :	______________ (cm)

Kyste DROITE #2 - taille :

____
(cm)

Kyste DROITE #3 - taille :

____
(cm)

Implication de l'uretère : ☐ Gauche ☐ Droite

Anomalies utérines :
- ☐ Fibromes
- ☐ Polypes
- ☐ Adénomyose

Autre (spécifier) :

____

Diagnostics

Endométriose
- ☐ Soupçonné
- ☐ Diagnostic visuel
- ☐ Endométriome
- ☐ Nodule
- ☐ Diagnostic par histologie
- ☐ Diagnostic par imagerie
- ☐ Laparoscopie NÉGATIVE
- ☐ NON soupçonné

Nombre de laparoscopies :

____

Nombre de laparoscopies confirmé par documentation :

____

Nombre de laparotomies :

____

Nombre de laparotomies confirmé par documentation :

____

Autres chirurgies dans le passé :
- ☐ Aucune
- ☐ Diagnostic l'endométriose
- ☐ Cautérisation de l'endométriose
- ☐ Excision de l'endométriose
- ☐ Cystectomie DROITE
- ☐ Cystectomie GAUCHE
- ☐ Ovariectomie DROITE
- ☐ Ovariectomie GAUCHE
- ☐ Hystérectomie
- ☐ Autre

Autre chirurgie :

____
(séparer avec une virgule)

Chirurgie pour diagnostic d'endométriose - année (plus récente) :

____

Diagnostic endométriose (vérifié) :	◯ Oui ◯ Non
Cautérisation endométriose - année (plus récente) :	______________________
Cautérisation endométriose (vérifié) :	◯ Oui ◯ Non
Excision de l'endométriose - année (plus récente) :	______________________
Excision de l'endométriose (vérifié) :	◯ Oui ◯ Non
Cystectomie droite - année (plus récente) :	______________________
Cystectomie droite (vérifié) :	◯ Oui ◯ Non
Cystectomie gauche- année (plus récente) :	______________________
Cystectomie gauche (vérifié) :	◯ Oui ◯ Non
Ovariectomie (droite) - année (plus récente) :	______________________
Ovariectomie droite (vérifié) :	◯ Oui ◯ Non
Ovariectomie (gauche) - année (plus récente) :	______________________
Ovariectomie gauche (vérifié) :	◯ Oui ◯ Non
Hystérectomie - année (plus récente) :	______________________
Hystérectomie (vérifié) :	◯ Oui ◯ Non
Autre chirurgie - année (plus récent) :	______________________
Autre chirurgie (vérifié) :	◯ Oui ◯ Non
Endométriose - Stade le plus élevé documenté :	◯ Stade 1/2<br>◯ Stade 3/4<br>◯ Inconnu<br>◯ Négatif (pas d'endométriose)
Utérus	☐ Adénomyose<br>☐ Fibromes<br>☐ Polypes<br>☐ Saignements anormaux

Saignements anormaux :	☐ Ménorragie<br>☐ Oligoménorrhée /aménorrhée<br>☐ Saignements irréguliers sous suppression hormonale/ Mirena<br>☐ Saignements intermenstruels
Pathologie ovaire :	☐ Kyste ovarien<br>☐ Ovaires restantes<br>☐ Adhérences<br>☐ HSX
Kyste ovarien	☐ Endométriome<br>☐ Kyste hémorragique<br>☐ Kyste simple<br>☐ Autre
Si autre kyste, veuillez spécifier :	________________________________
Douleur à la ceinture pelvienne	◯ Oui
Adhérences (non-ovarien)	◯ Oui
MSK	☐ Points gâchettes aux abdominaux (unique)<br>☐ Points gâchettes aux abdominaux (multiple)<br>☐ Douleur plancher pelvien/myalgie
Allodynie cutanée	◯ Oui
Masse/cicatrice douloureuse :	◯ Oui
Vessie douloureuse/Cystite interstitielle :	◯ Oui
Syndrome du côlon irritable	◯ Oui
Vulvodynie	☐ Vestibulodynie provoquée<br>☐ Vulvodynie<br>☐ Vaginisme
Neuropathie	☐ Ilioinguinal/Iliohypogastric<br>☐ Pudendal<br>☐ Other
Autres diagnostics :	________________________________
Comorbidités actuelles :	☐ Hx anxiété<br>☐ Hx dépression<br>☐ Sensibilisation centrale<br>☐ Usage quotidien de narcotiques<br>☐ Intolérance aux hormones<br>☐ Infertilité<br>☐ Pain Catastrophizing<br>☐ Autres
Autre comorbidité, veuillez l'indiquer :	________________________________

Powered by REDCap

Nombre d'années d'infertilité : ______________________

Échecs thérapeutiques antérieurs (persistance douleur ou intolérance) :

- ☐ COC
- ☐ Stérilet de lévonorgestrel
- ☐ Progestatif
- ☐ Agoniste de la GnRH
- ☐ Antagoniste de la GnRH
- ☐ Physiothérapie
- ☐ Psychothérapie
- ☐ Infiltrations
- ☐ Neuromodulateurs

Plan

Investigations :

- ☐ CA-125
- ☐ FSH/ E2
- ☐ AMH
- ☐ Échographie recherche
- ☐ Échographie
- ☐ IRM
- ☐ TDM
- ☐ Autre

Si autre investigation, veuillez spécifier : ______________________

Chirurgie prévue? ◯ Oui ◯ Non ◯ Non décidé

Type de chirurgie prévu :

- ☐ Excision de l'endometriose
- ☐ Cystectomie
- ☐ Ovariectomie
- ☐ Hystérectomie
- ☐ Présacral neurectomie
- ☐ Autre

Ovariectomie ◯ unilatérale ◯ bilatérale

Autre type de chirurgie prévu (spécifiez) ______________________

Médicaments/traitements PRESCRITS	☐ AINS<br>☐ Opioïdes<br>☐ COC<br>☐ Stérilet de levonorgestrel<br>☐ Progestatif<br>☐ Agoniste de la GnRH<br>☐ Antagoniste de la GnRH<br>☐ ____________________<br>☐ Marijuana<br>☐ Inhibiteur de l'aromatase<br>☐ Danazol<br>☐ SPRM<br>☐ Anti-épileptiques<br>☐ Anti-depresseurs<br>☐ Autres neuromodulateurs<br>☐ Relaxants musculaires<br>☐ Crèmes topiques<br>☐ Injections point gachette<br>☐ Autre<br>☐ Autre intervenant
Spécifier type d'opioïdes :	☐ Hydromorphone (dilaudid)<br>☐ Morphine<br>☐ Oxycodone<br>☐ Tramadol<br>☐ Fentanyl<br>☐ Methadone<br>☐ Codeine<br>☐ Autre
Si autre type d'opioïdes, veuillez spécifier :	____________________
Spécifier type COC	◯ Continu<br>◯ Cyclique
COC :	◯ Alesse<br>◯ Yasmin<br>◯ Yaz<br>◯ Marvelon<br>◯ Brevicon<br>◯ Cyclen<br>◯ MinOvral<br>◯ Seasonale/ Seasonique<br>◯ Evra patch<br>◯ NuvaRing<br>◯ LoLo<br>◯ Other
Spécifier progestatif :	☐ Provera ☐ Visanne<br>☐ Norlutate ☐ Micronor
Provera - spécifier la dose :	____________________<br>(mg)
Visanne - spécifier la dose :	____________________<br>(mg)

Norlutate - spécifier la dose :

______________________ (mg)

Micronor - spécifier la dose :

______________________ (mg)

IUD

☐ Mirena ☐ Kyleena
☐ Jaydess

Danazol :

☐ Oral ☐ Vaginal

Agoniste de la GNRH :

☐ Lupron 11.25 mg IM q 3 mois
☐ Lupron 3.75 mg IM q 1 mois
☐ Synarel
☐ Autre

Si autre agoniste de la GnRH, veuillez spécifier :

____

Antagoniste de la GnRH :

☐ Elagolix (orlissa) 150 mg PO die
☐ Elagolix (orlissa) 200 mg PO BID

SPRM :

☐ Fibristal
☐ Autre

Si autre SPRM, veuillez spécifier :

____

Inhibiteur de l'aromatase :

☐ Letrozole
☐ Autre

Si autre inhibiteur de l'aromatase, veuillez spécifier :

____

Anti-épileptique :

☐ Gabapentin
☐ Pregabalin

Anti-dépresseurs :

☐ Nortriptyline
☐ Desipramine
☐ Cymbalta
☐ Autre

Si autre anti-dépresseurs, veuillez spécifier :

____

Autres neuromodulateurs :

☐ Nozinan
☐ Cesamet
☐ Autre

Si autres neuromodulateurs, veuillez spécifier :

____

Relaxant musculaire :	☐ Baclofen<br>☐ Cyclobenzaprine<br>☐ Vaginal diazepam
Crème topique :	☐ Lidocaine ☐ Gabapentin<br>☐ Autre
Si autre médication, veuillez spécifier :	______________________
Veuillez spécifier les autres intervenants :	☐ Urologie<br>☐ Gen Sx<br>☐ Radiologie (embolisation)<br>☐ Autre<br>☐ Autre clinique pour la douleur
Si autre, veuillez spécifier :	______________________
Multidisciplinaire	☐ Psychologue<br>☐ Physiothérapie
Plan suivi :	☐ Après chirurgie<br>☐ Après physiothérapie et/ou psychothérapie<br>☐ 1-3 mois<br>☐ 4-6 mois<br>☐ 7-12 mois<br>☐ Libérée<br>☐ Non décidé

Commentaires

Echographie

Identifiant : ______________________

Échographie

Date de l'examen : ______________________

Préparation intestinale ◯ Oui ◯ Non

Suppression hormonale ◯ Oui ◯ Non

Jour du cycle ______________________

Précisez :
- ☐ Inhibiteur de l'aromatase
- ☐ COC
- ☐ Progestatif
- ☐ Stérilet de levonorgestrel
- ☐ Stériel de cuivre
- ☐ Danazol
- ☐ Agoniste de la GnRH
- ☐ Antagoniste de la GnRH
- ☐ SPRM

Utérus

Utérus ◯ Normal ◯ Anormal ◯ Absent

Taille de l'utérus - LARGEUR ______________________ (mm)

Taille de l'utérus - LONGUEUR ______________________ (mm)

Taille de l'utérus - HAUTEUR ______________________ (mm)

Orientation

◯ Antéfléchi ◯ Antéversé ◯ Intermédiaire ◯ Rétroversé ◯ Rétrofléchi

Jour du cycle ______________________

Phase du cycle ◯ Menstruations ◯ Folliculaire ◯ Lutéale

Polype ◯ Oui ◯ Non

Taille du polype - LARGEUR

____
((mm))

Taille du polype - LONGUEUR

____
((mm))

Taille du polype - HAUTEUR

____
((mm))

Nombre de fibromes :

◯ Aucun ◯ 1 fibrome ◯ 2 fibromes ◯ 3 fibromes ◯ Plus de 3 fibromes

Fibrome 1

☐ Sous-muqueux ☐ Intra-mural ☐ Sous-séreux

Fibrome 1 - localisation

◯ Fundique ◯ Antérieur ◯ Postérieur ◯ Latéral gauche ◯ Latéral droit ◯ Cervical

Fibrome 1 - taille - LARGEUR

____
((mm))

Fibrome 1 - taille - LONGUEUR

____
((mm))

Fibrome 1 - taille - HAUTEUR

____
((mm))

Fibrome 2

☐ Sous-muqueux ☐ Intra-mural ☐ Sous-séreux

Fibrome 2 - localisation

◯ Fundique ◯ Antérieur ◯ Postérieur ◯ Latéral gauche ◯ Latéral droit ◯ Cervical

Fibrome 2 - taille - LARGEUR

____
((mm))

Fibrome 2 - taille - LONGUEUR

____
((mm))

Fibrome 2 - taille - HAUTEUR

____
((mm))

Fibrome 3

☐ Sous-muqueux ☐ Intra-mural ☐ Sous-séreux

Fibrome 3 - localisation

◯ Fundique ◯ Antérieur ◯ Postérieur ◯ Latéral gauche ◯ Latéral droit ◯ Cervical

Fibrome 3 - taille - LARGEUR

____
((mm))

Fibrome 3 - taille - LONGUEUR

____
((mm))

Fibrome 3 - taille - HAUTEUR

____
((mm))

Adénomyose - Nombre de lésions

◯ Non ◯ 1 lésion ◯ 2 lésions ◯ 3 lésions ◯ Plus de 3 lésions

Adénomyose - Lésion 1 - Localisation

☐ Fundique ☐ Antérieur ☐ Postérieur ☐ Latéral gauche ☐ Latéral droit ☐ Cervical

Adénomyose - Lésion 1 - Différenciation

◯ Focal ◯ Diffus ◯ Mixte

Adénomyose - Lésion 1 - Kystique

◯ Oui ◯ Non

Adénomyose - Lésion 1 - Couche utérine affectée

☐ Zone jonctionnelle (interne) ☐ Myomètre moyen ☐ Sous-séreux (externe)

Adénomyose - Lésion 1 - Taille - LARGEUR

____
((mm))

Adénomyose - Lésion 1 - Taille - LONGUEUR

____
((mm))

Adénomyose - Lésion 1 - Taille - HAUTEUR

____
((mm))

Adénomyose - Lésion 2 - Localisation

☐ Fundique ☐ Antérieur ☐ Postérieur ☐ Latéral gauche ☐ Latéral droit ☐ Cervical

Adénomyose - Lésion 2 - Différenciation

◯ Focal ◯ Diffus ◯ Mixte

Adénomyose - Lésion 2 - Kystique

◯ Oui ◯ Non

Adénomyose - Lésion 2 - Couche utérine affectée

☐ Zone jonctionnelle (interne) ☐ Myomètre moyen ☐ Sous-séreux (externe)

Adénomyose - Lésion 2 - Taille - LARGEUR

____
((mm))

Adénomyose - Lésion 2 - Taille - LONGUEUR

____
((mm))

Adénomyose - Lésion 2 - Taille - HAUTEUR

____
((mm))

Adénomyose - Lésion 3 - Localisation

☐ Fundique ☐ Antérieur ☐ Postérieur ☐ Latéral gauche ☐ Latéral droit ☐ Cervical

Adénomyose - Lésion 3 - Différenciation

◯ Focal ◯ Diffus ◯ Mixte

Adénomyose - Lésion 3 - Kystique

◯ Oui ◯ Non

Adénomyose - Lésion 3 - Couche utérine affectée

☐ Zone jonctionnelle (interne) ☐ Myomètre moyen ☐ Sous-séreux (externe)

Adénomyose - Lésion 3 - Taille - LARGEUR

____
((mm))

Adénomyose - Lésion 3 - Taille - LONGUEUR

____
((mm))

Adénomyose - Lésion 3 - Taille - HAUTEUR

____
((mm))

Extension (proportion du corps utérin étant affecté par l'adénomyose)

◯ Légère (< 25%) ◯ Modérée (25-50%) ◯ Sévère (>50%)

Isthmocèle ◯ Oui ◯ Non

Isthmocèle - taille - LARGEUR

_______________________ ((mm))

Isthmocèle - taille - LONGUEUR

_______________________ ((mm))

Isthmocèle - taille - HAUTEUR

_______________________ ((mm))

Épaisseur du myomètre résiduel

_______________________ ((mm))

Distance de l'os interne

_______________________ ((mm))

Mobilité utérine ◯ Normale ◯ Réduite ◯ Fixe

Compartiment antérieur

Compartiment antérieur - Nombre de lésions d'endométriose

◯ Non ◯ 1 lésion ◯ 2 lésions ◯ 3 lésions ◯ Plus de 3 lésions

Compartiment antérieur- Lésion 1 - Localisation

◯ Vessie ◯ Péritoine vésico-utérin ◯ Ligament rond droit ◯ Ligament rond gauche

Compartiment antérieur - Lésion 1 - Taille - LARGEUR

_______________________ ((mm))

Compartiment antérieur - Lésion 1 - Taille - LONGUEUR

_______________________ ((mm))

Compartiment antérieur - Lésion 1 - Taille - HAUTEUR

_______________________ ((mm))

Compartiment antérieur- Lésion 2 - Localisation

◯ Vessie ◯ Péritoine vésico-utérin ◯ Ligament rond droit ◯ Ligament rond gauche

Compartiment antérieur - Lésion 2 - Taille - LARGEUR

____
((mm))

Compartiment antérieur - Lésion 2 - Taille - LONGUEUR

____
((mm))

Compartiment antérieur - Lésion 2 - Taille - HAUTEUR

____
((mm))

Compartiment antérieur- Lésion 3 - Localisation

◯ Vessie ◯ Péritoine vésico-utérin ◯ Ligament rond droit ◯ Ligament rond gauche

Compartiment antérieur - Lésion 3 - Taille - LARGEUR

____
((mm))

Compartiment antérieur - Lésion 3 - Taille - LONGUEUR

____
((mm))

Compartiment antérieur - Lésion 3 - Taille - HAUTEUR

____
((mm))

Sliding sign vessie-utérus

◯ Positif (glissement)
◯ Négatif (absence de glissement)

Compartiment du milieu

Annexe GAUCHE

Annexe GAUCHE :

◯ Normale ◯ Anormale ◯ Non visualisée ◯ Absente

Taille de l'annexe gauche - LARGEUR

____
((mm))

Taille de l'annexe gauche - LONGUEUR

____
((mm))

Taille de l'annexe gauche - HAUTEUR

____
((mm))

Annexe gauche - Nombre de lésions

◯ Non ◯ 1 lésion ◯ 2 lésions ◯ 3 lésions ◯ Plus de 3 lésions

Annexe gauche - Lésion 1 - Type kyste

☐ Séreux ☐ Hémorragique ☐ Endométriome ☐ Complexe ☐ Hydrosalpinx ☐ Hématosalpinx
☐ Follicule

Annexe gauche - Lésion 1 - Taille - LARGEUR

____
((mm))

Annexe gauche - Lésion 1 - Taille - LONGUEUR

____
((mm))

Annexe gauche - Lésion 1 - Taille - HAUTEUR

____
((mm))

Annexe gauche - Lésion 2 - Type kyste

☐ Séreux ☐ Hémorragique ☐ Endométriome ☐ Complexe ☐ Hydrosalpinx ☐ Hématosalpinx
☐ Follicule

Annexe gauche - Lésion 2 - Taille - LARGEUR

____
((mm))

Annexe gauche - Lésion 2 - Taille - LONGUEUR

____
((mm))

Annexe gauche - Lésion 2 - Taille - HAUTEUR

____
((mm))

Annexe gauche - Lésion 3 - Type kyste

☐ Séreux ☐ Hémorragique ☐ Endométriome ☐ Complexe ☐ Hydrosalpinx ☐ Hématosalpinx
☐ Follicule

Annexe gauche - Lésion 3 - Taille - LARGEUR

____
((mm))

Annexe gauche - Lésion 3 - Taille - LONGUEUR

____
((mm))

Annexe gauche - Lésion 3 - Taille - HAUTEUR

____
((mm))

Ovaire gauche - Sliding sign

◯ Positif (glissement)
◯ Négatif (absence de glissement)

Ovaire gauche - Nombre de follicules antraux ◯ Non évalué

Ovaire gauche - Nombre de follicules antraux ____________________

Lésion uretère gauche

◯ Oui ◯ Non ◯ Non visualisée ◯ Non évaluée

Lésion uretère gauche-Distance de la jonction vésico-urétérale ____________________ ((mm))

Lésion uretère gauche - commentaires (taille et % de circonférence) ____________________

Hydronéphrose gauche

◯ Non ◯ Légère ◯ Modérée ◯ Sévère ◯ Non-évaluée

Annexe DROITE

Annexe DROITE

◯ Normale ◯ Anormale ◯ Non visualisée ◯ Absente

Taille de l'annexe droite - LARGEUR ____________________ ((mm))

Taille de l'annexe droite - LONGUEUR ____________________ ((mm))

Taille de l'annexe droite - HAUTEUR ____________________ ((mm))

Annexe droite- Nombre de lésions

◯ Non ◯ 1 lésion ◯ 2 lésions ◯ 3 lésions ◯ Plus de 3 lésions

Annexe droite- Lésion 1 - Type kyste

☐ Séreux ☐ Hémorragique ☐ Endométriome ☐ Complexe ☐ Hydrosalpinx ☐ Hématosalpinx
☐ Follicule

Annexe droite- Lésion 1 - Taille - LARGEUR ____________________ ((mm))

Annexe droite- Lésion 1 - Taille - LONGUEUR ____________________ ((mm))

Powered by REDCap

Annexe droite- Lésion 1 - Taille - HAUTEUR

____
((mm))

Annexe droite- Lésion 2 - Type kyste

☐ Séreux ☐ Hémorragique ☐ Endométriome ☐ Complexe ☐ Hydrosalpinx ☐ Hématosalpinx
☐ Follicule

Annexe droite- Lésion 2 - Taille - LARGEUR

____
((mm))

Annexe droite- Lésion 2 - Taille - LONGUEUR

____
((mm))

Annexe droite- Lésion 2 - Taille - HAUTEUR

____
((mm))

Annexe droite- Lésion 3 - Type kyste

☐ Séreux ☐ Hémorragique ☐ Endométriome ☐ Complexe ☐ Hydrosalpinx ☐ Hématosalpinx
☐ Follicule

Annexe droite- Lésion 3 - Taille

____
(indiquer : profondeur; largeur; hauteur (mm))

Annexe droite- Lésion 3 - Taille - LARGEUR

____
((mm))

Annexe droite- Lésion 3 - Taille - LONGUEUR

____
((mm))

Annexe droite- Lésion 3 - Taille - HAUTEUR

____
((mm))

Ovaire droite - Sliding sign

◯ Positif (glissement)
◯ Négatif (absence de glissement)

Ovaire droite- Nombre de follicules antraux

◯ Non évalué

Ovaire droite - Nombre de follicules antraux

____

Lésion uretère droite

◯ Oui ◯ Non ◯ Non visualisée ◯ Non évaluée

Lésion uretère droite - Distance de la jonction vésico-urétérale

____
((mm))

Lésion uretère droite- commentaires
(taille et % de circonférence)

____

Hydronéphrose droite

◯ Non ◯ Légère ◯ Modérée ◯ Sévère ◯ Non-évaluée

Décompte follicules antraux total

____

Compartiment postérieur

Compartiment postérieur - Nombre de lésions d'endométriose

◯ Non ◯ 1 lésion ◯ 2 lésions ◯ 3 lésions ◯ Plus de 3 lésions

Compartiment postérieur- Lésion 1 - Localisation

☐ Ligament utéro-sacré droit
☐ Ligament utéro-sacré gauche
☐ Torus utérin (espace rétro-cervical)
☐ Cul-de-sac de Dougals
☐ Vagin
☐ Recto-sigmoïde

Compartiment postérieur- Lésion 1 - Taille - LARGEUR

____
((mm))

Compartiment postérieur- Lésion 1 - Taille - LONGUEUR

____
((mm))

Compartiment postérieur- Lésion 1 - Taille - HAUTEUR

____
((mm))

Compartiment postérieur - Lésion 1 - Distance lésion-marge anale

____

Lésion 1 rectosigmoïde - commentaire
(profondeur de l'invasion [séreuse, musculeuse, trans-rectale] et % circonférence affectée)

____

Compartiment postérieur- Lésion 2 - Localisation

☐ Ligament utéro-sacré droit
☐ Ligament utéro-sacré gauche
☐ Torus utérin (espace rétro-cervical)
☐ Cul-de-sac de Dougals
☐ Vagin
☐ Recto-sigmoïde

Compartiment postérieur- Lésion 2 - Taille - LARGEUR

____
((mm))

Compartiment postérieur- Lésion 2 - Taille - LONGUEUR

____
((mm))

Compartiment postérieur- Lésion 2 - Taille - HAUTEUR

____
((mm))

Compartiment postérieur - Lésion 2 - Distance lésion-marge anale

____

Lésion 2 rectosigmoïde - commentaire (profondeur de l'invasion [séreuse, musculeuse, trans-rectale] et % circonférence affectée)

____

Compartiment postérieur- Lésion 3 - Localisation

☐ Ligament utéro-sacré droit
☐ Ligament utéro-sacré gauche
☐ Torus utérin (espace rétro-cervical)
☐ Cul-de-sac de Dougals
☐ Vagin
☐ Recto-sigmoïde

Compartiment postérieur- Lésion 3 - Taille - LARGEUR

____
((mm))

Compartiment postérieur- Lésion 3 - Taille - LONGUEUR

____
((mm))

Compartiment postérieur- Lésion 3 - Taille - HAUTEUR

____
((mm))

Compartiment postérieur - Lésion 3 - Distance lésion-marge anale

____

Lésion 3 rectosigmoïde - commentaire (profondeur de l'invasion [séreuse, musculeuse, trans-rectale] et % circonférence affectée)

____

Sliding sign postérieur

○ Positif (glissement)
○ Négatif (absence de glissement)

Autres sites

Autres sites - lésion d'endométriose

○ Aucune lésion ○ 1 lésion ○ 2 lésions ○ 3 lésions ○ Plus de 3 lésions

Autres sites - localisation

☐ Diaphragme ☐ Ombilic ☐ Cicatrice de césarienne ☐ Appendice ☐ Iléon terminal
☐ Caecum ☐ Autre

Autres sites - si autre localisation, veuillez précisez :

____

Diaphragme - Lésion - Taille - LARGEUR

____
((mm))

Diaphragme - Lésion - Taille - LONGUEUR

____
((mm))

Diaphragme - Lésion - Taille - HAUTEUR

____
((mm))

Ombilic - Lésion - Taille - LARGEUR

____
((mm))

Ombilic - Lésion - Taille - LONGUEUR

____
((mm))

Ombilic - Lésion - Taille - HAUTEUR

____
((mm))

Cicatrice de césarienne - Lésion - Taille - LARGEUR

____
((mm))

Cicatrice de césarienne - Lésion - Taille - LONGUEUR

____
((mm))

Cicatrice de césarienne - Lésion - Taille - HAUTEUR

____
((mm))

Appendice - Lésion - Taille - LARGEUR

____
((mm))

Appendice - Lésion - Taille - LONGUEUR

____
((mm))

Appendice - Lésion - Taille - HAUTEUR

____
((mm))

Iléon terminal - Lésion - Taille - LARGEUR

____
((mm))

Iléon terminal - Lésion - Taille - LONGUEUR

____
((mm))

Iléon terminal - Lésion - Taille - HAUTEUR

____
((mm))

Caecum - Lésion - Taille - LARGEUR

____
((mm))

Caecum - Lésion - Taille - LONGUEUR

____
((mm))

Caecum - Lésion - Taille - HAUTEUR

____
((mm))

Autre - Lésion - Taille - LARGEUR

____
((mm))

Autre - Lésion - Taille - LONGUEUR

____
((mm))

Autre - Lésion - Taille - HAUTEUR

____
((mm))

Sliding sign nombril

◯ Positif (glissement)
◯ Négatif (absence de glissement)

Échographie cartographie de la douleur

◯ Pas fait ◯ Pas sensible/douloureux

	Vessie	Col de l'utérus	Utérus	Voûte droite	Voûte centrale	Voûte gauche
Sensibilité/douleur	☐	☐	☐	☐	☐	☐

	Cul-de-sac	Lig. US droit	Lig. US gauche	Annexe (DROITE)	Annexe (GAUCHE)
Sensibilité/douleur	☐	☐	☐	☐	☐

Conclusion

Commentaires / Conclusion

____

Surgical Database

Identifiant : ______________________

Surgeon ______________________

Date of Surgery ______________________

I. Menses

LMP ______________________

Cycle day ______________________

Currently bleeding? ◯ Yes ◯ No

II. Current Hormonal Treatment

Suppression hormonale en pré-opératoire? ◯ Oui ◯ Non

Si oui, qu'avez-vous prescrit ?
- ☐ Inhibiteur de l'aromatase
- ☐ COC
- ☐ Progestatif
- ☐ Stérilet de levonorgestrel
- ☐ Danazol
- ☐ Agoniste de la GnRH
- ☐ Antagoniste de la GnRH
- ☐ SPRM

Inhibiteur de l'aromatase : ☐ Letrozole ☐ Autre

Si autre inhibiteur de l'aromatase, veuillez spécifier : ______________________

Spécifier type COC :
- ◯ Continu
- ◯ Cyclique

COC :
◯ Alesse ◯ Yasmin ◯ Yaz
◯ Marvelon ◯ Brevicon
◯ Cyclen ◯ MinOvral
◯ Seasonale/ Seasonique
◯ Evra patch ◯ NuvaRing
◯ LoLo ◯ Other

Spécifier progestatif :
☐ Provera ☐ Visanne
☐ Norlutate ☐ Micronor

Provera - spécifier la dose : ______________________ (mg)

Visanne - spécifier la dose : ______________________ (mg)

Norlutate - spécifier la dose : ______________________ (mg)

Micronor - spécifier la dose : ______________________ (mg)

IUD
☐ Mirena ☐ Kyleena
☐ Jaydess

Danazol : ☐ Oral ☐ Vaginal

Agoniste de la GnRH :
☐ Lupron 11.25 mg IM q 3 mois
☐ Lupron 3.75 mg IM q 1 mois
☐ Synarel
☐ Autre

Si autre agoniste de la GnRH, veuillez spécifier : ______________________

Antagoniste de la GnRH :
◯ Elagolix (orlissa) 150 mg PO die
◯ Elagolix (orlissa) 200 mg PO BID

SPRM : ☐ Fibristal ☐ Autre

Si autre SPRM, veuillez spécifier : ______________________

Durée de la suppression hormonale (en mois) : ______________________ (mois)

Last application (dose): ______________________

III. Previous surgical diagnosis of endometriosis

Previous surgical diagnosis of endometriosis ◯ Yes ◯ No ◯ Do not know

How many previous surgical diagnoses of endometriosis? ______________________ (Max 3)

1) First Surgery - Hospital? ______________________

1) First Surgery - When? ______________________

1) First Surgery - Procedure(s)? ______________________

2) Second Surgery - Hospital?

____

2) Second Surgery - When?

____

2) Second Surgery - Procedure(s)?

____

3) Third Surgery - Hospital?

____

3) Third Surgery - When?

____

3) Third Surgery - Procedure(s)?

____

IV. Imaging prior to surgery

Imaging prior to surgery? ◯ Yes ◯ No

If yes:
- ☐ Ultrasound
- ☐ MRI

Ultrasound

____

MRI

____

Trouvailles à l'échographie :
- ☐ Kyste(s) à GAUCHE
- ☐ Kyste(s) à DROITE
- ☐ Nodule rectovaginal
- ☐ Atteinte digestive
- ☐ Atteinte vésicale
- ☐ Atteinte urétérale
- ☐ Anomalies utérines
- ☐ Autres
- ☐ Normale

Trouvailles à l'IRM :
- ☐ Kyste(s) à GAUCHE
- ☐ Kyste(s) à DROITE
- ☐ Nodule rectovaginal
- ☐ Atteinte digestive
- ☐ Atteinte vésicale
- ☐ Atteinte urétérale
- ☐ Anomalies utérines
- ☐ Autres
- ☐ Normale

Left cyst #1, size:

____
(cm)

Left cyst, how many?

________________ (Max 3)

Left cyst #2, size:

________________ (cm)

Left cyst #3, size:

________________ (cm)

Right cyst, how many?

________________ (Max 3)

Right cyst #1, size:

________________ (cm)

Right cyst #2, size:

________________ (cm)

Right cyst #3, size:

________________ (cm)

Ureter involvement: ☐ Left ☐ Right

Uterine anomalies:
☐ Fibroids
☐ Polyps
☐ Adenomyosis

Other (specify):

____

Left cyst, how many?

________________ (Max 3)

Left cyst #1, size:

________________ (cm)

Left cyst #2, size:

________________ (cm)

Left cyst #3, size:

________________ (cm)

Right cyst, how many?

________________ (Max 3)

Right cyst #1, size:

____
(cm)

Right cyst #2, size:

____
(cm)

Right cyst #3, size:

____
(cm)

Ureter involvement: ☐ Left ☐ Right

Uterine anomalies:
☐ Fibroids
☐ Polyps
☐ Adenomyosis

Other (specify):

____

V. Procedures

Total surgical time

____
(minutes)

Approach ☐ Laparoscopy ☐ Laparotomy

Uterine cavity surgery: ◯ Yes ◯ No

Hysteroscopy:

☐ Before laparoscopy ☐ After laparoscopy

Findings

☐ Normal ☐ Abnormal

Abnormal

Hysteroscopy

☐ Diagnostic hysteroscopy ☐ Polypectomy ☐ Resection of fibroid ☐ Resection of endometrium
☐ Resection of septum/adhesion ☐ Other

Other

____

Ovarian surgery: ◯ Yes ◯ No

Ovarian surgery:	☐ Surface (excision, ablation, fulguration, ovariolysis, suspension)<br>☐ Oophorectomy<br>☐ Cystectomy<br>☐ Reconstruction<br>☐ Cyst aspiration/drainage<br>☐ Cyst ablation
Ovarian surgery: surface	☐ Excision<br>☐ Ablation<br>☐ Fulguration<br>☐ Ovariolysis<br>☐ Temporary suspension
Excision:	◯ Left ◯ Right ◯ Both
Excision:	☐ Laser<br>☐ Monopolar<br>☐ Bipolar<br>☐ Plasmajet
Ablation:	◯ Left ◯ Right ◯ Both
Ablation:	☐ Laser<br>☐ Monopolar<br>☐ Bipolar<br>☐ Plasmajet
Fulguration:	◯ Left ◯ Right ◯ Both
Ovariolysis:	◯ Left ◯ Right ◯ Both
Temporary suspension:	◯ Left ◯ Right ◯ Both
Oophorectomy:	◯ Left ◯ Right ◯ Both
Ovarian cystectomy:	◯ Left ◯ Right ◯ Both
Ovarian reconstruction:	◯ Left ◯ Right ◯ Both
Cyst aspiration/drainage:	◯ Left ◯ Right ◯ Both
Cyst ablation:	◯ Left ◯ Right ◯ Both
Tubal surgery:	◯ Yes ◯ No
Tubal surgery:	☐ Fimbrioplasty<br>☐ Tuboplasty<br>☐ Lysis of adhesions (salpingolysis)<br>☐ Salpingectomy
Fimbrioplasty:	◯ Left ◯ Right ◯ Both

Tuboplasty:	◯ Left ◯ Right ◯ Both
Lysis of adhesions (salpingolysis):	◯ Left ◯ Right ◯ Both
Salpingectomy:	◯ Left ◯ Right ◯ Both
Peritoneal surgery:	◯ Yes ◯ No
Peritoneal surgery:	☐ Destruction of endometriosis<br>☐ Excision of endometriosis<br>☐ Other
Peritoneum: destruction of endometriosis	☐ Electrosurgery (monopolar)<br>☐ Electrosurgery (bipolar)<br>☐ Laser<br>☐ Other
Destruction of endometriosis, laser type:	__________________________
Destruction of endometriosis, other:	__________________________
Peritoneum: excision of endometriosis	☐ Electrosurgery (monopolar)<br>☐ Electrosurgery (bipolar)<br>☐ Laser<br>☐ Scissors<br>☐ Harmonic scalpel<br>☐ Other
Excision of endometriosis, laser type:	__________________________
Excision of endometriosis, other:	__________________________
Peritoneal surgery, number of specimens:	__________________________
Peritoneal surgery, other:	__________________________
Peritoneal fluid volume:	__________________________<br>(ml)
Peritoneal fluid:	☐ Clear<br>☐ Bloody
Bladder surgery:	◯ Yes ◯ No
Bladder surgery: viscera entered?	◯ Yes ◯ No

Bladder surgery, specify:	______________________
Ureter surgery:	◯ Yes ◯ No
Ureter surgery:	☐ Left ☐ Right
Left ureterolysis: mucosa entered?	◯ Yes ◯ No
Mucosa entered, specify:	______________________
Left ureterolysis	☐ Primary repair<br>☐ Segmental resection<br>☐ Psoas hitch
Psoas hitch, specify:	______________________
Right ureterolysis: mucosa entered?	◯ Yes ◯ No
Mucosa entered, specify:	______________________
Right ureterolysis	☐ Primary repair<br>☐ Segmental resection<br>☐ Psoas hitch
Psoas hitch, specify:	______________________
Bowel surgery:	◯ Yes ◯ No
Bowel surgery, mucosa entered?	◯ Yes ◯ No
Mucosa entered, specify:	______________________
Bowel surgery	☐ Nodule removed<br>☐ Discectomy<br>☐ Bowel resection<br>☐ Appendectomy<br>☐ Other
Bowel surgery, other:	______________________
Uterine surgery:	◯ Yes ◯ No
Uterine surgery:	◯ Hysterectomy<br>◯ Myomectomy

Hysterectomy	◯ Total<br>◯ Subtotal<br>◯ LAVH<br>◯ Other
Hysterectomy, other:	______________________
Other procedures:	______________________

VI. At conclusion of surgery

Residual peritoneal endometriosis?	◯ Yes ◯ No
Location(s):	______________________
Residual adhesions?	◯ Yes ◯ No
Location(s):	______________________
Residual endometriomas?	◯ Yes ◯ No
Location(s):	______________________
Residual nodules?	◯ Yes ◯ No
Location(s):	______________________

VII. Intraoperative complications

Intraoperative complications?	◯ Yes ◯ No
Type(s):	______________________
Treatment(s):	______________________

VIII. Pathology during surgery

Any pathology observed during surgery?	◯ Yes ◯ No
Visual diagnosis of endometriosis?	◯ Yes ◯ No
Specify which type seen:	☐ Peritoneal<br>☐ Ovarian<br>☐ Deeply infiltrative

Revised American Fertility Society Score Mark the total area of endometriosis possibly of multiple lesions NOT just the largest lesion."

Endometriosis - Peritoneum

Superficial:	◯ < 1 cm ◯ 1-3 cm ◯ >3 cm<br>◯ N/A<br>(MUST tick N/A if does not apply for calculation to function)
Deep:	◯ < 1 cm ◯ 1-3 cm ◯ >3 cm<br>◯ N/A<br>(MUST tick N/A if none apply for calculation to function)

Endometriosis - Ovary - LEFT

Superficial:	◯ < 1 cm ◯ 1-3 cm ◯ >3 cm<br>◯ N/A<br>(MUST tick N/A if none apply for calculation to function)
Deep:	◯ < 1 cm ◯ 1-3 cm ◯ >3 cm<br>◯ N/A<br>(MUST tick N/A if none apply for calculation to function)

Endometriosis - Ovary - RIGHT

Superficial:	◯ < 1 cm ◯ 1-3 cm ◯ >3 cm<br>◯ N/A<br>(MUST click N/A if none apply for calculation)
Deep:	◯ < 1 cm ◯ 1-3 cm ◯ >3 cm<br>◯ N/A<br>(MUST tick N/A if none apply for calculation to function)
Pouch of Douglas obliteration	◯ Partial ◯ Complete<br>◯ N/A
AFS endometriosis score:	__________________________________

Adhesions - LEFT ovary For all dense adhesions, if the fimbriated end of the fallopian tube is completely enclosed, please indicate >2/3 enclosure"

Filmy	◯ < 1/3 enclosure ◯ 1/3 - 2/3<br>◯ >2/3 enclosure ◯ N/A<br>(MUST tick N/A if none apply for calculation to function)
Dense	◯ < 1/3 enclosure ◯ 1/3 - 2/3<br>◯ >2/3 enclosure ◯ N/A<br>(MUST tick N/A if none apply for calculation to function)
Adhesions - RIGHT ovary	
Filmy	◯ < 1/3 enclosure ◯ 1/3 - 2/3<br>◯ >2/3 enclosure ◯ N/A<br>(MUST tick N/A if none apply for calculation to function)
Dense	◯ < 1/3 enclosure ◯ 1/3 - 2/3<br>◯ >2/3 enclosure ◯ N/A<br>(MUST tick N/A if none apply for calculation to function)
Adhesions - LEFT tube	
Filmy	◯ < 1/3 enclosure ◯ 1/3 - 2/3<br>◯ >2/3 enclosure ◯ N/A<br>(MUST tick N/A if none apply for calculation to function)
Dense	◯ < 1/3 enclosure ◯ 1/3 - 2/3<br>◯ >2/3 enclosure ◯ N/A<br>(MUST tick N/A if none apply for calculation to function)
Adhesions - RIGHT tube	
Filmy	◯ < 1/3 enclosure ◯ 1/3 - 2/3<br>◯ >2/3 enclosure ◯ N/A<br>(MUST tick N/A if none apply for calculation to function)
Dense	◯ < 1/3 enclosure ◯ 1/3 - 2/3<br>◯ >2/3 enclosure ◯ N/A<br>(MUST tick N/A if none apply for calculation to function)
AFS adhesion score:	________________________
AFS total score:	________________________
STAGE I (Minimal): 1-5	
STAGE II (Mild): 6-15	
STAGE III (Moderate): 16-40	

STAGE IV (Severe): >40

Deeply infiltrative endometriosis (DIE) ◯ Yes ◯ No

DIE Location
- ☐ Pelvic sidewall
- ☐ Ureter
- ☐ Posterior cul-de-sac (Pouch of Douglas)
- ☐ Rectum
- ☐ Sigmoid
- ☐ Bladder
- ☐ Parametrium
- ☐ Uterosacral ligament
- ☐ Vagina
- ☐ Other

DIE Pelvic side wall: ☐ Left ☐ Right

DIE Ureter: ☐ Left ☐ Right

DIE Uterosacral ligament: ☐ Left ☐ Right

Other ____________________

IX. Location of endometriosis, number and appearance of lesions:

[Attachment: "EPHect example pictures.docx"]

Location of endometriosis LEFT

	Vascular	Clear	Yellow	Red	White	Blue/Black	Brown	Filmy adhesions	Web adhesions	Thin adhesions	Dense adhesions	Sac like adhesions
Left pelvic sidewall	☐	☐	☐	☐	☐	☐	☐	☐	☐	☐	☐	☐
Left uterosacral ligament	☐	☐	☐	☐	☐	☐	☐	☐	☐	☐	☐	☐
Left ovary - serosa	☐	☐	☐	☐	☐	☐	☐	☐	☐	☐	☐	☐
Left tube - serosa	☐	☐	☐	☐	☐	☐	☐	☐	☐	☐	☐	☐
Other location #1, left	☐	☐	☐	☐	☐	☐	☐	☐	☐	☐	☐	☐
Other location #2, left	☐	☐	☐	☐	☐	☐	☐	☐	☐	☐	☐	☐

Size of vascular lesion on left pelvic sidewall? ◯ < 1cm ◯ 1-3 cm ◯ > 3 cm

Size of clear lesion on left pelvic sidewall? ◯ < 1cm ◯ 1-3 cm ◯ > 3 cm

Size of yellow lesion on left pelvic sidewall? ◯ < 1cm ◯ 1-3 cm ◯ > 3 cm

Size of red lesion on left pelvic sidewall? ◯ < 1cm ◯ 1-3 cm ◯ > 3 cm

Size of white lesion on left pelvic sidewall? ◯ < 1cm ◯ 1-3 cm ◯ > 3 cm

Size of blue/black lesion on left pelvic sidewall?	○ < 1cm ○ 1-3 cm ○ > 3 cm
Size of brown lesion on left pelvic sidewall?	○ < 1cm ○ 1-3 cm ○ > 3 cm
Size of vascular lesion on left utero-sacral ligament?	○ < 1cm ○ 1-3 cm ○ > 3 cm
Size of clear lesion on left utero-sacral ligament?	○ < 1cm ○ 1-3 cm ○ > 3 cm
Size of yellow lesion on left utero-sacral ligament?	○ < 1cm ○ 1-3 cm ○ > 3 cm
Size of red lesion on left utero-sacral ligament?	○ < 1cm ○ 1-3 cm ○ > 3 cm
Size of white lesion on left utero-sacral ligament?	○ < 1cm ○ 1-3 cm ○ > 3 cm
Size of blue/black lesion on left utero-sacral ligament?	○ < 1cm ○ 1-3 cm ○ > 3 cm
Size of brown lesion on left utero-sacral ligament?	○ < 1cm ○ 1-3 cm ○ > 3 cm
Size of vascular lesion on left ovarian serosa?	○ < 1cm ○ 1-3 cm ○ > 3 cm
Size of clear lesion on left ovarian serosa?	○ < 1cm ○ 1-3 cm ○ > 3 cm
Size of yellow lesion on left ovarian serosa?	○ < 1cm ○ 1-3 cm ○ > 3 cm
Size of red lesion on left ovarian serosa?	○ < 1cm ○ 1-3 cm ○ > 3 cm
Size of white lesion on left ovarian serosa?	○ < 1cm ○ 1-3 cm ○ > 3 cm
Size of blue/black lesion on left ovarian serosa?	○ < 1cm ○ 1-3 cm ○ > 3 cm
Size of brown lesion on left ovarian serosa?	○ < 1cm ○ 1-3 cm ○ > 3 cm
Size of vascular lesion on left tubal serosa?	○ < 1cm ○ 1-3 cm ○ > 3 cm
Size of clear lesion on left tubal serosa?	○ < 1cm ○ 1-3 cm ○ > 3 cm
Size of yellow lesion on left tubal serosa?	○ < 1cm ○ 1-3 cm ○ > 3 cm
Size of red lesion on left tubal serosa?	○ < 1cm ○ 1-3 cm ○ > 3 cm
Size of white lesion on left tubal serosa?	○ < 1cm ○ 1-3 cm ○ > 3 cm
Size of blue/black lesion on left tubal serosa?	○ < 1cm ○ 1-3 cm ○ > 3 cm
Size of brown lesion on left tubal serosa?	○ < 1cm ○ 1-3 cm ○ > 3 cm
Other location, #1, left:	__________________________

Size of vascular lesion on left "other location #1":	◯ < 1cm ◯ 1-3 cm ◯ > 3 cm
Size of clear lesion on left "other location #1":	◯ < 1cm ◯ 1-3 cm ◯ > 3 cm
Size of yellow lesion on left "other location #1":	◯ < 1cm ◯ 1-3 cm ◯ > 3 cm
Size of red lesion on left "other location #1":	◯ < 1cm ◯ 1-3 cm ◯ > 3 cm
Size of white lesion on left "other location #1":	◯ < 1cm ◯ 1-3 cm ◯ > 3 cm
Size of blue/black lesion on left "other location #1":	◯ < 1cm ◯ 1-3 cm ◯ > 3 cm
Size of brown lesion on left "other location #1":	◯ < 1cm ◯ 1-3 cm ◯ > 3 cm
Other location, #2, left:	__________________________
Size of vascular lesion on left "other location #2":	◯ < 1cm ◯ 1-3 cm ◯ > 3 cm
Size of clear lesion on left "other location #2":	◯ < 1cm ◯ 1-3 cm ◯ > 3 cm
Size of yellow lesion on left "other location #2":	◯ < 1cm ◯ 1-3 cm ◯ > 3 cm
Size of red lesion on left "other location #2":	◯ < 1cm ◯ 1-3 cm ◯ > 3 cm
Size of white lesion on left "other location #2":	◯ < 1cm ◯ 1-3 cm ◯ > 3 cm
Size of blue/black lesion on left "other location #2":	◯ < 1cm ◯ 1-3 cm ◯ > 3 cm
Size of brown lesion on left "other location #2":	◯ < 1cm ◯ 1-3 cm ◯ > 3 cm

Location of endometriosis RIGHT

	Vascular	Clear	Yellow	Red	White	Blue/ Black	Brown	Filmy adhesions	Web adhesions	Thin adhesions	Dense adhesions	Sac like adhesions
Right pelvic sidewall	☐	☐	☐	☐	☐	☐	☐	☐	☐	☐	☐	☐
Right uterosacral ligament	☐	☐	☐	☐	☐	☐	☐	☐	☐	☐	☐	☐
Right ovary - serosa	☐	☐	☐	☐	☐	☐	☐	☐	☐	☐	☐	☐
Right tube - serosa	☐	☐	☐	☐	☐	☐	☐	☐	☐	☐	☐	☐
Other location #1, right	☐	☐	☐	☐	☐	☐	☐	☐	☐	☐	☐	☐
Other location #2, right	☐	☐	☐	☐	☐	☐	☐	☐	☐	☐	☐	☐

Size of vascular lesion on right pelvic sidewall?	◯ < 1cm ◯ 1-3 cm ◯ > 3 cm
Size of clear lesion on right pelvic sidewall?	◯ < 1cm ◯ 1-3 cm ◯ > 3 cm
Size of yellow lesion on right pelvic sidewall?	◯ < 1cm ◯ 1-3 cm ◯ > 3 cm

Size of red lesion on right pelvic sidewall?	◯ < 1cm	◯ 1-3 cm	◯ > 3 cm
Size of white lesion on right pelvic sidewall?	◯ < 1cm	◯ 1-3 cm	◯ > 3 cm
Size of blue/black lesion on right pelvic sidewall?	◯ < 1cm	◯ 1-3 cm	◯ > 3 cm
Size of brown lesion on right pelvic sidewall?	◯ < 1cm	◯ 1-3 cm	◯ > 3 cm
Size of vascular lesion on right utero-sacral ligament?	◯ < 1cm	◯ 1-3 cm	◯ > 3 cm
Size of clear lesion on right utero-sacral ligament?	◯ < 1cm	◯ 1-3 cm	◯ > 3 cm
Size of yellow lesion on right utero-sacral ligament?	◯ < 1cm	◯ 1-3 cm	◯ > 3 cm
Size of red lesion on right utero-sacral ligament?	◯ < 1cm	◯ 1-3 cm	◯ > 3 cm
Size of white lesion on right utero-sacral ligament?	◯ < 1cm	◯ 1-3 cm	◯ > 3 cm
Size of blue/black lesion on right utero-sacral ligament?	◯ < 1cm	◯ 1-3 cm	◯ > 3 cm
Size of brown lesion on right utero-sacral ligament?	◯ < 1cm	◯ 1-3 cm	◯ > 3 cm
Size of vascular lesion on right ovarian serosa?	◯ < 1cm	◯ 1-3 cm	◯ > 3 cm
Size of clear lesion on right ovarian serosa?	◯ < 1cm	◯ 1-3 cm	◯ > 3 cm
Size of yellow lesion on right ovarian serosa?	◯ < 1cm	◯ 1-3 cm	◯ > 3 cm
Size of red lesion on right ovarian serosa?	◯ < 1cm	◯ 1-3 cm	◯ > 3 cm
Size of white lesion on right ovarian serosa?	◯ < 1cm	◯ 1-3 cm	◯ > 3 cm
Size of blue/black lesion on right ovarian serosa?	◯ < 1cm	◯ 1-3 cm	◯ > 3 cm
Size of brown lesion on right ovarian serosa?	◯ < 1cm	◯ 1-3 cm	◯ > 3 cm
Size of vascular lesion on right tubal serosa?	◯ < 1cm	◯ 1-3 cm	◯ > 3 cm
Size of clear lesion on right tubal serosa?	◯ < 1cm	◯ 1-3 cm	◯ > 3 cm
Size of yellow lesion on right tubal serosa?	◯ < 1cm	◯ 1-3 cm	◯ > 3 cm
Size of red lesion on right tubal serosa?	◯ < 1cm	◯ 1-3 cm	◯ > 3 cm
Size of white lesion on right tubal serosa?	◯ < 1cm	◯ 1-3 cm	◯ > 3 cm
Size of blue/black lesion on right tubal serosa?	◯ < 1cm	◯ 1-3 cm	◯ > 3 cm

Size of brown lesion on right tubal serosa?	◯ < 1cm ◯ 1-3 cm ◯ > 3 cm
Other location #1 right, please specify:	__________________________
Size of vascular lesion on right "other location #1"?	◯ < 1cm ◯ 1-3 cm ◯ > 3 cm
Size of clear lesion on right "other location #1"?	◯ < 1cm ◯ 1-3 cm ◯ > 3 cm
Size of yellow lesion on right "other location #1"?	◯ < 1cm ◯ 1-3 cm ◯ > 3 cm
Size of red lesion on right "other location #1"?	◯ < 1cm ◯ 1-3 cm ◯ > 3 cm
Size of white lesion on right "other location #1"?	◯ < 1cm ◯ 1-3 cm ◯ > 3 cm
Size of blue/black lesion on right "other location #1"?	◯ < 1cm ◯ 1-3 cm ◯ > 3 cm
Size of brown lesion on right "other location #1"?	◯ < 1cm ◯ 1-3 cm ◯ > 3 cm
Other location #2 right, please specify:	__________________________
Size of vascular lesion on right "other location #2"?	◯ < 1cm ◯ 1-3 cm ◯ > 3 cm
Size of clear lesion on right "other location #2"?	◯ < 1cm ◯ 1-3 cm ◯ > 3 cm
Size of yellow lesion on right "other location #2"?	◯ < 1cm ◯ 1-3 cm ◯ > 3 cm
Size of red lesion on right "other location #2"?	◯ < 1cm ◯ 1-3 cm ◯ > 3 cm
Size of white lesion on right "other location #2"?	◯ < 1cm ◯ 1-3 cm ◯ > 3 cm
Size of blue/black lesion on right "other location #2"?	◯ < 1cm ◯ 1-3 cm ◯ > 3 cm
Size of brown lesion on right "other location #2"?	◯ < 1cm ◯ 1-3 cm ◯ > 3 cm

Location of endometriosis CENTRAL

	Vascular	Clear	Yellow	Red	White	Blue/ Black	Brown	Filmy adhesions	Web adhesions	Thin adhesions	Dense adhesions	Sac like adhesions
Uterovesical pouch/anterior cul-de-sac	☐	☐	☐	☐	☐	☐	☐	☐	☐	☐	☐	☐
Pouch of Douglas/posterior cul-de-sac	☐	☐	☐	☐	☐	☐	☐	☐	☐	☐	☐	☐

Uterus - serosa	☐	☐	☐	☐	☐	☐	☐	☐	☐	☐	☐	☐
Bladder - deep inflitrating	☐	☐	☐	☐	☐	☐	☐	☐	☐	☐	☐	☐
Bladder - serosa	☐	☐	☐	☐	☐	☐	☐	☐	☐	☐	☐	☐
Colon - deep infiltrating	☐	☐	☐	☐	☐	☐	☐	☐	☐	☐	☐	☐
Colon - serosa	☐	☐	☐	☐	☐	☐	☐	☐	☐	☐	☐	☐
Vagina	☐	☐	☐	☐	☐	☐	☐	☐	☐	☐	☐	☐
Other, location #1	☐	☐	☐	☐	☐	☐	☐	☐	☐	☐	☐	☐
Other, location #2	☐	☐	☐	☐	☐	☐	☐	☐	☐	☐	☐	☐

Size of vascular lesion on uterovesical pouch/anterior cul-de-sac? ◯ < 1cm ◯ 1-3 cm ◯ > 3 cm

---

Size of clear lesion on uterovesical pouch/anterior cul-de-sac? ◯ < 1cm ◯ 1-3 cm ◯ > 3 cm

---

Size of yellow lesion on uterovesical pouch/anterior cul-de-sac? ◯ < 1cm ◯ 1-3 cm ◯ > 3 cm

---

Size of red lesion on uterovesical pouch/anterior cul-de-sac? ◯ < 1cm ◯ 1-3 cm ◯ > 3 cm

---

Size of white lesion on uterovesical pouch/anterior cul-de-sac? ◯ < 1cm ◯ 1-3 cm ◯ > 3 cm

---

Size of blue/black lesion on uterovesical pouch/anterior cul-de-sac? ◯ < 1cm ◯ 1-3 cm ◯ > 3 cm

---

Size of brown lesion on uterovesical pouch/anterior cul-de-sac? ◯ < 1cm ◯ 1-3 cm ◯ > 3 cm

---

Size of vascular lesion Pouch of Douglas/posterior cul-de-sac? ◯ < 1cm ◯ 1-3 cm ◯ > 3 cm

---

Size of clear lesion Pouch of Douglas/posterior cul-de-sac? ◯ < 1cm ◯ 1-3 cm ◯ > 3 cm

---

Size of yellow lesion Pouch of Douglas/posterior cul-de-sac? ◯ < 1cm ◯ 1-3 cm ◯ > 3 cm

---

Size of red lesion Pouch of Douglas/posterior cul-de-sac? ◯ < 1cm ◯ 1-3 cm ◯ > 3 cm

---

Size of white lesion Pouch of Douglas/posterior cul-de-sac? ◯ < 1cm ◯ 1-3 cm ◯ > 3 cm

---

Size of blue/black lesion Pouch of Douglas/posterior cul-de-sac? ◯ < 1cm ◯ 1-3 cm ◯ > 3 cm

---

Size of brown lesion Pouch of Douglas/posterior cul-de-sac? ◯ < 1cm ◯ 1-3 cm ◯ > 3 cm

Size of vascular lesion on uterine serosa?	◯ < 1cm ◯ 1-3 cm ◯ > 3 cm
Size of clear lesion on uterine serosa?	◯ < 1cm ◯ 1-3 cm ◯ > 3 cm
Size of yellow lesion on uterine serosa?	◯ < 1cm ◯ 1-3 cm ◯ > 3 cm
Size of red lesion on uterine serosa?	◯ < 1cm ◯ 1-3 cm ◯ > 3 cm
Size of white lesion on uterine serosa?	◯ < 1cm ◯ 1-3 cm ◯ > 3 cm
Size of blue/black lesion on uterine serosa?	◯ < 1cm ◯ 1-3 cm ◯ > 3 cm
Size of brown lesion on uterine serosa?	◯ < 1cm ◯ 1-3 cm ◯ > 3 cm
Size of deep infiltrating vascular lesion on bladder?	◯ < 1cm ◯ 1-3 cm ◯ > 3 cm
Size of deep infiltrating clear lesion on bladder?	◯ < 1cm ◯ 1-3 cm ◯ > 3 cm
Size of deep infiltrating yellow lesion on bladder?	◯ < 1cm ◯ 1-3 cm ◯ > 3 cm
Size of deep infiltrating red lesion on bladder?	◯ < 1cm ◯ 1-3 cm ◯ > 3 cm
Size of deep infiltrating white lesion on bladder?	◯ < 1cm ◯ 1-3 cm ◯ > 3 cm
Size of deep infiltrating blue/black lesion on bladder?	◯ < 1cm ◯ 1-3 cm ◯ > 3 cm
Size of deep infiltrating brown lesion on bladder?	◯ < 1cm ◯ 1-3 cm ◯ > 3 cm
Size of vascular lesion on bladder serosa?	◯ < 1cm ◯ 1-3 cm ◯ > 3 cm
Size of clear lesion on bladder serosa?	◯ < 1cm ◯ 1-3 cm ◯ > 3 cm
Size of yellow lesion on bladder serosa?	◯ < 1cm ◯ 1-3 cm ◯ > 3 cm
Size of red lesion on bladder serosa?	◯ < 1cm ◯ 1-3 cm ◯ > 3 cm
Size of white lesion on bladder serosa?	◯ < 1cm ◯ 1-3 cm ◯ > 3 cm
Size of blue/black lesion on bladder serosa?	◯ < 1cm ◯ 1-3 cm ◯ > 3 cm
Size of brown lesion on bladder serosa?	◯ < 1cm ◯ 1-3 cm ◯ > 3 cm
Size of deep infiltrating vascular lesion on colon?	◯ < 1cm ◯ 1-3 cm ◯ > 3 cm
Size of deep infiltrating clear lesion on colon?	◯ < 1cm ◯ 1-3 cm ◯ > 3 cm
Size of deep infiltrating yellow lesion on colon?	◯ < 1cm ◯ 1-3 cm ◯ > 3 cm
Size of deep infiltrating red lesion on colon?	◯ < 1cm ◯ 1-3 cm ◯ > 3 cm

Size of deep infiltrating white lesion on colon?	◯ < 1cm ◯ 1-3 cm ◯ > 3 cm
Size of deep infiltrating blue/black lesion on colon?	◯ < 1cm ◯ 1-3 cm ◯ > 3 cm
Size of deep infiltrating brown lesion on colon?	◯ < 1cm ◯ 1-3 cm ◯ > 3 cm
Size of vascular lesion on colonic serosa?	◯ < 1cm ◯ 1-3 cm ◯ > 3 cm
Size of clear lesion on colonic serosa?	◯ < 1cm ◯ 1-3 cm ◯ > 3 cm
Size of yellow lesion on colonic serosa?	◯ < 1cm ◯ 1-3 cm ◯ > 3 cm
Size of red lesion on colonic serosa?	◯ < 1cm ◯ 1-3 cm ◯ > 3 cm
Size of white lesion on colonic serosa?	◯ < 1cm ◯ 1-3 cm ◯ > 3 cm
Size of blue/black lesion on colonic serosa?	◯ < 1cm ◯ 1-3 cm ◯ > 3 cm
Size of brown lesion on colonic serosa?	◯ < 1cm ◯ 1-3 cm ◯ > 3 cm
Size of vascular lesion on vagina?	◯ < 1cm ◯ 1-3 cm ◯ > 3 cm
Size of clear lesion on vagina?	◯ < 1cm ◯ 1-3 cm ◯ > 3 cm
Size of yellow lesion on vagina?	◯ < 1cm ◯ 1-3 cm ◯ > 3 cm
Size of red lesion on vagina?	◯ < 1cm ◯ 1-3 cm ◯ > 3 cm
Size of white lesion on vagina?	◯ < 1cm ◯ 1-3 cm ◯ > 3 cm
Size of blue/black lesion on vagina?	◯ < 1cm ◯ 1-3 cm ◯ > 3 cm
Size of brown lesion on vagina?	◯ < 1cm ◯ 1-3 cm ◯ > 3 cm
Other location #1, please specify:	______________________________
Size of vascular lesion on central "other location #1"?	◯ < 1cm ◯ 1-3 cm ◯ > 3 cm
Size of clear lesion on central "other location #1"?	◯ < 1cm ◯ 1-3 cm ◯ > 3 cm
Size of yellow lesion on central "other location #1"?	◯ < 1cm ◯ 1-3 cm ◯ > 3 cm
Size of red lesion on central "other location #1"?	◯ < 1cm ◯ 1-3 cm ◯ > 3 cm
Size of white lesion on central "other location #1"?	◯ < 1cm ◯ 1-3 cm ◯ > 3 cm
Size of blue/black lesion on central "other location #1"?	◯ < 1cm ◯ 1-3 cm ◯ > 3 cm

Size of brown lesion on central "other location #1"?	◯ < 1cm ◯ 1-3 cm ◯ > 3 cm
Other location #2, please specify:	______________________________
Size of vascular lesion on central "other location #2"?	◯ < 1cm ◯ 1-3 cm ◯ > 3 cm
Size of clear lesion on central "other location #2"?	◯ < 1cm ◯ 1-3 cm ◯ > 3 cm
Size of yellow lesion on central "other location #2"?	◯ < 1cm ◯ 1-3 cm ◯ > 3 cm
Size of red lesion on central "other location #2"?	◯ < 1cm ◯ 1-3 cm ◯ > 3 cm
Size of white lesion on central "other location #2"?	◯ < 1cm ◯ 1-3 cm ◯ > 3 cm
Size of blue/black lesion on central "other location #2"?	◯ < 1cm ◯ 1-3 cm ◯ > 3 cm
Size of brown lesion on central "other location #2"?	◯ < 1cm ◯ 1-3 cm ◯ > 3 cm
Peritoneal pouches/pockets?	◯ Yes ◯ No
How many?	______________________________
Location pouch #1:	______________________________
Depth pouch #1:	______________________________ (cm)
Diameter pouch #1:	______________________________
Location pouch #2:	______________________________
Depth pouch #2:	______________________________ (cm)
Diameter pouch #2:	______________________________
Location pouch #3:	______________________________
Depth pouch #3:	______________________________ (cm)
Diameter pouch #3:	______________________________

Diaphragmatic endometriosis?	◯ Yes ◯ No
If yes,	☐ Left ☐ Right
Left, describe:	__________________
Right, describe:	__________________
Biopsy taken?	◯ Yes ◯ No
How many?	__________________
Location #1, please specify:	__________________
Location #2, please specify:	__________________
Location #3, please specify:	__________________
Location #4, please specify:	__________________
Location #5, please specify:	__________________
Location #6, please specify:	__________________
Control biopsy taken?	◯ Yes ◯ No
How many?	__________________
Location #1, please specify:	__________________
Location #2, please specify:	__________________
Location #3, please specify:	__________________

X. Endometrioma

Endometrioma? ◯ Yes ◯ No

Endometrioma(s) location: ☐ Left ☐ Right

Left endometrioma - how many? ________________ (Max 3)

Left endometrioma 1 - size: ________________ (cm)

Left endometrioma 2 - size: ________________ (cm)

Left endometrioma 3 - size: ________________ (cm)

Right endometrioma - how many? ________________ (Max 3)

Right endometrioma 1 - size: ________________ (cm)

Right endometrioma 2 - size: ________________ (cm)

Right endometrioma 3 - size: ________________ (cm)

Sent to histology? ◯ Yes ◯ No

Sample collected for research: ☐ Left ☐ Right ☐ None

XI. Endometriotic nodule

Endometriotic nodule:
☐ Pouch of Douglas
☐ Vagina
☐ Bladder
☐ Appendix
☐ Small bowel
☐ Sigmoid colon
☐ Rectum
☐ None

Pouch of Douglas - size in 3 dimensions: __ x __ x __ cm ________________ (cm)

Pouch of Douglas nodule, full thickness?	◯ Yes ◯ No
Vagina - size in 3 dimensions: __ x __ x __ cm	______________ (cm)
Vaginal nodule, full thickness?	◯ Yes ◯ No
Bladder - size in 3 dimensions: __ x __ x __ cm	______________ (cm)
Bladder nodule, full thickness?	◯ Yes ◯ No
Appendix - size in 3 dimensions: __ x __ x __ cm	______________ (cm)
Appendix nodule, full thickness?	◯ Yes ◯ No
Small bowel - size in 3 dimensions: __ x __ x __ cm	______________ (cm)
Small bowel nodule, full thickness?	◯ Yes ◯ No
Sigmoid colon - size in 3 dimensions: __ x __ x __ cm	______________ (cm)
Sigmoid colon - distance from anus	______________ (cm)
Sigmoid colon, full thickness?	◯ Yes ◯ No
Rectum - size in 3 dimensions: __ x __ x __ cm	______________ (cm)
Rectum - distance from anus	______________ (cm)
Rectum, full thickness?	◯ Yes ◯ No
Second endometriotic nodule?	◯ Yes ◯ No
Second endometriotic nodule:	☐ Pouch of Douglas<br>☐ Vagina<br>☐ Bladder<br>☐ Appendix<br>☐ Small bowel<br>☐ Sigmoid colon<br>☐ Rectum

Pouch of Douglas - size in 3 dimensions: __ x __ x ___ cm	______________ (cm)
Pouch of Douglas nodule, full thickness?	◯ Yes ◯ No
Vagina - size in 3 dimensions: __ x __ x __ cm	______________ (cm)
Vaginal nodule, full thickness?	◯ Yes ◯ No
Bladder - size in 3 dimensions: __ x __ x __ cm	______________ (cm)
Bladder nodule, full thickness?	◯ Yes ◯ No
Appendix - size in 3 dimensions: __ x __ x __ cm	______________ (cm)
Appendix nodule, full thickness?	◯ Yes ◯ No
Small bowel - size in 3 dimensions: __ x __ x __ cm	______________ (cm)
Small bowel nodule, full thickness?	◯ Yes ◯ No
Sigmoid colon - size in 3 dimensions: __ x __ x __ cm	______________ (cm)
Sigmoid colon, full thickness?	◯ Yes ◯ No
Sigmoid colon - distance from anus	______________ (cm)
Rectum - size in 3 dimensions: __ x __ x __ cm	______________ (cm)
Rectum - distance from anus	______________ (cm)
Rectum, full thickness?	◯ Yes ◯ No

XII. Additional Findings

Fibroids (Myoma)	◯ Yes ◯ No
Fibroids (Myoma):	☐ Submucous<br>☐ Intramural<br>☐ Subserous
Fibroids (Myoma) - Submucous - number:	__________________________________
Fibroid - Submucous 1- size:	__________________________________<br>(cm)
Fibroid - Submucous 2 - size:	__________________________________<br>(cm)
Fibroids (Myoma) - Intramural - number:	__________________________________<br>(max. 3)
Fibroid - Intramural 1- size:	__________________________________<br>(cm)
Fibroid - Intramural 2 - size:	__________________________________<br>(cm)
Fibroids (Myoma) - Subserous - number:	__________________________________<br>(max. 3)
Fibroid - Subserous 1- size:	__________________________________<br>(cm)
Fibroid - Subserous 2 - size:	__________________________________<br>(cm)
Adhesions (without evidence of endometriosis)	◯ Yes ◯ No
Type:	☐ Filmy<br>☐ Dense<br>☐ Co-apted<br>☐ Obstructed
Filmy adhesions - location(s):	__________________________________
Dense adhesions - location(s):	__________________________________

Co-apted adhesions - location(s): __________________________________

Obstructed adhesions - location(s): __________________________________

Congenital anomaly ◯ Yes ◯ No

Congenital anomaly - types: ____

Non-endometriotic ovarian cyst ◯ Yes ◯ No

Non-endometriotic ovarian cyst - side: ☐ Left ☐ Right

Left non-endometriotic ovarian cyst, suspected type: __________________________________

Right non-endometriotic ovarian cyst, suspected type: __________________________________

Any other findings:

Procedure was:
◯ More complex/difficult than expected
◯ As complex/difficult as expected
◯ Less complicated/difficult than expected

XIII. Endometriosis Fertility Index (EFI)

ENDOMETRIOSIS FERTILITY INDEX (EFI)
SURGERY FORM

LEAST FUNCTION (LF) SCORE AT CONCLUSION OF SURGERY

Score		Description
4	=	Normal
3	=	Mild Dysfunction
2	=	Moderate Dysfunction
1	=	Severe Dysfunction
0	=	Absent or Nonfunctional

	Left	Right
Fallopian Tube	☐	☐
Fimbria	☐	☐
Ovary	☐	☐

To calculate the LF score, add together the lowest score for the left side and the lowest score for the right side. If an ovary is absent on one side, the LF score is obtained by doubling the lowest score on the side with the ovary.

Lowest Score ☐ + ☐ = ☐
Left Right LF Score

background-color:#EBFFCC"">Least Function (LF) Score at Conclusion of Surgery" __________________________________

Endometriosis Fertility Index (EFI)

Historical Factors	
Age	◯ Age =< 35 years<br>◯ Age 36 to 39<br>◯ Age >= 40 years
Years infertile	◯ Years infertile =< 3<br>◯ Years infertile >3
Prior Pregnancy:	◯ History of prior pregnancy<br>◯ No history of prior pregnancy
Total historical factors	__________________
Surgical Factors	
LF score: [s_efi_lfscore]	◯ If LF Score = 7 to 8 (high score)<br>◯ If LF Score = 4 to 6 (moderate score)<br>◯ If LF Score = 1 to 3 (low score)
AFS endometriosis score: [s_afs_endoscore]	◯ If AFS Endometriosis Lesion Score is < 16<br>◯ If AFS Endometriosis Lesion Score is >=16
AFS Total Score: [s_afs_totalscore]	◯ If AFS total score is < 71<br>◯ If AFS total score is >= 71
Total Surgcial Factors	__________________
EFI Score (Historical + Surgical)	__________________
Data entered retrospectively?	◯ Yes
If surgical stage was noted in the OR report. Please enter here:	◯ Stage 1<br>◯ Stage 2<br>◯ Stage 3<br>◯ Stage 4<br>◯ No stage mentioned/unclear<br>(only for retrospective entry when AFS score is missing)

Suppression hormonale post-opératoire

Suppression hormonale en post-opératoire?	◯ Oui ◯ Non
Si oui, qu'avez-vous prescrit ?	☐ Inhibiteur de l'aromatase<br>☐ COC<br>☐ Progestatif<br>☐ Stérilet de levonorgestrel<br>☐ Danazol<br>☐ Agoniste de la GnRH<br>☐ Antagoniste de la GnRH<br>☐ SPRM

Inhibiteur de l'aromatase :	☐ Letrozole ☐ Autre
Si autre inhibiteur de l'aromatase, veuillez spécifier :	__________________________
Spécifier type COC :	◯ Continu<br>◯ Cyclique
COC :	◯ Alesse ◯ Yasmin ◯ Yaz<br>◯ Marvelon ◯ Brevicon<br>◯ Cyclen ◯ MinOvral<br>◯ Seasonale/ Seasonique<br>◯ Evra patch ◯ NuvaRing<br>◯ LoLo ◯ Other
Spécifier progestatif :	☐ Provera ☐ Visanne<br>☐ Norlutate ☐ Micronor
Provera - spécifier la dose :	__________________________<br>(mg)
Visanne - spécifier la dose :	__________________________<br>(mg)
Norlutate - spécifier la dose :	__________________________<br>(mg)
Micronor - spécifier la dose :	__________________________<br>(mg)
IUD	☐ Mirena ☐ Kyleena<br>☐ Jaydess
Danazol :	☐ Oral ☐ Vaginal
Agoniste de la GnRH :	☐ Lupron 11.25 mg IM q 3 mois<br>☐ Lupron 3.75 mg IM q 1 mois<br>☐ Synarel<br>☐ Autre
Si autre agoniste de la GnRH, veuillez spécifier :	__________________________
Antagoniste de la GnRH :	◯ Elagolix (orlissa) 150 mg PO die<br>◯ Elagolix (orlissa) 200 mg PO BID
SPRM :	☐ Fibristal ☐ Autre
Si autre SPRM, veuillez spécifier :	__________________________

Suppression hormonale : combien de temps (en mois)?

____
(mois)

Comments

Pathology

Identifiant : ______________________________

Enter the overall endometriosis pathology result for the PERSON

Date of surgery [visite_intiale_cli_arm_2][s_date]

Any histologically confirmed endometriosis ◯ Yes ◯ No

Type of Endometriosis
- ☐ Superficial Endometriosis
- ☐ Deep Infiltrating Endometriosis
- ☐ Endometrioma
- ☐ Unclear
- ☐ Other

(you may choose more than one)

Other : ______________________________

No Pathology Report? ◯ Surgery completed but no pathology
