# Supplementary material for: Quality of life and symptoms of pain in patients with endometriomas compared to those with other endometriosis lesions: a cross-sectional study
Source: BMC Womens Health. 2024 Jan 27;24:72. doi: 10.1186/s12905-024-02919-1 (PMC10821264; doi:10.1186/s12905-024-02919-1)
Supplement: Supplementary file 2 — Additional file 2: Table S1. Frequency and percentage of endometriosis subtypes in the study sample. Table S2. Number and size of endometriomas in participants with surgical diagnosis. Figure S1. Subgroup analyses of mean difference in superficial dyspareunia intensity of patients with endometrioma compared to those with other types of endometriosis regarding potential modification factors. Figure S2. Subgroup analyses of mean difference in chronic pelvic pain intensity of patients with endometrioma compared to those with other types of endometriosis regarding potential modification factors. Figure S3. Subgroup analyses of mean difference in deep dyspareunia intensity of patients with endometrioma compared to those with other types of endometriosis regarding potential modification factors. Figure S4. Subgroup analyses of mean difference in lower back pain intensity of patients with endometrioma compared to those with other types of endometriosis regarding potential modification factors. Figure S5. Subgroup analyses of mean difference in dysmenorrhea intensity of patients with endometrioma compared to those with other types of endometriosis regarding potential modification factors. Figure S6. Subgroup analyses of mean difference in dyschezia intensity of patients with endometrioma compared to those with other types of endometriosis regarding potential modification factors. Figure S7. Impaired quality of life and severe pain symptoms of patients with endometriomas compared to those with other lesions of endometriosis. [file 12905_2024_2919_MOESM2_ESM.pdf]

Quality of life and symptoms of pain in patients with endometriomas compared to those with other endometriosis lesions: a cross-sectional study

Authors and Affiliations

Fleur Serge Kanti[1*]

Valérie Allard[1]

Sarah Maheux-Lacroix[1]

1. Centre hospitalier universitaire de Québec - Université Laval, Quebec City, Quebec, Canada.

*** Corresponding Author**

Fleur Serge Kanti

Centre hospitalier universitaire de Québec - Université Laval

2705, boulevard Laurier

T0-64

Québec, Québec

Canada, G1V 4G2

Phone: +1 418 525 4444, # 46185

E-mail: serge.kanti@crchudequebec.ulaval.ca

SUPPLEMENTARY FILE 2

Table S1. Frequency and percentage of endometriosis subtypes in the study sample

Endometriosis [a]	Total (N = 248)
Superficial	51 (20.6%)
Deep	180 (72.6%)
Ovarian	81 (32.7%)

Abbreviations: N, sample size.

Notes:

[a] Patients may have at least one endometriosis lesion. Endometriosis subtypes have been described separately. Values are given in frequency (percentage).

Table S2. Number and size of endometriomas in participants with surgical diagnosis

**Variables** [a]	**N = 36**
Number of endometriomas lesions	
Mean ± SD	1.5 ± 1.3
Minimum-maximum	[1 to 7]
Maximum size of all endometriomas lesions (10^{-2} m)	
Mean ± SD	5 ± 3.0
Minimum-maximum	[1 to 11.0]
Median (IQR)	5 (2.8 - 7)
Sum of the size of all endometriomas lesions (10^{-2} m)	
Mean ± SD	6 ± 4.5
Minimum-maximum	[1 to 16.0]
Median (IQR)	6 (2.8 - 9)
Mean of the size of all endometriomas lesions (10^{-2} m)	
Mean ± SD	5 ± 2.8
Minimum-maximum	[1 to 10.0]
Median (IQR)	5 (2.2 - 6)

Abbreviations: N, sample size; SD, standard deviation; IQR, interquartile range.

Notes:

[a] Variables are indicated at participant level.

Figure S1-S6 legends. Subgroup analyses of the mean difference in pain intensity of patients with endometrioma compared to those with other types of endometriosis regarding potential modification factors.

Abbreviations: CI, Confidence interval.

Notes:

β denotes the linear regression coefficient (mean difference in pain symptom score between individuals with endometriomas and those without endometriomas).

The modification factors are the diagnosis method (imaging modalities/histology), concurrent pain symptoms (1-4/5-6), comorbid infertility (yes/no), presence of adenomyosis (yes/no), presence of fibroma (yes/no), moderate depression symptoms (yes/no), moderate anxiety symptoms (yes/no), pain catastrophizing (yes/no) and central component of pain (yes/no).

The P value for each factor was used for determining the association between the pain intensity and the presence of endometrioma (modification factor if $P < 0.05$; not otherwise), allowing to compare the mean difference between modification factor levels. For each level of each factor, the mean difference in the pain intensity between patients with endometriomas and those with other types of endometriosis is indicated by the blue square (point estimate value), with the 95% confidence interval delimited by the black horizontal solid line.

The vertical black dashed line represents the null value (zero) of the mean difference, indicating that the mean difference is significantly different from zero when the confidence interval does not include zero (equivalent to $p < 0.05$; not shown here).

The models were adjusted for age, body mass index (kg/m^2), ethnicity, age of menarche, parity, education level, employment status, marital status, annual income, hormone use in the last three months, and each modification factor using an interaction term with the endometriosis type variable.

Stars highlight p-values less than 0.05.

Superficial dyspareunia

Modification factor	β [95%CI]	P-value
Diagnosis method		0.618
Imaging modalities	0.708 [-0.94 to 2.36]	
Surgery/Histology	1.317 [-0.46 to 3.09]	
Concurrent pain symptoms		0.313
1-4	2.944 [0.02 to 5.87]	
5-6	1.261 [-0.12 to 2.64]	
Infertility		0.049*
Yes	4.639 [1.15 to 8.12]	
No	0.928 [-0.34 to 2.19]	
Adenomyosis		0.738
Yes	2.088 [-2.97 to 7.15]	
No	1.163 [-1.12 to 3.44]	
Fibroma		0.494
Yes	2.598 [-0.99 to 6.19]	
No	0.563 [-4.24 to 5.37]	
Moderate depression		0.952
Yes	2.274 [-1.55 to 6.09]	
No	2.429 [-0.97 to 5.82]	
Moderate anxiety		0.411
Yes	4.304 [0.39 to 8.22]	
No	2.41 [-0.35 to 5.17]	
Central component of pain		0.953
Yes	1.724 [-2.09 to 5.54]	
No	1.588 [-0.53 to 3.7]	
Pain catastrophizing		0.201
Yes	2.599 [0.31 to 4.88]	
No	0.885 [-0.5 to 2.27]	

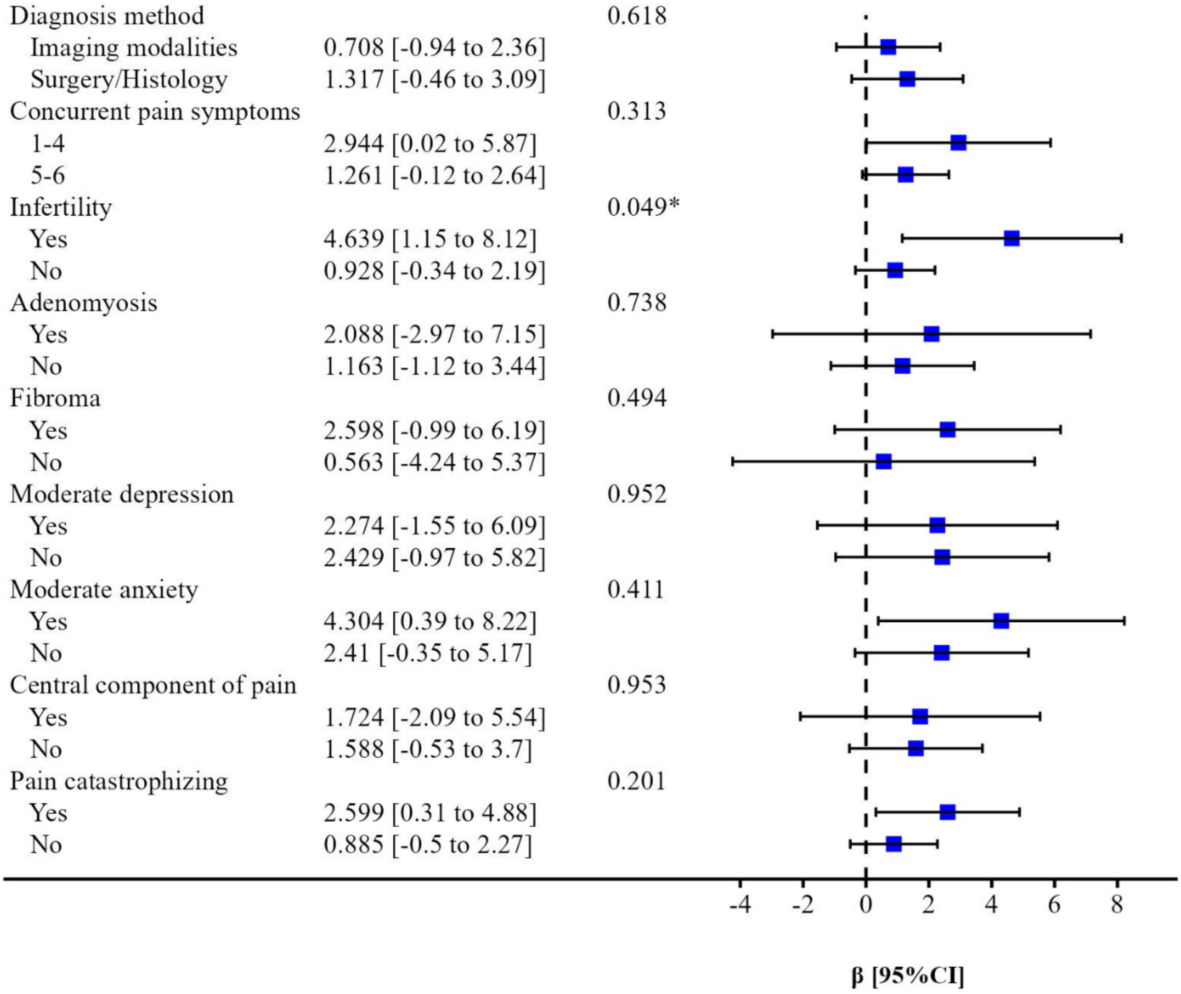

Figure S1. Subgroup analyses of mean difference in superficial dyspareunia intensity of patients with endometrioma compared to those with other types of endometriosis regarding potential modification factors.

Chronic pelvic pain

Modification factor	β [95%CI]	P-value
Diagnosis method		0.981
Imaging modalities	-0.383 [-1.36 to 0.6]	
Surgery/Histology	-0.402 [-1.61 to 0.8]	
Concurrent pain symptoms		0.911
1-4	-0.044 [-1.43 to 1.34]	
5-6	-0.147 [-1.25 to 0.96]	
Infertility		0.472
Yes	0.399 [-1.55 to 2.35]	
No	-0.388 [-1.24 to 0.47]	
Fibroma		0.378
Yes	-0.341 [-3.08 to 2.4]	
No	1.727 [-1.83 to 5.29]	
Moderate depression		0.828
Yes	0.938 [-2.29 to 4.17]	
No	0.546 [-1.13 to 2.22]	
Moderate anxiety		0.359
Yes	-0.852 [-4.35 to 2.65]	
No	0.949 [-0.76 to 2.66]	
Central component of pain		0.041*
Yes	2.542 [-0.39 to 5.48]	
No	-0.718 [-1.77 to 0.33]	
Pain catastrophizing		0.782
Yes	-0.042 [-1.65 to 1.56]	
No	-0.303 [-1.18 to 0.57]	

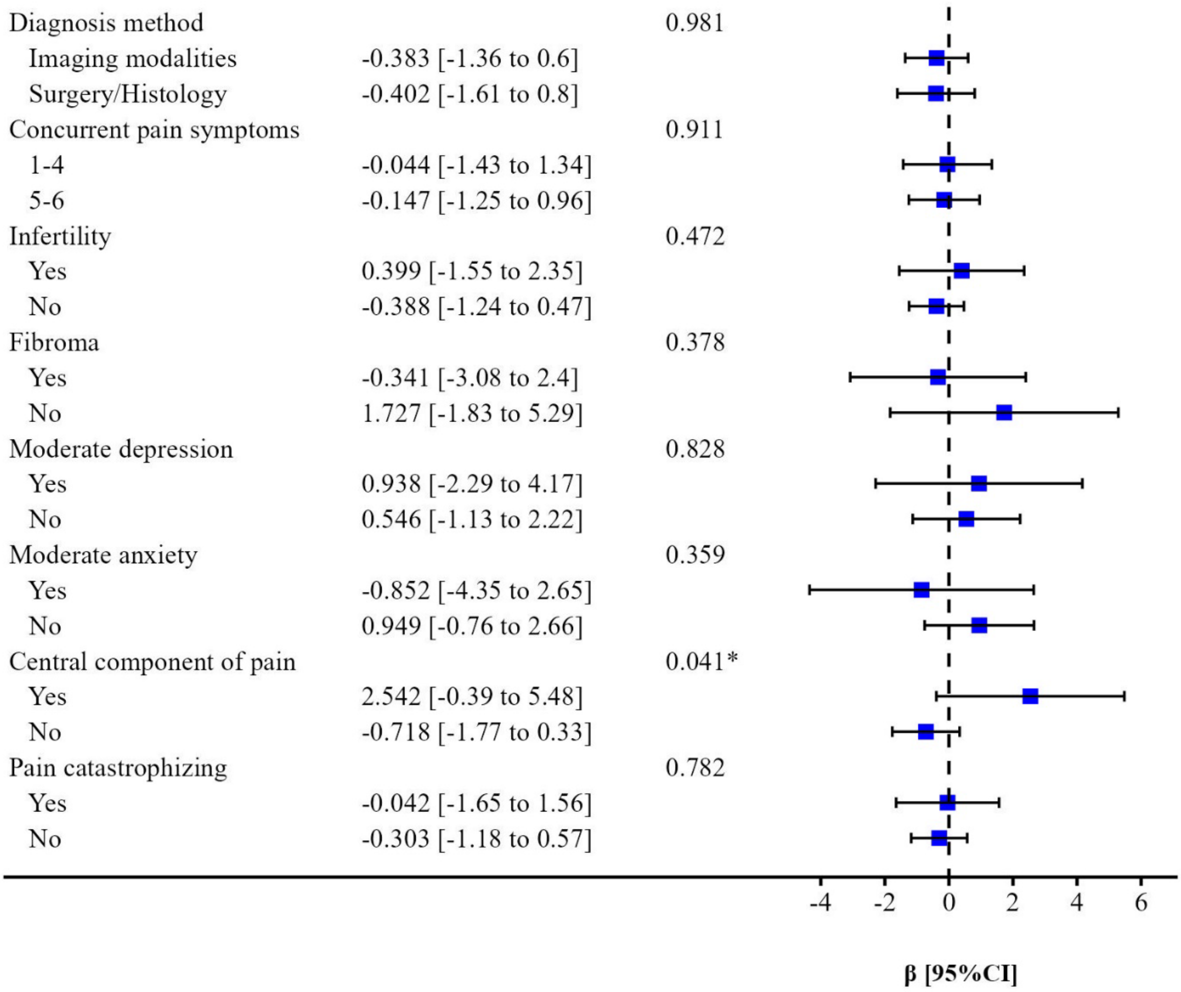

Figure S2. Subgroup analyses of mean difference in chronic pelvic pain intensity of patients with endometrioma compared to those with other types of endometriosis regarding potential modification factors.

Deep dyspareunia

Modification factor	β [95%CI]	P-value
Diagnosis method		0.939
Imaging modalities	-0.731 [-1.75 to 0.29]	
Surgery/Histology	-0.792 [-1.94 to 0.36]	
Concurrent pain symptoms		0.86
1-4	-0.353 [-1.66 to 0.95]	
5-6	-0.204 [-1.17 to 0.76]	
Infertility		0.582
Yes	-1.165 [-2.96 to 0.63]	
No	-0.613 [-1.43 to 0.2]	
Adenomyosis		0.398
Yes	-2.42 [-6.16 to 1.32]	
No	-0.727 [-1.95 to 0.5]	
Fibroma		0.784
Yes	-0.409 [-2.82 to 2]	
No	-0.876 [-3.38 to 1.62]	
Moderate depression		0.454
Yes	-1.673 [-4.32 to 0.97]	
No	-0.487 [-2.22 to 1.25]	
Moderate anxiety		0.945
Yes	-0.694 [-3.78 to 2.39]	
No	-0.816 [-2.51 to 0.88]	
Central component of pain		0.458
Yes	0.005 [-2.82 to 2.83]	
No	-1.162 [-2.3 to -0.02]	
Pain catastrophizing		0.117
Yes	0.318 [-1.2 to 1.84]	
No	-1.063 [-1.9 to -0.23]	

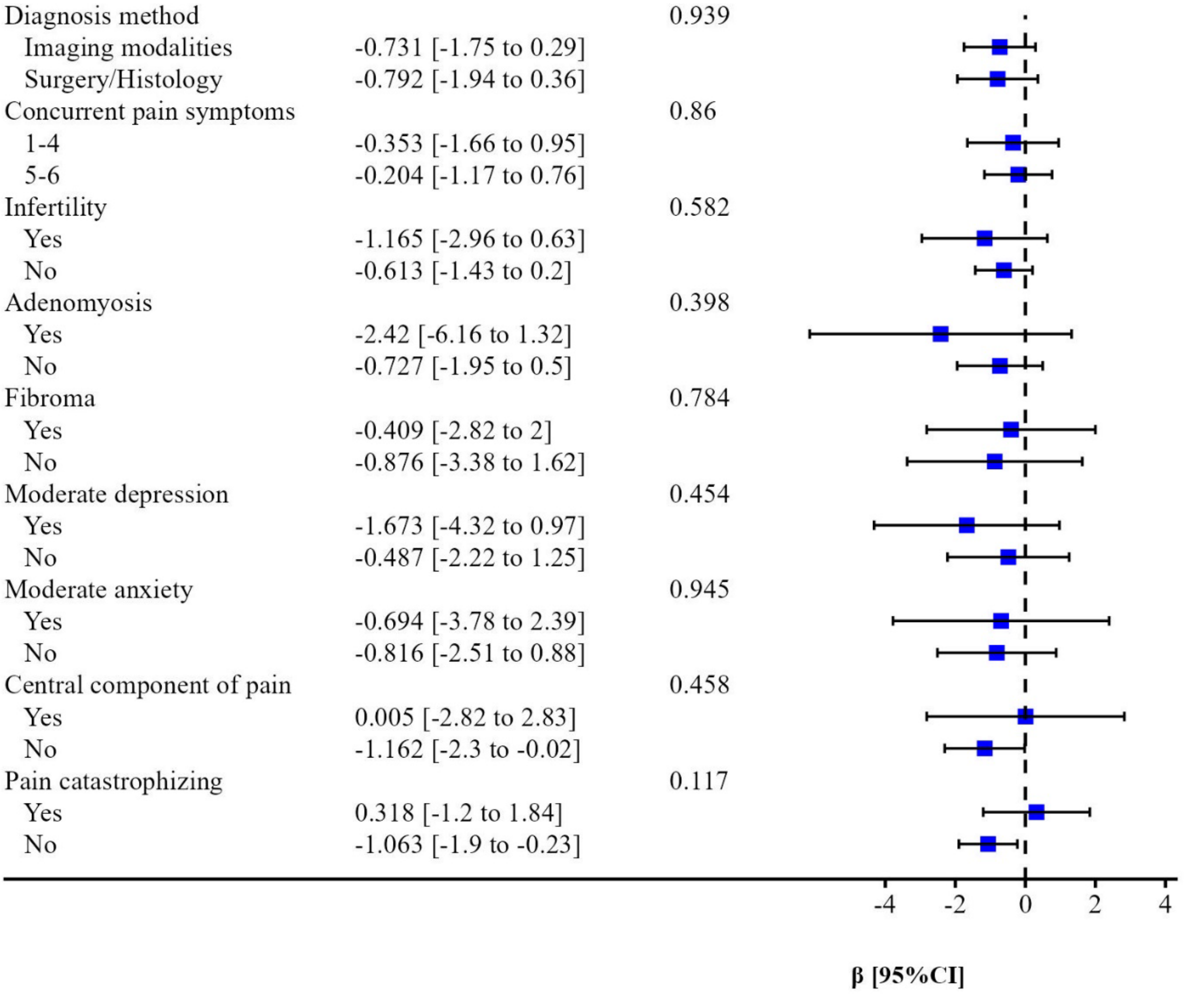

Figure S3. Subgroup analyses of mean difference in deep dyspareunia intensity of patients with endometrioma compared to those with other types of endometriosis regarding potential modification factors.

Lower back pain

Modification factor	β [95%CI]	P-value
Diagnosis method		0.86
Imaging modalities	-0.711 [-1.7 to 0.28]	
Surgery/Histology	-0.842 [-1.9 to 0.22]	
Concurrent pain symptoms		0.595
1-4	-1.032 [-2.39 to 0.32]	
5-6	-0.577 [-1.5 to 0.35]	
Infertility		0.645
Yes	-0.273 [-2.21 to 1.66]	
No	-0.763 [-1.53 to 0.01]	
Adenomyosis		0.144
Yes	-3.659 [-8.14 to 0.82]	
No	-0.143 [-1.51 to 1.22]	
Fibroma		0.568
Yes	-0.981 [-3.25 to 1.29]	
No	0.087 [-2.78 to 2.95]	
Moderate depression		0.936
Yes	-0.885 [-3.54 to 1.77]	
No	-1.012 [-2.75 to 0.72]	
Moderate anxiety		0.429
Yes	-1.95 [-4.74 to 0.84]	
No	-0.652 [-2.33 to 1.02]	
Central component of pain		0.054
Yes	-3.64 [-6.47 to -0.81]	
No	-0.657 [-1.73 to 0.41]	
Pain catastrophizing		0.171
Yes	0.327 [-1.13 to 1.78]	
No	-0.838 [-1.64 to -0.04]	

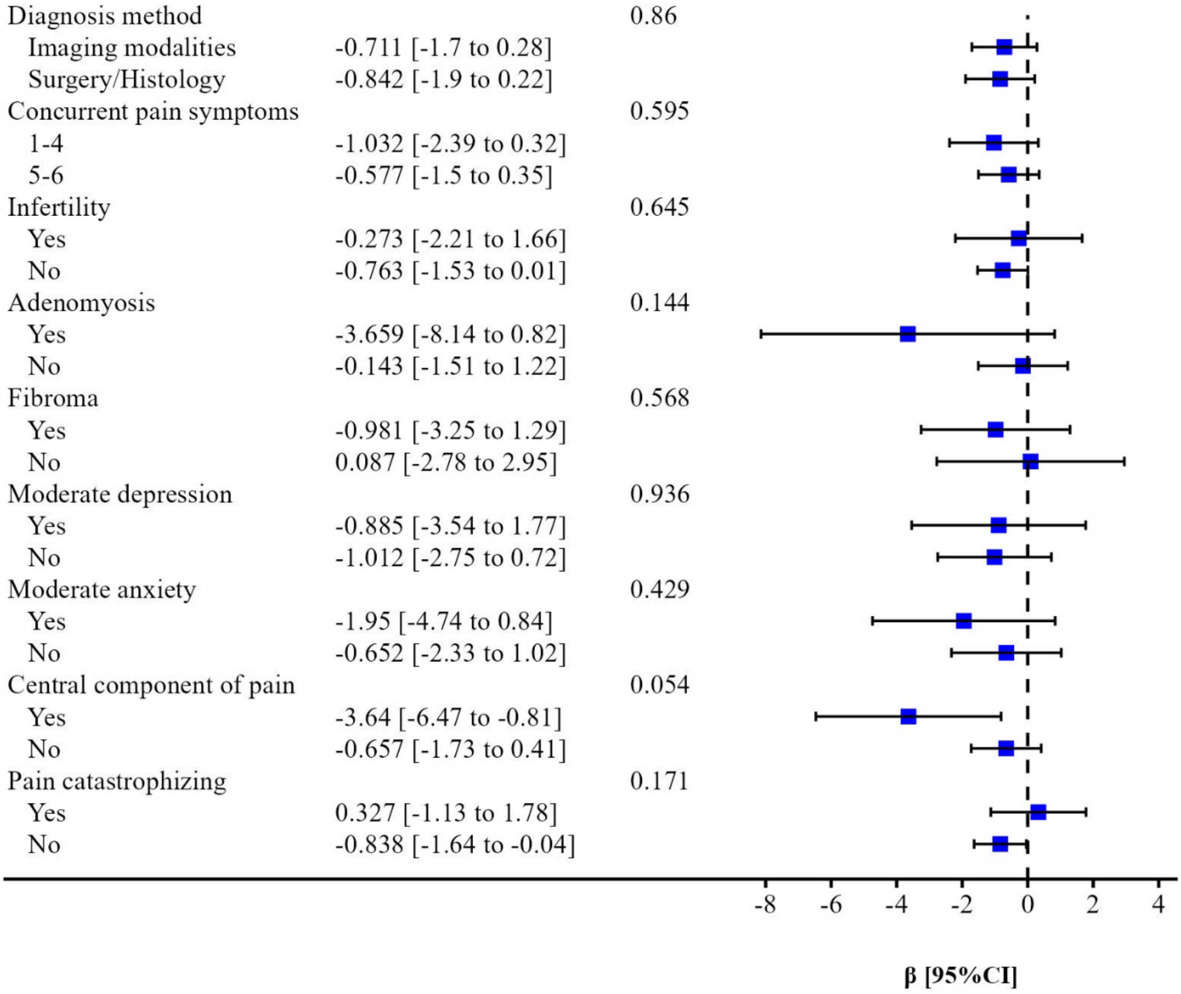

Figure S4. Subgroup analyses of mean difference in lower back pain intensity of patients with endometrioma compared to those with other types of endometriosis regarding potential modification factors.

Dysmenorrhea

Modification factor	β [95%CI]	P-value
Diagnosis method		0.564
Imaging modalities	-0.695 [-1.83 to 0.44]	
Surgery/Histology	-0.133 [-1.66 to 1.39]	
Concurrent pain symptoms		0.083
1-4	0.871 [-0.8 to 2.54]	
5-6	-0.928 [-2.08 to 0.23]	
Infertility		0.747
Yes	-0.131 [-2.47 to 2.21]	
No	-0.545 [-1.52 to 0.43]	
Fibroma		0.288
Yes	0.834 [-1.79 to 3.45]	
No	-1.94 [-6.67 to 2.79]	
Moderate depression		0.123
Yes	-2.021 [-5.04 to 0.99]	
No	0.657 [-1.25 to 2.56]	
Moderate anxiety		0.178
Yes	-2.218 [-5.75 to 1.31]	
No	0.358 [-1.45 to 2.17]	
Central component of pain		0.471
Yes	-1.514 [-4.41 to 1.39]	
No	-0.377 [-1.54 to 0.78]	
Pain catastrophizing		0.81
Yes	-0.598 [-2.61 to 1.41]	
No	-0.323 [-1.31 to 0.66]	

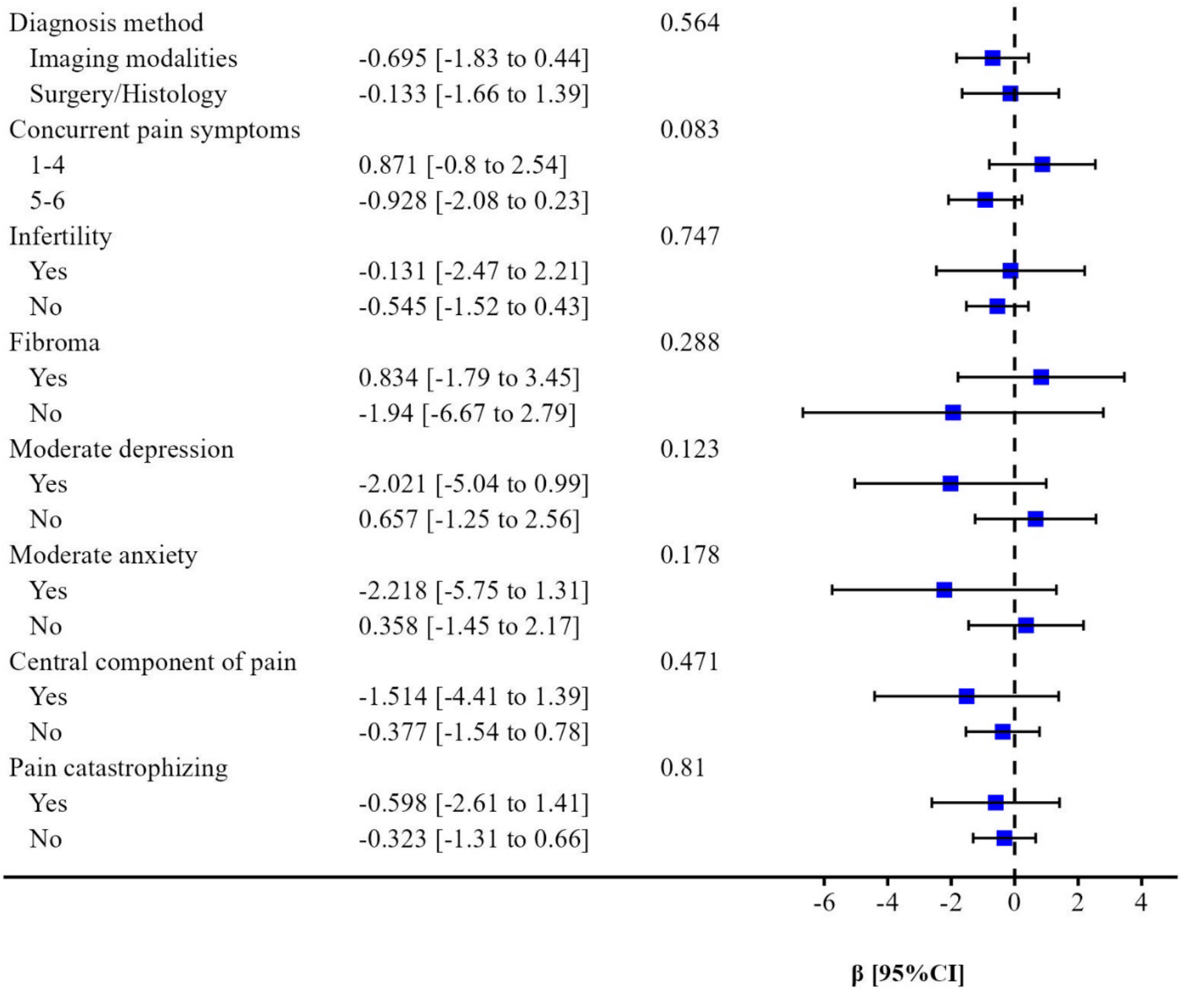

Figure S5. Subgroup analyses of mean difference in dysmenorrhea intensity of patients with endometrioma compared to those with other types of endometriosis regarding potential modification factors.

Dyschezia

Modification factor	β [95%CI]	P-value
Diagnosis method		0.549
Imaging modalities	0.545 [-0.79 to 1.88]	
Surgery/Histology	-0.038 [-1.36 to 1.29]	
Concurrent pain symptoms		0.54
1-4	-0.314 [-2.55 to 1.92]	
5-6	0.495 [-0.63 to 1.62]	
Infertility		0.659
Yes	0.744 [-1.46 to 2.94]	
No	0.193 [-0.83 to 1.21]	
Adenomyosis		0.714
Yes	-0.506 [-5.87 to 4.86]	
No	0.567 [-1.23 to 2.37]	
Fibroma		0.725
Yes	1.149 [-2.24 to 4.54]	
No	0.249 [-3.51 to 4.01]	
Moderate depression		0.942
Yes	-0.9 [-3.51 to 1.7]	
No	-1.024 [-3.07 to 1.03]	
Moderate anxiety		0.979
Yes	-0.958 [-3.96 to 2.05]	
No	-1.005 [-2.86 to 0.85]	
Central component of pain		0.847
Yes	-0.476 [-3.61 to 2.66]	
No	-0.139 [-1.4 to 1.12]	
Pain catastrophizing		0.925
Yes	0.312 [-1.44 to 2.06]	
No	0.213 [-0.85 to 1.28]	

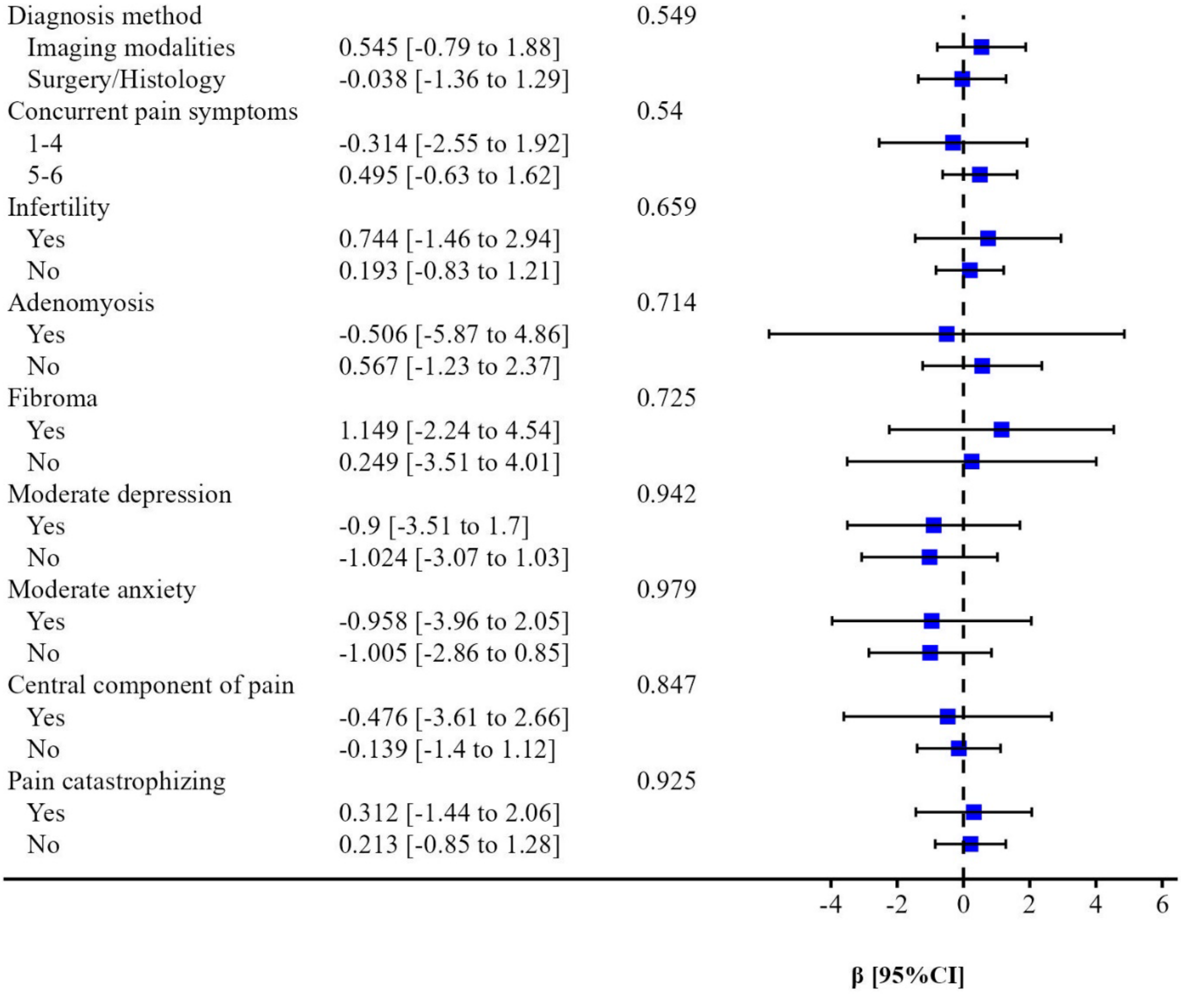

Figure S6. Subgroup analyses of mean difference in dyschezia intensity of patients with endometrioma compared to those with other types of endometriosis regarding potential modification factors.

Outcome	$N_{outcomes}/N_{group}$ E$_1$	$N_{outcomes}/N_{group}$ E$_0$	OR [95%CI]	p-value
Impaired quality of life	16 / 63	42 / 146	1.05 [0.5 to 2.19]	0.888
			0.84 [0.42 to 1.63]	0.618
Severe superficial dyspareunia	8 / 28	16 / 84	2.11 [0.6 to 7.43]	0.239
			1.7 [0.61 to 4.48]	0.291
Severe deep dyspareunia	27 / 55	77 / 120	0.52 [0.25 to 1.08]	0.08
			0.54 [0.28 to 1.03]	0.061
Severe dysmenorrhea	26 / 43	71 / 107	0.82 [0.38 to 1.79]	0.607
			0.78 [0.37 to 1.63]	0.495
Severe dyschezia	11 / 38	33 / 116	1.17 [0.47 to 2.82]	0.724
			1.02 [0.44 to 2.26]	0.953
Severe lower back pain	18 / 51	56 / 118	0.69 [0.33 to 1.42]	0.319
			0.6 [0.3 to 1.18]	0.145
Severe chronic pelvic pain	22 / 49	67 / 123	0.7 [0.34 to 1.42]	0.32
			0.68 [0.35 to 1.32]	0.258

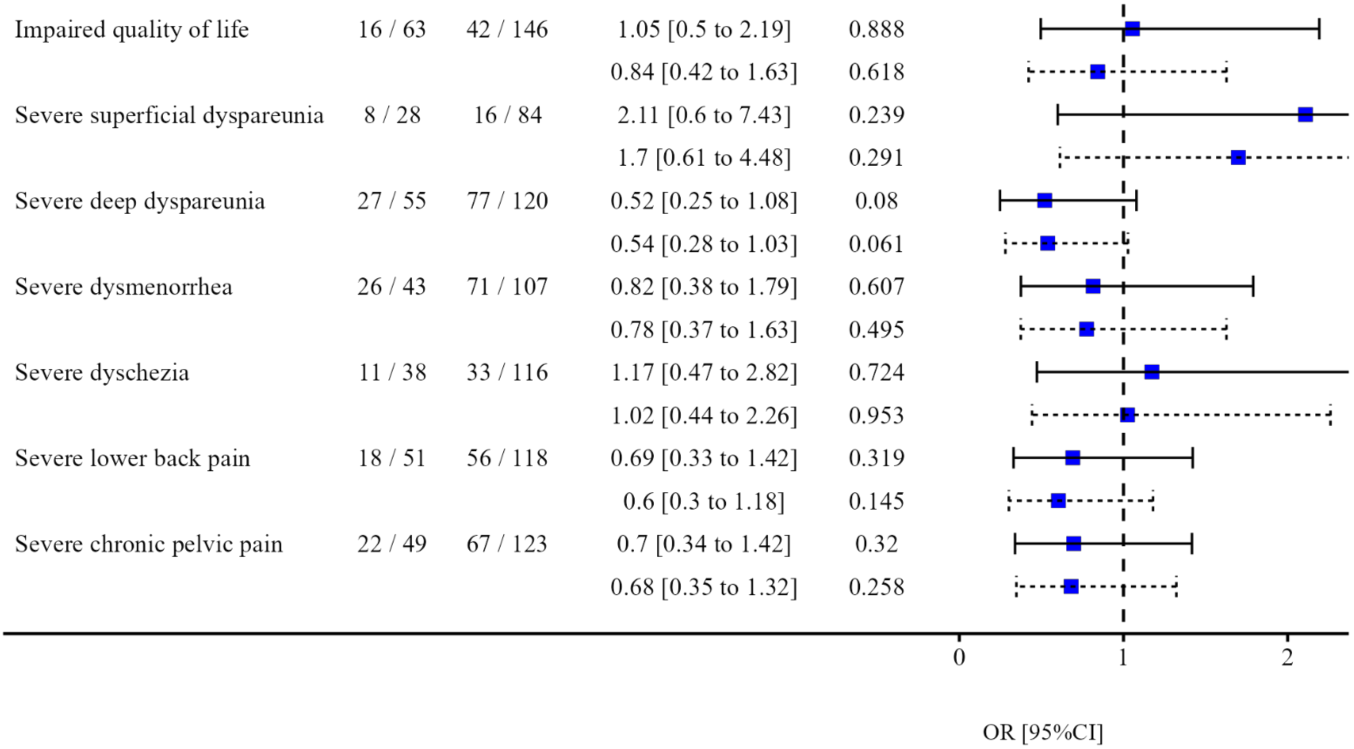

Figure S7. Impaired quality of life and severe pain symptoms of patients with endometriomas compared to those with other lesions of endometriosis.

Abbreviations: OR, odd radios; CI, Confidence interval.

Notes:

OR denotes the odds of a given outcome (impaired quality of life or severe pain symptom) occurring in the presence of endometriomas compared to the odds of that outcome in the absence of endometriomas.

$N_{outcomes}$ denotes the number of patients or individuals which had the outcome in a particular group whilst N_{group} denotes the total number of people in that group. "E_1" denotes endometrioma group, and "E_0", non-endometrioma group.

The quality of life is evaluated using the validated Endometriosis Health Profile-30 (EHP-30) questionnaire pain subscale. The EHP-30 score is categorized as impaired (score ≥ 75th centile of EHP-30 score population distribution) or best (score < 75th centile of EHP-30 score population distribution) quality of life.

Pain symptoms are evaluated using an 11-point numerical rating scale (NRS). Scores ranged from 0 (indicating no pain) to 10 (indicating worst pain imaginable). The pain NRS scores are categorized as mild-moderate (1-6) or severe (7-10).

The vertical black dashed line represents the null value of the odd ratios (1), indicating an odd ratio significantly different from 1 when its confidence interval does not include it (equivalent to $p < 0.05$).

The odds ratios are indicated by the blue squares (point estimates values) with their 95% confidence interval delimited by the black horizontal solid lines.

The models are adjusted for age, body mass index (kg/m^2), ethnicity, age of menarche, parity, education level, employment status, marital status, annual income, and hormone use in the last three months.
